# Complications following vaginal mesh procedures for stress urinary incontinence: an 8 year study of 92,246 women

**DOI:** 10.1038/s41598-017-11821-w

**Published:** 2017-09-20

**Authors:** Kim Keltie, Sohier Elneil, Ashwani Monga, Hannah Patrick, John Powell, Bruce Campbell, Andrew J. Sims

**Affiliations:** 1 Newcastle upon Tyne Hospitals NHS Foundation Trust, Newcastle upon Tyne, UK; 2 Institute of Cellular Medicine, Faculty of Medical Sciences, University of Newcastle upon Tyne, Newcastle upon Tyne, UK; 3 University College London Hospitals NHS Foundation Trust, London, UK; 4 University Hospital of Southampton NHS Foundation Trust, Southampton, UK; 5 National Institute for Health and Care Excellence, London, UK; 6 Nuffield Department of Primary Care Health Sciences, University of Oxford, Oxford, UK; 7 University of Exeter Medical School, University of Exeter, Exeter, UK

## Abstract

Complications of surgical mesh procedures have led to legal cases against manufacturers worldwide and to national inquiries about their safety. The aim of this study was to investigate the rate of adverse events of these procedures for stress urinary incontinence in England over 8 years. This was a retrospective cohort study of first-time tension-free vaginal tape (TVT), trans-obturator tape (TOT) or suprapubic sling (SS) surgical mesh procedures between April 2007 and March 2015. Cases were identified from the Hospital Episode Statistics database. Outcomes included number and type of procedures, including those potentially confounded by concomitant procedures, and frequency, nature and timing of complications. 92,246 first-time surgical mesh procedures (56,648 TVT, 34,704 TOT, 834 SS and 60 combinations) were identified, including 68,002 unconfounded procedures. Peri-procedural and 30-day complication rates in the unconfounded cohort were 2.4 [2.3–2.5]% and 1.7 [1.6–1.8]% respectively; 5.9 [5.7–6.1]% were readmitted at least once within 5 years for further mesh intervention or symptoms of complications, the highest risk being within the first 2 years. Complication rates were higher in the potentially confounded cohort. The complication rate within 5 years of the mesh procedure was 9.8 [9.6:10.0]% This evidence can inform future decision-making on this procedure.

## Introduction

Mesh insertion is the most common surgical procedure used to treat stress urinary incontinence (SUI) in women^1^, with 3.7 million meshes sold worldwide between 2005 and 2013^2^. However the safety of these procedures is the subject of international debate and scrutiny^3^ with court actions against mesh manufacturers underway in various countries, including Australia, Belgium, Canada, England, Israel, Italy, the Netherlands, Scotland, USA, and Venezuela^4^. In the USA, the FDA has proposed to raise the risk classification of urogynaecological meshes, requiring premarket notification and special controls^5^. In the UK, safety concerns have led to parliamentary questions^6^, a mandatory national audit^7^, a national campaign “Hear Our Voice”^8,9^, and in Scotland, an independent inquiry^10^. Some manufacturers have withdrawn their products from sale^11,12^.

Complications associated with mesh procedures for SUI include haemorrhage, organ perforation, mesh erosion, infection and pain^10,13^, which may require further surgery. However, there is uncertainty about the rates of complications during surgery and in the longer term, and concern that rates in everyday practice may be higher than previously identified^13,14^. Four systematic reviews have identified a lack of long-term outcome data^12,15–17^.

The primary aim of the study was to assess peri-procedural and post-procedural (within 30-days and long-term) outcomes following surgical mesh insertions for SUI using the administrative Hospital Episode Statistics (HES) database used in England^18^.

## Methods

### Data source

Records from English National Health Service (NHS) database of Admitted Patient Care (including day-cases)^19^ were extracted (on 26^th^ November 2015) from HES. All data extraction and analyses were carried out in accordance with relevant guidelines and regulations. Pseudonymised episode-level HES data were provided by NHS Digital to the Newcastle upon Tyne Hospitals NHS Foundation Trust under Data Sharing Framework Contract CON-313204-B3P1Y and Data Sharing Agreement HDIS-DSC-0109. In accordance with the terms of these agreements, only aggregated totals of patients and procedures are reported and no identifiable information was available for analysis. This research involved only previously collected, non-identifiable information and did not require review by a UK Research Ethics Committee. The study is registered on clinicaltrials.gov; NCT02850120.

### Study design and population

This was a retrospective cohort study of all women discharged from hospital in England between 1^st^ April 2007 and 31^st^ March 2015 after surgical mesh procedures for treatment of SUI. Unique pseudo-anonymised patient identifiers were extracted from finished episodes of care which contained at least one of the (Office of Population Censuses and Surveys Classification of Interventions and Procedures, OPCS4.7) procedure codes M53.3 (Introduction of tension-free vaginal tape, TVT), M53.6 (Introduction of trans-obturator tape, TOT) or M52.1 (Suprapubic sling operation, a mid-urethral sling introduced via a suprapubic approach, SS), Supplementary Table S1. All finished inpatient episodes during the study period for this population were extracted from HES.

### Data preparation & cleaning

Episodes were removed which were exact duplicates (of patient identifier, admission date and method, discharge date, destination and method, hospital, gender, age, all procedure codes and all diagnostic codes (International Statistical Classification of Diseases 10^th^ revision, ICD-10), Supplementary Table S1) and for patients with: an admission not coded as female; age missing or under 18 years; an invalid or missing admission method; a missing admission date; episodes after a reported date of death.

In this study we define *insertion* to mean the introduction of a mesh for the treatment of SUI, *repair* to mean a further procedure on a previously inserted mesh; *renewal* to mean a procedure to remove a previously inserted mesh and to replace it with a new mesh; and *removal* to mean the complete or partial removal of a previously inserted mesh. The OPCS-4 codes which correspond to each of these definitions are given in Supplementary Table S2.

In HES a spell is defined as one or more contiguous episodes within the same hospital admission. The ‘index spell’ for each patient was defined as the earliest admission within the study period including one or more procedure codes for surgical mesh insertion (M53.3, M53.6, M52.1); excluding procedure codes indicating pelvic organ prolapse surgery (P24.2, P24.5, P24.6, P23.6, P23.7, Q54.4, Q54.5, Q54.6), mesh repair, removal, renewal or subsequent mesh insertion^20^. Full details of codes and combinations used in this study are listed in Supplementary Tables S1 and S2.

Index spells without a coded diagnosis of incontinence (diagnostic codes N39.3, N39.4, R32) or without an implied diagnosis of incontinence (fitting of a urinary prosthesis: T83.1, T83.4, T83.5, T83.6, T83.8, T83.9, Z46.6) were excluded from analysis. Remaining eligible index spells were assumed to be first-time surgical mesh insertions for SUI.

Index spells were considered “unconfounded” if they included no concomitant procedures, or if concomitant procedures were considered unlikely to affect outcomes (Supplementary Table S3), or if they were rescue procedures associated with the mesh insertion procedure (Supplementary Table S4). The remaining index spells were considered “confounded” by concomitant procedures which potentially influenced outcomes. Two cohorts of patients were defined: those with an unconfounded index spell and those with a confounded index spell. Subgroups of patients within each cohort were defined by the type of mesh procedure (one of TVT, TOT or SS).

Analysis was conducted using the programming language R^21^. Analysis software and its saved output^22^ are available in Supplementary Files S5 & S6 respectively. HES data are available from NHS Digital via formal application^18^.

### Outcomes

Age, admission method, length of stay, frequency of endoscopic examination of the bladder and/or urethra (procedure codes: M45, M77), frequency of urinary or suprapubic catheter intervention (M30.2, M38.2, M47.1/4/8/9, M48.1) and rate of peri-procedural complications were reported for each mesh procedure^23^. Peri-procedural complications were classified, based on coding, as attributable to the procedure; attributable to the device; involving urinary symptoms (*e.g*. immediate acute urinary retention); or “other” (Supplementary Table S7).

All-cause readmissions, readmissions due to a complication^23^, or further mesh surgery (Supplementary Table S2) within 30 days of the index mesh insertion were recorded. 30-day readmissions for which the primary diagnostic code was not a complication code, or qualified by one, were considered “routine” readmissions.

Each patient was followed from their index procedure until 31^st^ March 2015 or their in-hospital death (if earlier). During follow-up particular events were recorded: in-hospital deaths; admissions due to complications of mesh implanted previously (using diagnostic codes intended for that purpose, from section T83 “complications of genitourinary prosthetic devices, implants or grafts”, or main codes qualified by codes from section Y73 “gastroenterology or urology devices associated with adverse incidents” (Supplementary Table S8)); admissions for further mesh surgery (excluding those due to a complication recorded within 30 days of the index procedure – to avoid double counting).

### Statistical analysis

Crude incidence rates for long-term complications were calculated as the number of mesh-related readmissions per 1000 person-years of follow-up. Instantaneous hazard rates were calculated using kernel-based smoothing^24,25^. Kaplan-Meier analysis was applied to the time from index procedure to the time of the first mesh-related readmission. Patients who suffered no mesh-related readmission and were alive at the end of the study were considered censored.

### Data availability

The Hospital Episode Statistics (HES) data used in this study were provided under license by NHS Digital to Newcastle Hospitals NHS Foundation Trust. The terms of the license do not permit the authors to make the data publicly available, but the data can be requested via formal application to NHS Digital using its online Data Access Request Service (http://content.digital.nhs.uk/dars).

The analytical software developed for this study is licensed by the authors under a Creative Commons Attribution-NonCommercial-ShareAlike 4.0 International License (Supplementary File S5).

### Details of ethics approval

Only aggregated totals of patients and procedures are reported. No identifiable information was available for analysis. No ethical approval was required.

## Results

### Participants

**Figure 1:**
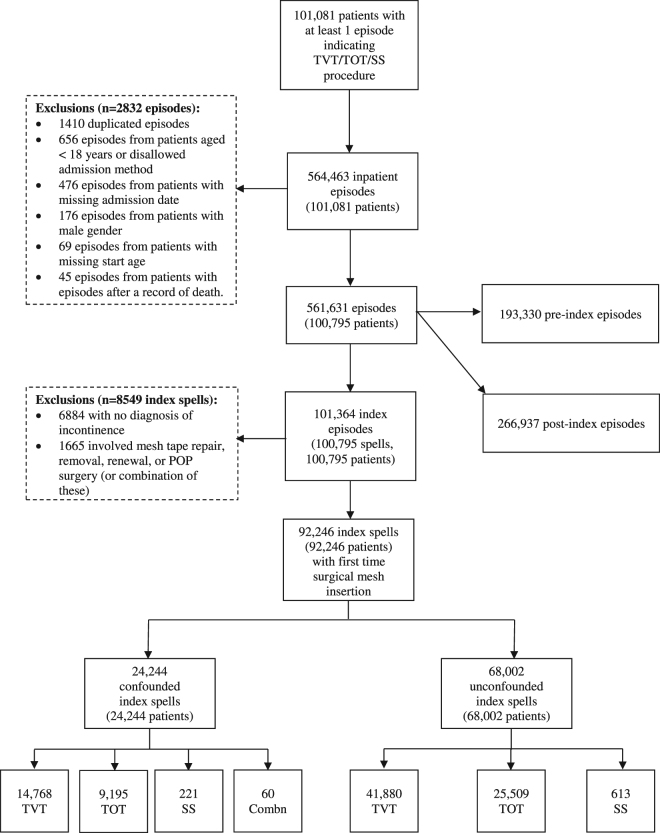
Flow diagram for study participants. “Combn” indicates that more than one type of surgical mesh was inserted.

During the study period, 101,081 patients had at least one surgical mesh insertion for SUI; 564,463 inpatient episodes of care were recorded for these patients. 2832 episodes were excluded from analysis because they were duplicates, or from patients with missing or ineligible demographic information (Fig. 1).

### Index procedures

An index spell was identified for each of the 100,795 remaining patients (101,364 episodes). 6884 admissions were excluded due to no documented diagnosis of SUI and 1665 were excluded due to evidence of earlier or concomitant mesh surgery (*e.g*. surgical mesh repairs, removals or pelvic organ prolapse surgery). Of the remaining 92,246 index spells, 68,002 (73.7%) were unconfounded and 24,244 (26.3%) were confounded.

**Figure 2:**
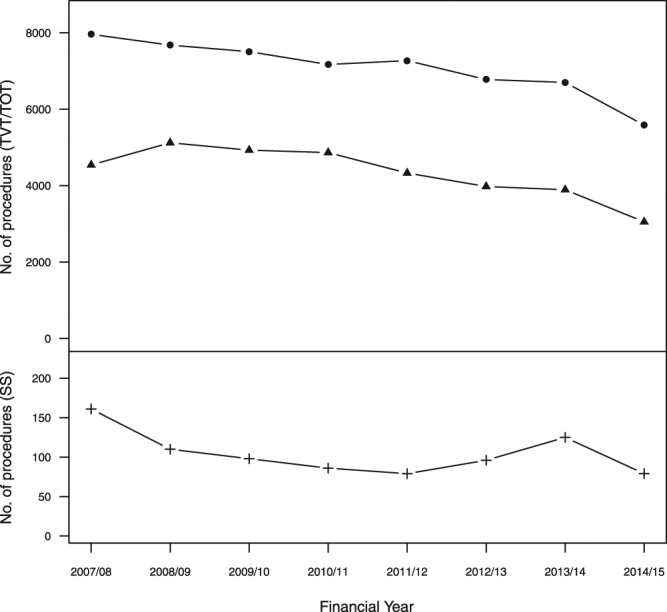
Annual activity of surgical mesh insertions for SUI in England, 2007/8 to 2014/15 (Key to symbols: ● = TVT; ▲ = TOT; += SS).

Yearly totals of index surgical mesh insertions for SUI are shown in Fig. 2. These were carried out in the majority of NHS trusts providing acute care in England, and almost all admissions were elective (Table 1). One quarter (24.7%, 22,747/92,246) included clinical codes for endoscopy of the bladder or urethra. The median length of stay for unconfounded surgical mesh insertions was one day, with 9537/68,002 (14.0%) staying longer than one night (maximum of 33 days), and two days for insertions done with concomitant procedures, with 15,853/24,244 (65.4%) staying longer than one night (maximum of 90 days).

**Table 1:** Outcomes from index admissions to introduce tension-free vaginal (TVT), trans-obturator (TOT) and suprapubic sling (SS) surgical mesh products in the treatment of stress urinary incontinence.

	Unconfounded cohort^†^			Confounded cohort^‡^		
	TVT	TOT	SS	TVT	TOT	SS
Patients	41,880	25,509	613	14,768	9195	221
Elective admissions (%)	41,831 (99.9)	25,490 (99.9)	611 (99.7)	14,722 (99.7)	9180 (99.8)	220 (99.5)
Number of hospitals	175	170	67	172	158	54
Age, years: median (lower quartile, upper quartile)	50 (44, 60)	51 (44, 61)	52 (45, 62)	51 (44, 62)	51 (45, 63)	50 (41, 63)
Endoscopic examination of bladder/urethra (%)	11,466 (27.4)	5477 (21.5)	125 (20.4)	3781 (25.6)	1825 (19.8)	53 (24.0)
Catheterisations (%)	928 (2.2)	444 (1.7)	11 (1.8)	759 (5.1)	324 (3.5)	27 (12.2)
Length of stay, days median (lower quartile, upper quartile)	1 (0, 1)	1 (0, 1)	2 (0, 4)	2 (1, 3)	2 (1, 3)	3 (2, 7)
In-hospital deaths	0	0	0	0	0	0
Procedures with in-hospital complications (%)	1232 (2.9)	356 (1.4)	30 (4.9)	880 (6.0)	342 (3.7)	23 (10.4)
*Complication attribution:*						
*- Procedural (%)†*	*656 (53.2)*	*90 (25.3)*	*19 (63.3)*	*453 (51.5)*	*161 (47*.*1)*	*17 (73*.*9)*
*- Device (%)†*	*83 (6.7)*	*28 (7.9)*	*4 (13.3)*	*62 (7.0)*	*21 (6*.*1)*	0
- Complications with urinary symptoms (%)*	*416 (33.8)*	*172 (48.3)*	*4 (13.3)*	*210 (23.3)*	*99 (28*.*9)*	*2 (8*.*7)*
- Other complications (%)*	*140 (11.4)*	*76 (21.3)*	*6 (20.0)*	*204 (23.9)*	*76 (22*.*2)*	*6 (26*.*1)*

Key: *Percentage of procedures with a complication, ^†^Index admissions without concomitant procedures which may influence outcomes, ^‡^Index admissions with concomitant procedures which may influence outcomes.

### Complications during index spells

Complications were reported in 2.4 [95% CI 2.3–2.5] % of unconfounded and 5.2 [95% CI 4.9–5.5] % of confounded index spells. T81 “Complications of procedures, not elsewhere classified”, and R33 “Retention of urine” were the two most frequently reported complications in both cohorts (Supplementary Tables S9 and S10), accounting for 72.4% (2202/3044) of the total.

### Outcomes within 30-days

In the unconfounded cohort, 7.1% (4850/68,002) were re-admitted within 30 days on 5904 occasions. Almost a quarter (23.5%, 1137) were admitted (on 1273 occasions) due to a complication, with 171 requiring further mesh surgery. Most (76.9%) of the routine readmissions within 30 days involved no further surgery (Supplementary Table S11). In the confounded cohort, 2345 (9.7%) women were re-admitted within 30 days; including 738 (31.5%) whose readmission was due to a complication (Supplementary Table S12). The 30-day complication rate for the unconfounded cohort was 1.7 [95% CI 1.6–1.8] % and 3.0 [95% CI 2.8–3.3] % for the confounded cohort.

### Longitudinal outcomes

**Figure 3:**
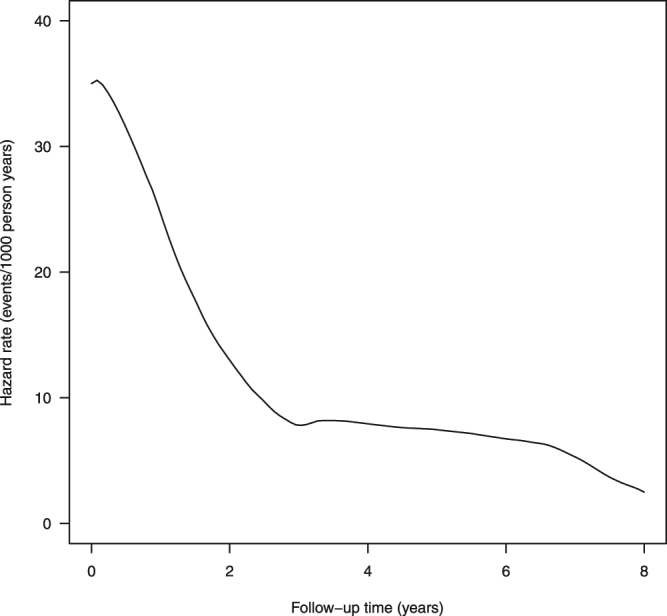
Hazard of readmission for further mesh surgery or due to complications from previous mesh surgery (in units of events per 1000 patient years) for those having surgical mesh insertion in the absence of concomitant procedures likely to influence outcomes.

The aggregate follow-up in the unconfounded cohort was 286,273 years (mean 4.2 years; 90.2% were followed >1 year; 66.5% >3 years; 40.9% >5 years, 13.6% >7 years). The frequencies of mesh-related readmissions, either for further mesh surgery or for symptoms indicating a complication of a previous mesh, are shown in Table 2. Table 3 shows the unadjusted number of readmissions and crude incidence rates for the unconfounded cohort (overall 16.0 per 1000 patient-years of follow-up); Fig. 3 shows the estimated hazard rate of readmission during 8 years after a first-time mesh implant for SUI.

**Table 2:** The total number of patients (%, percentage of cohort) who had a trans-vaginal tape (TVT), transobturator tape (TOT) or suprapubic sling (SS) mesh insertion (in the absence of concomitant procedures) who were re-admitted during the study period for further mesh surgery or due to complications from previous mesh surgery. Results are uncorrected for censoring.

Procedure type	Number of readmissions				Maximum number of readmissions
	0	1	2	3+	
TVT	39,632 (94.6)	1737 (4.1)	375 (0.9)	136 (0.3)	6
TOT	24,254 (95.1)	1017 (4.0)	174 (0.7)	64 (0.3)	6
SS	574 (93.6)	34 (5.5)	4 (0.7)	1 (0.2)	3
All (combined)	64,460 (94.8)	2788 (4.1)	553 (0.8)	201 (0.3)	6

For example, 2248 of 41,880 (5.4%) patients who had a TVT mesh inserted were re-admitted at least once during the period of follow-up (mean follow-up of 4.2 years).

**Table 3:** Reasons and frequency of readmissions during follow-up. The total number (%, percentage of cohort) of patients readmitted is also given (some patients being readmitted on multiple occasions).

Total number of patients in cohort (total duration of follow-up)	TVT		TOT		SS		All	
	41,880 (175,284 patient years)		25,509 (108,339 patient years)		613 (2650 patient years)		68,002 (286,273 patient years)	
	Total	Patients	Total	Patients	Total	Patients	Total	Patients
In-hospital deaths	—	345 (0.8%)	—	204 (0.8%)	—	3 (0.5%)	—	552 (0.8%)
**Readmissions** for further surgery	2476	2016 (4.8%)	1370	1161 (4.6%)	40	36 (5.9%)	3886	3213 (4.7%)
*- removal*	*1277*	*1112 (2.7%)*	*541*	486 (1.9%)	11	10 (1.6%)	1829	1608 (2.4%)
*- repair*	*459*	*435 (1.0%)*	*240*	227 (0.9%)	6	5 (0.8%)	705	657 (1.0%)
*- insertion*	*831*	*812 (1.9%)*	*630*	615 (2.4%)	25	24 (3.9%)	1486	1451 (2.1%)
*- renewal*	*5*	*5 (0.0%)*	*1*	1 (0.0%)	0	0 (0.0%)	6	6 (0.0%)
**Readmissions** for complications from mesh surgery	1389	1047 (2.5%)	607	484 (1.9%)	12	10 (1.6%)	2008	1541 (2.3%)
**Readmissions** for complications *or* further surgery	2950	2248 (5.4%)	1580	1255 (4.9%)	45	39 (6.4%)	4575	3542 (5.2%)
**Readmissions**/1000 person years:								
- further mesh surgery	14.1	—	12.7	—	15.1	—	13.6	—
- complications of mesh surgery	7.9	—	5.6	—	4.5	—	7.0	—
- complications *or* further surgery	16.8	—	14.6	—	17.0	—	16.0	—
**Patients** free from further surgery or admission for complications after 5 years [95% CI] %	—	93.9 [93.7–94.2]	—	94.4 [94.1–94.8]	—	92.5 [90.1–94.9]	—	94.1 [93.9–94.3]

**Figure 4:**
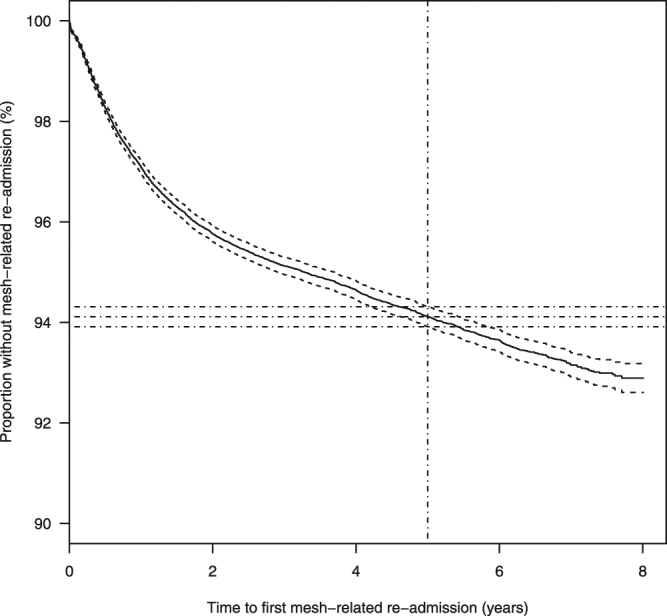
Kaplan-Meier curve for time to first readmission for further mesh surgery or for complications from previous mesh surgery (solid lines) and 95% confidence limits (dashed lines) for those having surgical mesh insertion in the absence of concomitant procedures likely to influence outcomes. Dash-dot lines indicate the proportions of patients free from mesh-related admission at 5 years (with 95% confidence intervals).

Adjusting for different lengths of follow-up, 5.9 [95% CI 5.7 to 6.1] % of women having unconfounded mesh procedures were readmitted for a further mesh intervention or for symptoms of mesh complications within 5 years of their first-time mesh procedure, Fig. 4 (Supplementary Figs S13–S15).

In the unconfounded cohort, 1212 patients (1.8%) were readmitted for complications of earlier mesh surgery (based on their diagnostic codes) *and* for further mesh surgery (based on their procedure codes), in some cases during the same admission. Overall, 3541 (5.2%) were readmitted for either reason (Supplementary Table S16). The majority of patients readmitted for complications of an earlier mesh surgery (1212/1541, 78.7%) required further mesh surgery.

The aggregate follow-up in the confounded cohort was 102,870 years (mean 4.3 years). Frequencies of mesh-related readmissions, total number of readmissions and crude incidence rates are reported in Supplementary Tables S17 and S18. The estimated hazard rate of readmission during 8 years is shown in Supplementary Fig. S19. Adjusting for different lengths of follow-up, 6.4 [95% CI 6.1 to 6.8] % of women undergoing potentially confounded mesh procedures were readmitted for a further mesh intervention or for symptoms of mesh complication within 5 years of their first-time mesh procedure (Supplementary Fig. S20).

The proportion of patients experiencing a complication during the mesh insertion procedure, within 30 days or within 5 years, was 9.8 [9.6:10.0]%: 8.7% (5915/68,002) in the unconfounded cohort and 12.8% (3105/24,244) in the confounded cohort.

## Discussion

We estimated that 9.8% of patients undergoing surgical mesh insertion for SUI experience a complication peri-procedurally, within 30-days or within 5 years. The risk of readmission is highest within the first 2 years and some women required up to 6 mesh-related readmissions (mean follow-up of 4.2 years). We estimate that 5.9 [5.7–6.1] % of women (6.4 [6.1–6.8] % in the confounded cohort) required readmission for further mesh intervention within 5 years of their index procedure, more than double the cumulative incidence rate of mesh revision and removal (2.3% at 5 years) reported previously^26^. These findings provide evidence in this area of concern particularly considering that the prevalence of stress urinary incontinence is between 10–40% in community-dwelling women and higher in the elderly^27^.

The NHS England Mesh Working Group found insufficient evidence to determine the extent of longer term complications^14^. An observational cohort study used insurance claims from 188,454 women over a 9-year period, but included approximately 98,000 women with concomitant POP surgery^28^. The study with the longest follow-up was a prospective multi-centre cohort study of 90 women following TVT mesh insertion for a mean of 7.5 years^29^. Although the mean follow-up of our study was 4.2 years, approximately 9240 women (13.6%) were followed for over 7 years.

An independent review commissioned by the Scottish government also analysed administrative data, reported as an interim results^10^ and as a full paper^30^. The proportions of unconfounded TVT, TOT and SS patients experiencing peri-procedural complications (2.9%, 1.4% and 4.9% respectively) reported here are lower than those estimated by the Scottish Inquiry (4%, 2% and 6%) and reported in their full paper^30^. This may be a consequence of the different methods used to identify complications. We used the method recommended by the NHS Classification Service^23^. The Scottish Inquiry considered the presence of prospectively defined diagnostic codes (grouped as haemorrhage, infection, pain and procedure-related complications) to indicate a complication, but these included other diagnoses (*e.g*. ICD-10: N94.1 “Dyspareunia” and R52.2 “Other chronic pain”) which, without a qualifying supplementary code, may indicate comorbidities rather than procedural complications.

The Scottish Inquiry reported crude readmission rates^30^ almost double that of our study. This may be explained by the different methodologies. For example, the Scottish Inquiry included pelvic organ prolapse (POP) procedures, additional non-mesh intervention and any diagnosis of general infection, chronic pain, and haemorrhage as relevant readmissions. However, we found it difficult to attribute these to an earlier mesh insertion: subsequent POP or non-mesh procedures may indicate efficacy rather than safety. Furthermore the presence of diagnostic codes for infection or pain were not necessarily due to a previous mesh insertion. In summary our methods were more specific, but perhaps less sensitive than that applied by the Scottish Inquiry, with our study providing a more conservative but robust estimate of long-term complications^31^.

This is the largest study to date of SUI mesh procedures (92,244), with 100% coverage of NHS patients (including private patients treated in an NHS setting) in England over an 8-year period. The British Society of Urogynaecology (BSUG) established a database to record data relating to any anti-incontinence and/or prolapse procedure, including those conducted in a private setting. However results from this audit are, as yet, unpublished^32^.

Case ascertainment was optimised by using recommended coding practice to identify peri-procedural and 30-day complications, and choosing codes directly attributable to mesh complications to identify long-term events. Around two thirds of first-time mesh insertions for SUI had no concomitant procedures likely to affect outcomes; these cases were analysed separately from potentially confounded ones, increasing confidence in attributing complications to mesh intervention. A limitation is the dependence on clinical coding. For example, outcomes were not compared between TVT, TOT and SS, or between the confounded and unconfounded cohorts because severity of incontinence is not available from ICD-10 diagnostic codes for baseline matching. Analysis of procedural complications was limited by lack of detail (*e.g*. “T81: Complications of procedures, not elsewhere classified” was the most common complication reported peri-procedurally); the introduction of additional complication codes in ICD-11, expected in 2018^33^, will increase the utility of future administrative data to assess the safety of interventional procedures. The outcome measures chosen reflected the concerns expressed by patient representatives, and were available from the HES database of inpatient and day case episodes; however complications managed entirely in an outpatient setting were not captured in this study. Because emigration and out-of-hospital death are not recorded in HES more women were assumed to be at risk, in the long-term analysis, than was actually the case. Both these limitations suggest that the true hazard rates may be higher than reported. The accuracy of clinical coding is an important factor to consider in the potential limitations of this study. The accuracy of coding in HES data, as in all routinely collected datasets derived from administrative sources, is often questioned, however the Audit Commission in England have shown that HES coding accuracy has improved over time, when auditing Payment by Results (PbR) assurance programme^34^.

## Conclusions

This is the largest study to date of surgical mesh insertions for SUI. It includes all NHS patients in England over an 8-year period. We estimate that 9.8% of patients undergoing surgical mesh insertion for SUI experienced a complication peri-procedurally, within 30-days or within 5 years of the initial mesh insertion procedure. This is likely a lower estimate of the true incidence. Given concerns about the safety of these procedures, this study provides robust data to inform both individual decision-making and national guidance.

## Electronic supplementary material

Supplementary Information
