# Supplementary material for: Complications following vaginal mesh procedures for stress urinary incontinence: an 8 year study of 92,246 women
Source: Sci Rep. 2017 Sep 20;7:12015. doi: 10.1038/s41598-017-11821-w (PMC5607307; doi:10.1038/s41598-017-11821-w)

Complications following vaginal mesh procedures for stress urinary incontinence: an 8 year study of 92,246 women

Kim Keltie, Sohier Elneil, Ashwani Monga, Hannah Patrick,
John Powell, Bruce Campbell, Andrew J Sims

Supplementary Information

Supplementary Table S1: List of procedure (OPCS-4) and diagnosis (ICD10) codes used during analysis ('.x' represents any number from 0 to 9 indicating that the whole chapter is used). OPCS-4 codes can appear in any of the 24 procedure fields in HES; ICD10 codes can appear in any of the 20 diagnosis fields in HES.

OPCS-4 Code	*OPCS-4 Description*
M30.2	Endoscopic catheterisation of ureter
M38.2	Cystostomy and insertion of suprapubic tube into bladder
M45.x	Diagnostic endoscopic examination of bladder
M47.1	Urethral irrigation of bladder
M47.4	Urodynamic studies using catheter
M47.8	Urethral catheterisation of bladder – other specified
M47.9	Urethral catheterisation of bladder – unspecified
M48.1	Suprapubic aspiration of bladder
M52.1	Suprapubic sling operation
M52.8	Abdominal operations to support outlet of female bladder – other specified
M53.3	Introduction of tension-free vaginal tape
M53.4	Total removal of tension-free vaginal tape
M53.5	Partial removal of tension-free vaginal tape
M53.6	Introduction of transobturator tape
M53.7	Removal of transobturator tape
M53.8	Vaginal operations to support outlet of female bladder – other specified
M77.x	Diagnostic endoscopic examination of urethra
P23.6	Anterior colporrhaphy with mesh reinforcement
P23.7	Posterior colporrhaphy with mesh reinforcement
P24.2	Sacrocolpopexy
P24.5	Repair of vault of vagina with mesh using abdominal approach
P24.6	Repair of vault of vagina with mesh using vaginal approach
Q54.4	Suspension of uterus using mesh, not elsewhere classified
Q54.5	Sacrohysteropexy
Q54.6	Infracoccygeal hysteropexy
Y03.1	Maintenance of prosthesis in organ, Not Otherwise Classifiable
Y03.2	Renewal of prosthesis in organ Not Otherwise Classifiable
Y03.3	Correction of displacement of prosthesis, Not Otherwise Classifiable
Y03.4	Other resiting of prosthesis in organ, Not Otherwise Classifiable
Y03.6	Adjustment to prosthesis in organ, Not Otherwise Classifiable
Y03.7	Removal of prosthesis from organ, Not Otherwise Classifiable
Y03.8	Other specified attention to prosthesis in organ, Not Otherwise Classifiable
Y03.9	Unspecified attention to prosthesis in organ, Not Otherwise Classifiable
Y26.4	Removal of other repair material from organ Not Otherwise Classifiable
Y26.5	Other attention to repair of organ, Not Otherwise Classifiable
Y71.2	Secondary operations, Not Otherwise Classifiable
Y71.3	Revisional operations, Not Otherwise Classifiable
Y71.6	Second revisional operation, Not Otherwise Classifiable
Y71.7	Third or greater revisional operation, Not Otherwise Classifiable
ICD10 Code	*ICD10 Description*
N39.3	Stress incontinence
N39.4	Other specified urinary incontinence
N93.x	Other abnormal uterine and vaginal bleeding
N94.1	Other abnormal uterine and vaginal bleeding
N99.8	Other postprocedural disorders of genitourinary system
N99.9	Postprocedural disorder of genitourinary system, unspecified
R10.0	Abdominal and pelvic pain – acute abdomen
R10.2	Pelvic and perineal pain
R10.3	Pain localized to other parts of lower abdomen
R10.4	Other and unspecified abdominal pain

continued . . .

. . . continued

ICD10 Code	*ICD10 Description*
R30.x	Pain associated with micturition
R31.x	Unspecified haematuria
R32.x	Unspecified urinary incontinence
R33.x	Retention of urine
R34.x	Anuria and oliguria
R35.x	Polyuria
R36.x	Urethral discharge
R39.x	Other symptoms and signs involving the urinary system
S34.5	Injury of lumbar, sacral and pelvic sympathetic nerves
S34.6	Injury of peripheral nerve(s) of abdomen, lower back and pelvis
S34.8	Injury of other and unspecified nerves at abdomen, lower back and pelvis level
S35.7	Injury of multiple blood vessels at abdomen, lower back and pelvis level
S35.8	Injury of other blood vessels at abdomen, lower back and pelvis level
S35.9	Injury of unspecified blood vessel at abdomen, lower back and pelvis level
S36.5	Injury of colon
S36.7	Injury of multiple intra-abdominal organs
S36.8	Injury of other intra-abdominal organs
S36.9	Injury of unspecified intra-abdominal organ
S37.0	Injury of kidney
S37.1	Injury of ureter
S37.2	Injury of bladder
S37.3	Injury of urethra
S37.7	Injury of multiple pelvic organs
S37.8	Injury of other pelvic organs
S37.9	Injury of unspecified pelvic organ
S39.x	Other and unspecified injuries of abdomen, lower back and pelvis
T81.x	Complications of procedures, not elsewhere classified
T83.1	Mechanical complication of other urinary devices and implants
T83.4	Mechanical complication of other prosthetic devices, implants and grafts in genital tract
T83.5	Infection and inflammatory reaction due to prosthetic device, implant and graft in urinary system
T83.6	Infection and inflammatory reaction due to prosthetic device, implant and graft in genital tract
T83.8	Other complications of genitourinary prosthetic devices, implants and grafts
T83.9	Unspecified complication of genitourinary prosthetic device, implant and graft
T85.6	Mechanical complication of other specified internal prosthetic devices, implants and grafts
T85.7	Infection and inflammatory reaction due to other internal prosthetic devices, implants and grafts
T85.8	Other complications of internal prosthetic devices, implants and grafts, not elsewhere classified
T85.9	Unspecified complication of internal prosthetic device, implant and graft
Y60.0	During surgical operation
Y60.8	During other surgical and medical care
Y73.2	Gastroenterology and urology devices associated with adverse incidents - Prosthetic and other implants, materials and accessory devices
Y73.3	Gastroenterology and urology devices associated with adverse incidents - Surgical instruments, materials and devices (including sutures)
Y83.1	Surgical operation with implant of artificial internal device
Z46.6	Fitting and adjustment of urinary device

Supplementary Table S2: Definition of surgical mesh removals, repairs, renewals and insertions using OPCS-4 procedure codes.

Surgical mesh procedure	*OPCS-4 procedure code combinations*
Removal	Main codes (M53.4 or M53.5 or M53.7), or main codes (M53.3 or M53.6 or M52.1 or M53.8* or M52.8*) qualified by (Y03.7 or Y26.4) supplementary codes.
Repair	Main codes (M53.3, M53.6, M52.1, M53.8*, M52.8*) qualified by (Y71.2 or Y71.3 or Y71.6 or Y71.7 or Y26.5 or Y03.1 or Y03.3 or Y03.4 or Y03.6 or Y03.8 or Y03.9) supplementary codes.
Renewal	Main codes (M53.3, M53.6, M52.1, M53.8*, M52.8*) qualified by Y03.2 supplementary code.
Insertion	The supplementary OPCS code, Y03.2, used to identify renewals is not well utilised (as confirmed by survey of clinical coders across England). Therefore patients with repeated occurrences of any insertion main code (M53.3, M53.6, M52.1) which were not followed by the supplementary codes defined in the repairs, removals and renewals, were deemed to have multiple surgical mesh insertion procedures.

* codes used only during post-procedural analysis

Supplementary Table S3: List of OPCS-4 procedure codes considered not to influence outcomes if performed during an index procedure ('.x' represents any number from 0 to 9 indicating that the whole chapter is used)

Category	*OPCS-4 codes*	*Description*
Diagnostic endoscopic examinations	A55.x	Diagnostic spinal puncture
	L72.x	Diagnostic transluminal operations on other artery
	L95.x	Diagnostic transluminal operations on vein
	M11.x	Diagnostic endoscopic examination of kidney
	M30.x	Diagnostic endoscopic examination of ureter
	M45.x	Diagnostic endoscopic examination of bladder
	M77.x	Diagnostic endoscopic examination of urethra
	M85.x	Diagnostic endoscopic examination of urinary diversion
	Q18.x	Diagnostic endoscopic examination of uterus
	Q39.x	Diagnostic endoscopic examination of fallopian tube
	Q50.x	Diagnostic endoscopic examination of ovary
Diagnostic imaging, testing and rehabilitation methods	A84.3	Nerve conduction studies
	H44.4	Examination of rectum under anaesthetic
	M49.6	Micturating cystography
	P27.3	Colposcopy of vagina
	Q55.x	Other examination of female genital tract
	U01.x-U21.x	Diagnostic imaging
	U22.x-U40.x	Diagnostic testing
	U50.x-U54.x	Rehabilitation
	Y93.x	Gallium-67 imaging
	Y94.x	Radiopharmaceutical imaging
	Y97.x	Radiology with contrast
	Y98.x	Radiology procedures
Exploration	M83.2	Exploration of retropubic space
	P27.8	Other specified exploration of vagina
	S57.8	Other specified exploration of other skin of other site
	T31.7	Exploration of groin NEC
	Y31.x	Exploration of organ NOC
	Y32.x	Re-exploration of organ NOC
Non-operations	E85.x-E98.x	Non operations on lower respiratory tract
	Y90.x	Other non-operations
	Y92.x	Support for preparation for radiotherapy
	Y99.x	Donor status
Methods of operation	Y02.x	Placement of prosthesis in organ NOC
	Y21.x	Cytology of organ NOC
	Y22.x	Drainage of organ NOC
	Y25.x	Suture of organ NOC
	Y41.x	Examination of organ NOC
Approach to organ	Y50.x	Approach through abdominal cavity
	Y52.x	Approach to organ through other opening
	Y53.x	Approach to organ under image control
	Y75.x	Minimal access to abdominal cavity
	Y76.x	Minimal access to other body cavity
Staged procedures	Y70.x	Early operations NOC
Anaesthetic	Y80.x	General anaesthetic
	Y81.x	Spinal anaesthetic
	Y82.x	Local anaesthetic
	Y84.x	Other anaesthetic

continued . . .

. . . continued

Category	*OPCS-4 codes*	*Description*
Sites of operation	O16.1	Pelvis NEC
	Z31.x	Other abdominal organ
	Z41.x	Upper urinary tract
	Z42.x	Lower urinary tract
	Z44.x	Vagina
	Z53.x	Abdominal wall
Therapeutic interventions	A52.x	Therapeutic epidural injection
	L91.2	Insertion of central venous catheter NEC
	L91.4	Removal of central venous catheter
	L99.7	Percutaneous transluminal peripheral insertion of central catheter
	M47.x	Urethral catheterisation of bladder
	M49.2	Change of suprapubic tube into bladder
	M49.3	Removal of suprapubic tube from bladder
	S42.1	Primary suture of skin NEC
	S42.2	Delayed primary suture of skin NEC
	S42.3	Secondary suture of skin NEC
	S42.8	Other specified suture of skin of other site
	S42.9	Unspecified suture of skin of other site
	S47.6	Incision of skin NEC
	S57.4	Dressing of skin NEC
	S57.5	Attention to dressing of skin NEC
	S57.6	Cleansing and sterilisation of skin NEC
	S57.7	Dressing of skin using vacuum assisted closure device NEC
	X29.2	Continuous intravenous infusion of therapeutic substance NEC
	X30.8	Other specified injection of therapeutic substance
	X30.9	Unspecified injection of therapeutic substance
	X31.2	Intravenous pyelography
	X56.9	Unspecified intubation of trachea

Supplementary Table S4: List of OPCS-4 procedure codes considered to be rescue procedures associated with complications caused by the index mesh insertion procedure.

OPCS-4 codes	*OPCS-4 description*
E42.3	Temporary tracheostomy
E42.7	Removal of tracheostomy tube
L70.3	Ligation of artery not elsewhere classified
M37.3	Repair of rupture of bladder
M37.8	Other specified other repair of bladder
M37.9	Unspecified other repair of bladder
M39.2	Open removal of foreign body from bladder
M42.2	Endoscopic cauterisation of lesion of bladder
M44.3	Endoscopic removal of foreign body from bladder
M44.4	Endoscopic removal of blood clot from bladder
M73.7	Repair of rupture of urethra not elsewhere classified
M73.8	Other specified repair of urethra
M73.9	Unspecified repair of urethra
P20.3	Cauterisation of lesion of vagina
P27.1	Evacuation of haematoma from vagina
P29.4	Removal of foreign body from vagina
S11.1	Cauterisation of lesion of skin not elsewhere classified
S42.4	Resuture of skin not elsewhere classified
S57.1	Debridement of skin not elsewhere classified
T30.1	Reopening of abdomen and re-exploration of intra-abdominal operation site and surgical arrest of postoperative bleeding
T30.2	Reopening of abdomen and re-exploration of intra-abdominal operation site not elsewhere classified
T30.3	Reopening of abdomen not elsewhere classified
T30.4	Opening of abdomen and exploration of groin
T30.8	Other specified opening of abdomen
T30.9	Unspecified opening of abdomen
X33.2	Intravenous blood transfusion of packed cells
X33.9	Unspecified other blood transfusion
X34.1	Transfusion of coagulation factor
X34.2	Transfusion of plasma not elsewhere classified
X35.5	Intravenous injection of antimicrobial therapy
X40.3	Haemodialysis not elsewhere classified
X40.4	Haemofiltration
X50.1	Direct current cardioversion
X50.2	External cardioversion not elsewhere classified
X50.3	Advanced cardiac pulmonary resuscitation
X50.9	Unspecified external resuscitation

Supplementary File S5: R code for data manipulation and analysis. Scripts and functions, written in the R programming language, are provided in the interests of reproducibility.

S5.1 Introduction

S5.1.1 R code

The code consists of a main script which executes a series of separate scripts (§S5.2) for each step in the analysis. Helper functions, used repeatedly during the analysis, are also provided (§S5.3). The scripts create a log file containing the main results and write interim data frames at each step. A complete analysis of approximately 500,000 HES episodes takes about 25 minutes to complete on a typical desktop PC.

S5.1.2 HES data

The HES records on which the code operates are available from NHS Digital, subject to their rules concerning requests for episode-level data. Only selected HES fields from the "inpatient universe" are used in this analysis - the non-NULL ones listed in `import.R`. Specifically, using HES terminology: FYEAR, PSEUDO_HESID, ADMIDATE, ADMIMETH, DIAG_4_CONCAT, DISDATE, DISDEST, DISMETH, EPIEND, EPISTART, OPERTN_4_CONCAT, PROCODE3, SEX, STARTAGE.

S5.2 Scripts for each step of analysis

S5.2.1 main.R

```
# --------------------------------------------------------------------------
# main.R
#
# Description:
# ============
# Runs all steps in mesh/sui analysis.
#
# History:
# ========
# 23.02.2015. A.J. Sims. Created.
# --------------------------------------------------------------------------

# clean up
rm(list=ls())

# send output to a file
logfile <- paste("log/", format(Sys.time(), "%Y%m%d%H%M%S"), ".log", sep='')
sink(file=logfile, append=F, split=T)
cat(format(Sys.time(), "%Y-%m-%d %H:%M:%S"), "\n")
rv <- R.Version()
cat(rv$version.string, "\n\n")

# create 'suipop.Rdata' from CSV dump of HES searches
source("import.R", local=T)

# create cohort data sets for SUI and POP indications
# as if separate HES searches had been done
source("cohorts.R", local=T)

# extract raw activity data from uncleaned data in 'sui.Rdata'
source("activity.R", local=T)

# clean 'sui.Rdata' and save to 'sui-cleaned.Rdata'
source("clean.R", local=T)
```

```
# identify index procedures
source("indexprocs.R", local=T)

# split cohort into confounded and unconfounded
source("split.R", local=T)

# peri-procedural complications
source("pericomp.R", local=T)

# analysis of 30 day readmissions
source("thirty.R")

# generate post-procedure event tables
source("postproc.R", local=T)

# survival analysis
source("history.R", local=T)

# overall safety analysis
source("safety.R", local=T)

# stop output
cat("\n")
cat(format(Sys.time(), "%Y-%m-%d %H:%M:%S"), "\n\n")
sink()
rm(list=ls())
```

S5.2.2 import.R

```
# --------------------------------------------------------------------------
# import.R
#
# Description:
# ============
# Reads raw HES search, filters fields and saves as data frame in an .Rdata
# file.
#
# History:
# ========
# 23.02.2015. A.J. Sims. Created.
# 12.03.2015. A.J. Sims. Added spell id, and separated DIAG and OPERTN into
#                        lists within the data frame.
# 31.03.2015. A.J. Sims. Episodes given unique labels (row names).
# 29.06.2015. A.J. Sims. Creates combined SUI+POP data frame.
# 10.08.2015. A.J. Sims. Updated regular expression.
# 07.12.2015. A.J. Sims. Minor changes to fields for 0708-1415 dataset.
# --------------------------------------------------------------------------

# clear workspace...
rm(list=ls())

cat('HES extract\n')
cat('-----------\n')

# source of episodes
where <- "sql"

# ----------------------------------------------------------------------------
# read from CSV
# ----------------------------------------------------------------------------
if (where == "csv") {
```

```
# which HES search?
path <- "S:/hes/kim/MESH"
file <- "20151126_RX029_MESH_0708_1415_M533M536M521_allep.csv"
cat(file, '\n\n')

# block size to read...
nrows <- -1

# define some classes and methods for colClasses in read...
setClass("HESDate")
setAs("character", "HESDate", function(from) as.Date(from, format="%d%b%Y"))

# define column classes, to permit processing on input...
colClasses=c(
  FYEAR="character",
  EPIKEY="NULL",
  PSEUDO_HESID="factor",
  ADMIDATE="HESDate",
  ADMIMETH="factor",
  DIAG_4_01="NULL",
  DIAG_4_02="NULL",
  DIAG_4_03="NULL",
  DIAG_4_04="NULL",
  DIAG_4_05="NULL",
  DIAG_4_06="NULL",
  DIAG_4_07="NULL",
  DIAG_4_08="NULL",
  DIAG_4_09="NULL",
  DIAG_4_10="NULL",
  DIAG_4_11="NULL",
  DIAG_4_12="NULL",
  DIAG_4_13="NULL",
  DIAG_4_14="NULL",
  DIAG_4_15="NULL",
  DIAG_4_16="NULL",
  DIAG_4_17="NULL",
  DIAG_4_18="NULL",
  DIAG_4_19="NULL",
  DIAG_4_20="NULL",
  DIAG_4_CONCAT="character",     # 0708-1415 only, not used in analysis
  DIAG_COUNT="NULL",
  DISDATE="HESDate",
  DISDEST="integer",
  DISMETH="integer",
  EPIDUR="NULL",
  EPIEND="HESDate",
  EPIORDER="NULL",
  EPISTART="HESDate",
  EPISTAT="NULL",                 # 0708-1415 only, not used in analysis
  EPITYPE="NULL",                 # 0708-1415 only, not used in analysis
  FCE="NULL",
  OPERTN_4_01="NULL",
  OPERTN_4_02="NULL",
  OPERTN_4_03="NULL",
  OPERTN_4_04="NULL",
  OPERTN_4_05="NULL",
  OPERTN_4_06="NULL",
  OPERTN_4_07="NULL",
  OPERTN_4_08="NULL",
  OPERTN_4_09="NULL",
  OPERTN_4_10="NULL",
  OPERTN_4_11="NULL",
```

```
    OPERTN_4_12="NULL",
    OPERTN_4_13="NULL",
    OPERTN_4_14="NULL",
    OPERTN_4_15="NULL",
    OPERTN_4_16="NULL",
    OPERTN_4_17="NULL",
    OPERTN_4_18="NULL",
    OPERTN_4_19="NULL",
    OPERTN_4_20="NULL",
    OPERTN_4_21="NULL",
    OPERTN_4_22="NULL",
    OPERTN_4_23="NULL",
    OPERTN_4_24="NULL",
    OPERTN_4_CONCAT="character",
    OPDATE_01="NULL",
    OPDATE_02="NULL",
    OPDATE_03="NULL",
    OPDATE_04="NULL",
    OPDATE_05="NULL",
    OPDATE_06="NULL",
    OPDATE_07="NULL",
    OPDATE_08="NULL",
    OPDATE_09="NULL",
    OPDATE_10="NULL",
    OPDATE_11="NULL",
    OPDATE_12="NULL",
    OPDATE_13="NULL",
    OPDATE_14="NULL",
    OPDATE_15="NULL",
    OPDATE_16="NULL",
    OPDATE_17="NULL",
    OPDATE_18="NULL",
    OPDATE_19="NULL",
    OPDATE_20="NULL",
    OPDATE_21="NULL",
    OPDATE_22="NULL",
    OPDATE_23="NULL",
    OPDATE_24="NULL",
    OPERTN_COUNT="NULL",  # 0708-1415 only, not used in analysis
    PROCODE3="factor",
    #ORG_DESCRIPTION="NULL",  # 0708-1314 only, not used in analysis
    SEX="integer",
    SPELBGIN="NULL",
    SPELDUR="NULL",
    SPELDUR_CALC="NULL",
    SPELEND="NULL",
    STARTAGE="integer",
    STARTAGE_CALC="NULL",
    SUSSPELLID="NULL"
  )

  # read and convert...
  SP <- read.csv(file=paste(path, file, sep='/'),
                 header=TRUE,
                 row.names=NULL,
                 na.strings=c("NA", "01JAN1800", "01JAN1801"),
                 strip.white=T,
                 colClasses=colClasses,
                 nrows=nrows)

  # drop first data row for 14/15 data set
  SP <- SP[-c(1),]
}
```

```r
# ----------------------------------------------------------------------------
# read from SQL
# ----------------------------------------------------------------------------
if (where=="sql") {

  # load RODBC library
  library("RODBC")

  # data source and table name
  dsn <- "HES Archive"
  table.name  = c("20151126_RX029_MESH_0708_1415_M533M536M521_allep")
  cat("SQL table:", table.name, '\n')

  # establish database connection
  con <- odbcConnect(dsn,
                   interpretDot=FALSE,
                   case="nochange"
  )

  # find size of results set
  df.temp <- sqlQuery(channel=con,
                    query=paste("SELECT COUNT(*) AS NROW FROM", table.name, sep=' '),
                    buffsize=1,
                    rows_at_time=1,
                    max=1)
  buffsize <- as.integer(df.temp$NROW[1])
  cat(buffsize, "rows in table", table.name, '\n')

  # NA strings
  na.strings <- c("NA", '', "0000-00-00", "1800-01-01", "1801-01-01")

  # query into a data frame
  qry <- paste("SELECT",
               "FYEAR,",
               "PSEUDO_HESID,",
               "STR_TO_DATE(ADMIDATE, '%d%b%Y') AS ADMIDATE,",
               "ADMIMETH,",
               "DIAG_4_CONCAT,",
               "STR_TO_DATE(DISDATE, '%d%b%Y') AS DISDATE,",
               "DISDEST,",
               "DISMETH,",
               "STR_TO_DATE(EPIEND, '%d%b%Y') AS EPIEND,",
               "STR_TO_DATE(EPISTART, '%d%b%Y') AS EPISTART,",
               "OPERTN_4_CONCAT,",
               "PROCODE3,",
               "SEX,",
               "STARTAGE",
            "FROM",
               table.name,
             sep=' '
  )
  SP <- sqlQuery(channel=con,
               query=qry,
               as.is=c(1,5,11),  # fields to leave as character
               rows_at_time=1,
               na.strings=na.strings
               )

  # close database connection
  odbcCloseAll()

  # drop first data row for 14/15 data set
```

```r
  SP <- SP[-c(1),]
  cat("\n")
}

# Add a column which defines a unique episode ID. This is useful in
# later analysis to keep track of individual episodes.
SP$EPI_ID <- paste('E', rownames(SP), sep='')

# convert FYEAR to factor (blank = NA)
SP$FYEAR[SP$FYEAR==''] <- NA
SP$FYEAR <- as.factor(SP$FYEAR)

# clean (as per HES data dictionary)...
SP$STARTAGE[SP$STARTAGE>7000] <- 0    # babies < 1 year

# convert procedures and diagnoses to list...
SP$OPERTN <- strsplit(SP$OPERTN_4_CONCAT, ',')
SP$OPERTN_4_CONCAT <- NULL

SP$DIAG <- strsplit(SP$DIAG_4_CONCAT, ',')
SP$DIAG_4_CONCAT <- NULL

# spells...
SP$SPELL <- as.integer(as.factor(
  paste(SP$PSEUDO_HESID,
        SP$ADMIDATE,
        SP$ADMIMETH,
        SP$PROCODE3,
        sep='+')
))

# save it...
save(file="data/suipop.Rdata", SP)

# raw data summary...
cat("Raw data\n--------\n")
cat(nrow(SP), "episodes\n")
PATIENTS <- unique(SP$PSEUDO_HESID)
cat(length(PATIENTS), "patients\n")

# basic checks...
cat("\n")
cat("Checks on data:\n--------------\n")
cat("ADMIDATE range: ", as.character(min(SP$ADMIDATE, na.rm=TRUE)),
    as.character(max(SP$ADMIDATE, na.rm=TRUE)), '\n')
cat("DISDATE range: ", as.character(min(SP$DISDATE, na.rm=TRUE)),
    as.character(max(SP$DISDATE, na.rm=TRUE)), '\n')
cat("EPIEND range: ", as.character(min(SP$EPIEND, na.rm=TRUE)),
    as.character(max(SP$EPIEND, na.rm=TRUE)), '\n')
cat("EPISTART range: ", as.character(min(SP$EPISTART, na.rm=TRUE)),
    as.character(max(SP$EPISTART, na.rm=TRUE)), '\n')

cat("FYEAR values: ")
print(unique(SP$FYEAR))

cat("ADMIMETH values:")
print(unique(SP$ADMIMETH))

cat("DISDEST values:")
print(unique(SP$DISDEST))

cat("DISMETH values:")
print(unique(SP$DISMETH))
```

```
cat("SEX values:")
print(unique(SP$SEX))

cat("STARTAGE range:", min(SP$STARTAGE, na.rm=TRUE), max(SP$STARTAGE, na.rm=TRUE), '\n')

# bad OPERTN or DIAG codes
pattern <- "^[[:upper:]][[:digit:]]{2}[X[:digit:]]{1}$"     # e.g. A001, P232, I10X

all.OPERTN <- unlist(SP$OPERTN)
all.OPERTN <- all.OPERTN[!is.na(all.OPERTN)]
bad.OPERTN <- grep(pattern, all.OPERTN, invert=T)
cat(length(bad.OPERTN), "bad OPERTN codes out of", length(SP$OPERTN), "episodes\n")
ct  <- as.data.frame(table(all.OPERTN[bad.OPERTN]))
if (nrow(ct)>0) {
  names(ct) <- c('code', 'freq')
  print(ct, row.names=F)
}

all.DIAG <- unlist(SP$DIAG)
all.DIAG <- all.DIAG[!is.na(all.DIAG)]
bad.DIAG <- grep(pattern, all.DIAG, invert=T)
cat(length(bad.DIAG), "bad DIAG codes out of", length(SP$DIAG), "episodes\n")
ct <- as.data.frame(table(unlist(SP$DIAG)[bad.DIAG]))
if (nrow(ct)>0) {
  names(ct) <- c('code', 'freq')
  print(ct, row.names=F)
}

cat("Bad ICD-10 codes:\n")
ct <- as.data.frame(table(all.DIAG[bad.DIAG]))
if (nrow(ct)>0) {
  names(ct) <- c('code', 'freq')
  print(ct, row.names=F)
}
cat('\n\n')

rm(list=ls())
```

S5.2.3 cohorts.R

```
# ------------------------------------------------------------------------------
# cohorts.R
#
# Description:
# ============
# Creates cohorts based on mesh/tape from suipop.Rdata.
#
# History:
# ========
# 24.02.2015. A.J. Sims. Created.
# 29.06.2015. A.J. Sims. Creates separate episode sets, as if separate HES
#                        searches had been run for each cohort.
# 18.08.2015. A.J. Sims. Uses revised epimatch.
# ------------------------------------------------------------------------------

# clear workspace...
rm(list=ls())

# load functions...
source("R/epimatch.R")
```

```
# load data set...
load('data/suipop.Rdata')

# ----------------------------------------------------------------------------
# SUI cohort
# ----------------------------------------------------------------------------
cat("SUI cohort\n")
cat("----------\n")

# find all episodes with M533, M536, M521
op.codes <- quote(M533 | M536 | M521)
ep.SUI <- with(SP, {
  sapply(OPERTN, epimatch, e.codes=op.codes)
})
cat(sum(ep.SUI), "episodes containing", deparse(op.codes), "\n")

# find patients having these episodes
cohort.SUI <- unique(SP$PSEUDO_HESID[ep.SUI])
cat(length(cohort.SUI), "patients\n")

# create a subset for the SUI cohort
SUI <- subset(SP, SP$PSEUDO_HESID %in% cohort.SUI, drop=T)
cat(nrow(SUI), "episodes for these patients\n")

save(SUI, file="data/sui.Rdata")
cat("\n")

# ----------------------------------------------------------------------------
# POP cohort
# ----------------------------------------------------------------------------
cat("POP cohort\n")
cat("----------\n")

# find all episodes with POP procedures
codes <- quote(P236 | P237 | P242 | P245 | P246 | Q544 | Q545 | Q546)
ep.POP <- with(SP, {
  sapply(OPERTN, epimatch, e.codes=codes)
})
cat(sum(ep.POP), "episodes containing", deparse(codes), "\n")

# find patients having these episodes
cohort.POP <- unique(SP$PSEUDO_HESID[ep.POP])
cat(length(cohort.POP), "patients\n")

# create a subset for the SUI cohort
POP <- subset(SP, SP$PSEUDO_HESID %in% cohort.POP, drop=T)
cat(nrow(POP), "episodes for these patients\n")
save(POP, file="data/pop.Rdata")
cat("\n")

# -----------------------------------------------------------------------------
# clear
# -----------------------------------------------------------------------------
rm(list=ls())
```

S5.2.4 activity.R

```
# ------------------------------------------------------------------------------
# activity.R
#
# Description:
```

```
# ============
# Computes raw activity to compare with HES on-line.
#
# History:
# ========
# 24.02.2015. A.J. Sims. Created.
# 29.06.2015. A.J. Sims. SUI only.
# 18.08.2015. A.J. Sims. Uses updated epimatch.
# ---------------------------------------------------------------------------

# clear workspace...
rm(list=ls())

# load functions...
source("R/epimatch.R")

# load data set...
load('data/sui.Rdata')

# --------------------------------------------------------------------------
# Function to calculate raw activity for specified code combination
# --------------------------------------------------------------------------
activity <- function(codes) {
  # find all episodes that include specified codes...
  activity <- with(SUI, sapply(OPERTN, FUN=epimatch, e.codes=codes))
  cat(length(which(activity)), "episodes with", deparse(codes), "\n")
  ct <- ftable(SUI$FYEAR[activity])
  fy <- unlist(attr(ct, "col.vars"))
  for (i in 1:length(ct)) {
    cat(format(ct[i], width=6, justify="right"), "in", fy[i], "\n")
  }
}

# --------------------------------------------------------------------------
# raw data summary...
# --------------------------------------------------------------------------
cat("Raw data\n--------\n")
cat(nrow(SUI), "episodes\n")
PATIENTS <- unique(SUI$PSEUDO_HESID)
cat(length(PATIENTS), "patients\n")

# --------------------------------------------------------------------------
# raw activity (to match with HSCIC)
# --------------------------------------------------------------------------
cat("\n")
cat("Activity - incontinence\n")
cat("-----------------------\n")
activity(quote(M533))
activity(quote(M536))
activity(quote(M521))
activity(quote(M534))
activity(quote(M535))
activity(quote(M533 & (Y037 | Y264)))
activity(quote(M537))
activity(quote(M536 & (Y037 | Y264)))
```

S5.2.5 clean.R

```
# ---------------------------------------------------------------------------
# clean.R
#
```

```
# Description:
# ============
# Cleans mesh.Rdata and saves as mesh-cleaned.Rdata
#
# History:
# ========
# 24.02.2015. A.J. Sims. Created.
# 28.04.2015. A.J. Sims. Moved to separate script.
# 30.07.2015. A.J. Sims. Uses EPI_ID instead of row.names for episoide IDs.
# 18.08.2015. A.J. Sims. Uses updated epimatch.
# 11.09.2015. A.J. Sims. Clearer definition of index procedure.
# 28.09.2015. A.J. Sims. DISDATE added to index ordering to break ties.
# 07.12.2015. A.J. Sims. Added main code M528, M538, M537 as main codes which
#                         qualify as index procedures.
# 07.12.2015. A.J. Sims. Drop patients with records AFTER date of death.
# 08.12.2015. A.J. Sims. Drop episodes with missing FYEAR.
# 27.01.2016. A.J. Sims. OPERTN and DIAG with empty list (e.g. '-' only) are
#                         converted to NA.
# 14.06.2016. A.J. Sims. Codes M528 and M538 must be followed by a Y code to
#                         qualify as a mesh code.
# ------------------------------------------------------------------------------

rm(list=ls())
load(file="data/sui.Rdata")
source("R/epimatch.R")
source("R/opcssplit.R")
source("R/opcs_has_supp.R")
source("interventions.R")

# -----------------------------------------------------------------------------
# function to drop all episodes for specified patients
# -----------------------------------------------------------------------------
drop.patients <- function(HESIDS) {

  cat(length(HESIDS), "patients\n")

  # find all episodes for specified patients
  to.drop <- which(SUI$PSEUDO_HESID %in% HESIDS)
  cat(length(to.drop), "episodes for these patients to be dropped\n")

  # remove dropped episodes from analysis
  if (length(to.drop) > 0) {
    SUI <<- SUI[-(to.drop),]
    cat(nrow(SUI), "remaining episodes\n")
    PATIENTS <- unique(SUI$PSEUDO_HESID)
    cat(length(PATIENTS), "remaining patients\n")
  }
}

# -----------------------------------------------------------------------------
# function to identify index episodes
# -----------------------------------------------------------------------------
find.index <- function() {

  # episodes with at least one tape/mesh intervention
  cat("\nFind index episodes\n-------------------\n")
  SUI.mt <- sapply(SUI$OPERTN, FUN=int.anymesh)
  cat(length(which(SUI.mt)), "episodes with at least 1 main tape/mesh code in OPERTN\n")

  # index episodes with at least one main code
  keep <- c('PSEUDO_HESID', 'ADMIDATE', 'DISDATE', 'EPI_ID')
  ss <- SUI[SUI.mt, keep, drop=T]
  ss <- with(ss, ss[order(ADMIDATE, DISDATE),])
```

```r
  ss <- ss[!duplicated(ss$PSEUDO_HESID),]
  ndx <- ss$EPI_ID # episode IDs of index procedures
  rm(ss)
  rm(SUI.mt)
  cat(length(ndx), "index episodes\n")
  PATIENTS <- unique(SUI$PSEUDO_HESID)
  cat(length(PATIENTS)-length(ndx), "patients with no index\n")

  return(ndx)
}

# ---------------------------------------------------------------------------
# remove duplicated rows
# ---------------------------------------------------------------------------
cat("\n")
cat("Duplicated episodes\n")
cat("-------------------\n")
dup <- duplicated(SUI[,!(names(SUI) %in% c('EPI_ID'))])
#SUI <- unique(SUI)
SUI <- SUI[!dup,]
cat(sum(dup), "duplicated episodes removed\n")
cat(nrow(SUI), "episodes remaining\n")

# ---------------------------------------------------------------------------
# drop rows with missing data in essential fields...
# ---------------------------------------------------------------------------
cat("\n")
cat("Missing data (all episodes)\n")
cat("---------------------------\n")

EXPAT <- c()
with(SUI, {

  to.drop <- c()

  drop <- which(is.na(PSEUDO_HESID))
  cat(length(drop), "episodes with missing PSEUDO_HESID\n")
  to.drop <- c(to.drop, drop)

  drop <- which(is.na(SEX))
  cat(length(drop), "episodes with missing SEX\n")
  to.drop <- c(to.drop, drop)

  drop <- which(is.na(ADMIDATE))
  cat(length(drop), "episodes with missing ADMIDATE\n")
  to.drop <- c(to.drop, drop)

  drop <- which(is.na(ADMIMETH))
  cat(length(drop), "episodes with missing ADMIMETH\n")
  to.drop <- c(to.drop, drop)

  drop <- which(is.na(PROCODE3))
  cat(length(drop), "episodes with missing PROCODE3\n")
  to.drop <- c(to.drop, drop)

  drop <- which(is.na(FYEAR))
  cat(length(drop), "episodes with missing FYEAR\n")
  to.drop <- c(to.drop, drop)

  to.drop <- unique(to.drop)
  cat(length(to.drop), "unique episodes with missing essential fields\n")

  # find all patients that have some missing essential data
```

```
  EXPAT <<- PSEUDO_HESID[to.drop]

  rm(drop)
  rm(to.drop)
})
drop.patients(EXPAT)
rm(EXPAT)

# ----------------------------------------------------------------------------
# inclusion criteria applying to all episodes for each patient
# ----------------------------------------------------------------------------

cat("\n")
cat("Inclusion criteria (all episodes)\n")
cat("---------------------------------\n")

EXPAT <- c()
with(SUI, {

  to.drop <- c()

  drop <- which(!(SEX==2))
  cat(length(drop), "episodes with SEX != 2\n")
  to.drop <- c(to.drop, drop)

  to.drop <- unique(to.drop)
  cat(length(to.drop), "unique episodes not matching inclusion criteria\n")

  # find all patients involved
  EXPAT <<- PSEUDO_HESID[to.drop]

  rm(drop)
  rm(to.drop)

})
drop.patients(EXPAT)
rm(EXPAT)

# ---------------------------------------------------------------------------
# missing data in index episodes
# ---------------------------------------------------------------------------
ndx <- find.index()
cat("\n")

cat("Missing data (index episodes)\n")
cat("-----------------------------\n")

EXPAT <- c()
with(SUI[SUI$EPI_ID %in% ndx,], {

  to.drop <- c()

  drop <- which(is.na(STARTAGE))
  cat(length(drop), "index episodes with missing STARTAGE\n")
  to.drop <- c(to.drop, drop)

  to.drop <- unique(to.drop)
  cat(length(to.drop), "unique index episodes with missing essential data\n")

  # all patients with missing fields in index episode
  EXPAT <<- PSEUDO_HESID[to.drop]

})
```

```r
drop.patients(EXPAT)
rm(EXPAT)

# ---------------------------------------------------------------------------
# inclusion criteria for index episodes
# ---------------------------------------------------------------------------
ndx <- find.index()
cat("\n")
cat("Inclusion criteria (index episodes)\n")
cat("-----------------------------------\n")

EXPAT <- c()
with(SUI[SUI$EPI_ID %in% ndx,], {

  to.drop <- c()

  drop <- which(STARTAGE < 18)
  cat(length(drop), "index episodes with STARTAGE < 18\n")
  to.drop <- c(to.drop, drop)

  adm.inc <- c('11', '12', '13', '21', '22', '23', '24', '25', '2A', '2B', '2D', '28', '81')
  drop <- which(!(ADMIMETH %in% adm.inc))
  cat(length(drop), "index episodes with disallowed ADMIMETH\n")
  to.drop <- c(to.drop, drop)

  to.drop <- unique(to.drop)
  cat(length(to.drop), "unique index episodes not matching inclusion criteria\n")

  # all patients with invalid fields in index episode
  EXPAT <<- PSEUDO_HESID[to.drop]

})
drop.patients(EXPAT)
rm(EXPAT)

# ----------------------------------------------------------------------------
# exclude patients with episodes after they have been marked as died in hospital
# ----------------------------------------------------------------------------

cat("\n")
cat("Patients with episodes AFTER date of death\n")
cat("------------------------------------------\n")

# episodes
event.death <- SUI$DISMETH==4
deaths <- SUI[event.death, c("PSEUDO_HESID", "SPELL", "ADMIDATE", "DISDATE")]
cat(nrow(deaths), "in-hospital death episodes\n")

# spells
deaths <- deaths[order(deaths$PSEUDO_HESID, deaths$ADMIDATE, deaths$SPELL),]
dup <- duplicated(deaths$PSEUDO_HESID)
deaths <- deaths[!dup,]
cat(nrow(deaths), "in-hospital death spells\n")

# find any patients with records AFTER date of death
revivs <- apply(deaths, 1, FUN=function(row) {
  person <- row['PSEUDO_HESID']
  dod <- row['DISDATE']
  ss <- (SUI$PSEUDO_HESID==person & SUI$ADMIDATE>dod)
  return(sum(ss)>0)
})
cat(sum(revivs, na.rm=T), "patients with episodes AFTER date of death\n")
print(deaths[revivs, c("SPELL", "ADMIDATE", "DISDATE")], row.names=F)
```

```
drop.patients(deaths$PSEUDO_HESID[revivs])

# ---------------------------------------------------------------------------
# final identification of index procedures
# ---------------------------------------------------------------------------
ndx <- find.index()
cat("\n")

# ---------------------------------------------------------------------------
# add index field
# ---------------------------------------------------------------------------
SUI$Index <- F
SUI[SUI$EPI_ID %in% ndx,'Index'] <- T

# ---------------------------------------------------------------------------
# remove non-OPCS codes from OPERTN
# ---------------------------------------------------------------------------
cat("Bad OPCS and ICD-10 codes\n")
cat("-------------------------\n")

"%w/o%" <- function(x, y) x[!x %in% y] #--  x without y
SUI$OPERTN <- sapply(SUI$OPERTN, function(codes) {
  # eliminate non-OPCS codes
  opcodes <- codes %w/o% c("&", "-", "-   ", "&   ")
  if (length(opcodes)==0) {
    return(NA)
  } else {
    return(opcodes)
  }
})
pattern <- "^[[:upper:]][[:digit:]]{2}[X[:digit:]]{1}$"     # e.g. A001, P232, I10X
all.OPERTN <- unlist(SUI$OPERTN)
all.OPERTN <- all.OPERTN[!is.na(all.OPERTN)]
bad.OPERTN <- grep(pattern, all.OPERTN, invert=T)
cat(length(bad.OPERTN), "bad OPERTN codes out of", length(SUI$OPERTN), "episodes\n")
ct <- as.data.frame(table(all.OPERTN[bad.OPERTN]))
if (nrow(ct)>0) {
  names(ct) <- c('code', 'freq')
  print(ct, row.names=F)
}

# ---------------------------------------------------------------------------
# remove non-ICD-10 codes from DIAG
# ---------------------------------------------------------------------------
SUI$DIAG <- sapply(SUI$DIAG, function(codes) {
  # eliminate non-ICD-10 codes
  opcodes <- codes %w/o% c("&", "-", "-   ", "&  ")
  if (length(opcodes)==0) {
    return(NA)
  } else {
    return(opcodes)
  }
})
all.DIAG <- unlist(SUI$DIAG)
all.DIAG <- all.DIAG[!is.na(all.DIAG)]
bad.DIAG <- grep(pattern, all.DIAG, invert=T)
cat(length(bad.DIAG), "bad DIAG codes out of", length(SUI$DIAG), "episodes\n")
ct <- as.data.frame(table(all.DIAG[bad.DIAG]))
if (nrow(ct)>0) {
  names(ct) <- c('code', 'freq')
  print(ct, row.names=F)
}
cat("\n")
```

```
# ----------------------------------------------------------------------------
# save cleaned data set
# ----------------------------------------------------------------------------
save(SUI, file="data/sui-cleaned.Rdata")
rm(list=ls())
```

S5.2.6 indexprocs.R

```
# -----------------------------------------------------------------------------
# indexprocs.R
#
# Description:
# ============
# Identifies index procedures and collects further details about them.
#
# History:
# ========
# 14.12.2015. A.J. Sims. Created from parts of pericomp.R.
# 23.12.2015. A.J. Sims. Repair and removal codes added to white list (because
#                        they count as index procedures, which are excluded
#                        later). Also added POP codes to white list, for which
#                        same argument applies.
# 10.05.2016. A.J. Sims. Step to identify complications in index procedures
#                        moved before splitting cohort.
# 14.06.2016. A.J. Sims. Use functions in interventions.R.
# 21.06.2016. A.J. Sims. LoS set to NA for DISDATE < ADMIDATE; but whole
#                        spell not excluded.
# 22.02.2017. A.J. Sims. Edits to activity graph for clarity.
# -----------------------------------------------------------------------------

rm(list=ls())
source("R/epimatch.R")
source("R/inChapters.R")
source("R/inSections.R")
source("R/opcssplit.R")
source("R/opcs_has_supp.R")
source("R/icdsplit.R")
source("R/is_complication.R")
source("pubfig.R")
source("interventions.R")

load("data/sui-cleaned.Rdata")

# ----------------------------------------------------------------------------
# Build dataframe of episodes in index spells
# ----------------------------------------------------------------------------
cat("SUI index spells\n----------------\n")
IEP.SUI <- merge(data.frame(SPELL=SUI$SPELL[SUI$Index==T]), SUI, by="SPELL", sort=T)

cat(nrow(IEP.SUI), "episodes in index spells\n")
cat(sum(IEP.SUI$Index==T), "index episodes\n")
cat(sum(IEP.SUI$Index==F), "non-index episodes\n")

rm(SUI)

# ------------------------------------------------------
# collect information about index spells
# ------------------------------------------------------

cat("\n")
cat("Information about index spells\n")
```

```r
cat("------------------------------\n")

# count of episodes per spell
SP.SUI <- with(IEP.SUI, {aggregate(EPI_ID, by=list(SPELL), FUN=length)})
names(SP.SUI) <- c("SPELL", "Count")

# diagnostic print
cat(nrow(SP.SUI), "index spells\n")
cat(sum(SP.SUI$Count>1), "index spells with >1 episode\n")

# require diagnosis of SUI in index spell
sui.codes <- quote(N393 | N394 | R32X | T831 | T834 | T835 | T836 | T838 | T839 | Z466 )
IEP.SUI$isSUI <- sapply(IEP.SUI$DIAG, FUN=epimatch, e.codes=sui.codes)
df <- with(IEP.SUI, {aggregate(isSUI, by=list(SPELL), FUN=any)})
names(df) <- c("SPELL", "isSUI")
SP.SUI <- merge(SP.SUI, df, by="SPELL", sort=T)
rm(df)
IEP.isSUI <- NULL
cat(sum(SP.SUI$isSUI), "index spells with SUI diagnosis\n")
cat(sum(SP.SUI$isSUI==F), "index spells without SUI diagnosis\n")
SP.SUI <- SP.SUI[SP.SUI$isSUI==T, , drop=T]

# add extra columns to spell frame
keep <- c("SPELL", "PSEUDO_HESID", "PROCODE3", "STARTAGE", "ADMIMETH", "ADMIDATE",
          "DISMETH", "DISDATE", "FYEAR")
demog.df <- IEP.SUI[,keep]
dup <- which(duplicated(demog.df[,c("SPELL", "PROCODE3", "ADMIMETH")]))
if (length(dup)>0) {
  demog.df <- demog.df[-dup,]
}
cat(nrow(demog.df), "index spells with demographic details found\n")
SP.SUI <- merge(SP.SUI, demog.df, by="SPELL", sort=T)
rm(demog.df)

SP.SUI$LoS <- SP.SUI$DISDATE - SP.SUI$ADMIDATE
cat(sum(SP.SUI$LoS<0, na.rm=T), "spells with LoS < 0 days; LoS set to NA\n")
SP.SUI$LoS[SP.SUI$LoS < 0] <- NA

cat(nrow(SP.SUI), "spells with SUI diagnosis and demographic details\n")


# -----------------------------------------------------
# classify index spells by type of mesh operation
# -----------------------------------------------------

cat("\n")
cat("Classify index spells by type of mesh operation\n")
cat("-----------------------------------------------\n")

# removals
IEP.SUI$removal <- sapply(IEP.SUI$OPERTN, FUN=int.removal)
cat(sum(IEP.SUI$removal), "index episodes with removal of mesh\n")

# repairs
IEP.SUI$repair <- sapply(IEP.SUI$OPERTN, FUN=int.repair)
cat(sum(IEP.SUI$repair), "index episodes with repair of mesh\n")

# renewals
IEP.SUI$renewal <- sapply(IEP.SUI$OPERTN, FUN=int.renewal)
cat(sum(IEP.SUI$renewal), "index episodes with renewal of mesh\n")

# POP operations (by main code)
IEP.SUI$pop <- sapply(IEP.SUI$OPERTN, FUN=int.pop)
```

```r
cat(sum(IEP.SUI$pop), "index episodes involving POP surgery\n")

# combine all these exclusions
IEP.SUI$rrrrp <- with(IEP.SUI, removal | repair | renewal | pop)
cat(sum(IEP.SUI$rrrrp), "index episodes with mesh removal/repair/renewal or pop surgery\n")

# exclude spells with r/r/r or pop (which are not index spells)
df <- with(IEP.SUI, {aggregate(rrrrp, by=list(SPELL), FUN=any)})
names(df) <- c("SPELL", "rrrrp")
SP.SUI <- merge(SP.SUI, df, by="SPELL", sort=T)
rm(df)
IEP.SUI$rrrrp <- NULL
cat(sum(SP.SUI$rrrrp), "'index' spells with repair/removal/renewal or POP surgery - excluded\n")
SP.SUI <- SP.SUI[SP.SUI$rrrrp==F,, drop=T]
cat(nrow(SP.SUI), 'remaining index spells (')
cat(length(unique(SP.SUI$PSEUDO_HESID)), "patients)\n")
cat("\n")

# tot, tvt and minisling codes
tot.codes <- quote(M536)
IEP.SUI$TOT <- sapply(IEP.SUI$OPERTN, FUN=epimatch, e.codes=tot.codes)
cat(sum(IEP.SUI$TOT), "index episodes including TOT main code\n")

tvt.codes <- quote(M533)
IEP.SUI$TVT <- sapply(IEP.SUI$OPERTN, FUN=epimatch, e.codes=tvt.codes)
cat(sum(IEP.SUI$TVT), "index episodes including TVT main code\n")

ms.codes <- quote(M521)
IEP.SUI$MS <- sapply(IEP.SUI$OPERTN, FUN=epimatch, e.codes=ms.codes)
cat(sum(IEP.SUI$MS), "index episodes including MS main code\n")

# combine into spells (for eligible spells only)
cat("Check for combinations of TVT, TOT, MS:\n")
df <- aggregate(IEP.SUI[,c('TOT', 'TVT', 'MS')], by=list(IEP.SUI$SPELL), FUN=any)
names(df) <- c('SPELL', 'TOT', 'TVT', 'MS')
df$comb <- df$TOT + df$TVT + df$MS
SP.SUI <- merge(SP.SUI, df, by="SPELL", sort=T)
rm(df)
cat('  ', sum(SP.SUI$comb==0), "spells with no TVT, TOT or MS\n")
cat('  ', sum(SP.SUI$comb==1), "spells with one of TVT, TOT or MS\n")
cat('  ', sum(SP.SUI$comb>1), "spells with more than one TVT, TOT or MS\n")
SP.SUI$comb <- SP.SUI$comb>1
SP.SUI$TVT[SP.SUI$comb==T] <- F
SP.SUI$TOT[SP.SUI$comb==T] <- F
SP.SUI$MS[SP.SUI$comb==T] <- F

# anything else
combos <- IEP.SUI[IEP.SUI$SPELL %in% SP.SUI$SPELL[SP.SUI$comb==T],]
save(combos, file="data/combos.Rdata")
cat('  ', nrow(SP.SUI[SP.SUI$TOT==T & SP.SUI$comb==F,]), "index spells with TOT only\n")
cat('  ', nrow(SP.SUI[SP.SUI$TVT==T & SP.SUI$comb==F,]), "index spells with TVT only\n")
cat('  ', nrow(SP.SUI[SP.SUI$MS==T & SP.SUI$comb==F,]), "index spells with MS only\n")
cat('  ', nrow(SP.SUI[SP.SUI$comb==T,]), "index spells with combination\n")

# mark episodes in eligible spells
IEP.SUI$eligible <- IEP.SUI$SPELL %in% SP.SUI$SPELL
cat("\n")
cat(sum(IEP.SUI$eligible), "episodes in eligible spells marked\n")

# ------------------------------------------
# identify spells with complication
# ------------------------------------------
```

```r
cat("\n")
cat("Identify spells with complication\n")
cat("---------------------------------\n")

# data frame to store which code identified a chunk as a complication
# (so will have one entry per qualifying chunk). Also stores spell
# ID with which the chunk is associated, so can be referenced to
# type of tape introduction
which.comp <- data.frame(SPELL=NA, PSEUDO_HESID=NA, MainCode=NA, CompCode=NA, SuppCode=NA)

# identify complications from diagnostic codes, one episode per row
is.comp <- function(row) {
  chunks <- icdsplit(unlist(row['DIAG'], use.names=F))  # break into sequences
  chunks <- chunks[-1]                                  # ignore the first
  if (length(chunks) == 0) {
    return(F)
  }
  chunk.MT <- sapply(chunks, FUN=is.complication) # main and trigger codes, if any
  chunk.MT <- as.data.frame(t(chunk.MT))          # in columns
  chunk.MT <- chunk.MT[!is.na(chunk.MT$MainCode) | !is.na(chunk.MT$CompCode),]
  if (nrow(chunk.MT)>0) {
    chunk.MT <- cbind(SPELL=row['SPELL'], PSEUDO_HESID=row['PSEUDO_HESID'], chunk.MT)
    which.comp <<- rbind(which.comp, chunk.MT)
  }
  return(nrow(chunk.MT)>0)
}

# find spells with complication
keep <- c('PSEUDO_HESID', 'SPELL', 'EPI_ID', 'OPERTN', 'DIAG')
ss <- IEP.SUI[IEP.SUI$SPELL %in% SP.SUI$SPELL, keep]
cat(nrow(ss), 'index episodes in eligible spells\n')
ss$hasComplication <- apply(ss, 1, FUN=is.comp)
cat(sum(ss$hasComplication), "index episodes in eligible spells with complication\n")
bad.spells <- unique(ss$SPELL[ss$hasComplication])
cat(length(bad.spells), 'eligible index spells with complication\n')

df <- with(ss, {aggregate(hasComplication, by=list(SPELL), FUN=any)})
names(df) <- c("SPELL", "hasComplication")
SP.SUI <- merge(SP.SUI, df, by="SPELL", all.x=T, sort=T)
rm(df)

# tidy up which.comp
narow <- apply(which.comp, 1, function(x) all(is.na(x)))
which.comp <- which.comp[!narow, ]

# function to write codes in normalised order (i.e. one code per row)
normalise.codes <- function(chunk) {
  return(c(MainCode=chunk[1], SuppCode=paste(chunk[-1], collapse=' ')))
}

# collate procedures and diagnoses in spells with complication for
# further analysis
normalise <- function(row) {
  chunks <- opcssplit(unlist(row['OPERTN'], use.names=F))
  ops <- sapply(X=chunks, FUN=normalise.codes)
  ops <- as.data.frame(t(ops), optional=T)
  ops <- cbind(SPELL=unlist(row['SPELL']), EPI_ID=unlist(row['EPI_ID']),
               OorD='OPCS4', ops, row.names=NULL)
  chunks <- icdsplit(unlist(row['DIAG'], use.names=F))
  dgs <- sapply(X=chunks, FUN=normalise.codes)
  dgs <- as.data.frame(t(dgs), optional=T)
  dgs <- cbind(SPELL=unlist(row['SPELL']), EPI_ID=unlist(row['EPI_ID']),
               OorD='ICD10', dgs, row.names=NULL)
```

```r
  return(rbind(ops, dgs))
}

# write index procedures with complication in normalised order
spells.comp <- apply(ss[ss$SPELL %in% bad.spells,], 1, FUN=normalise)
spells.comp <- as.data.frame(do.call(rbind, spells.comp))
spells.comp$OorD <- as.factor(spells.comp$OorD)
spells.comp$MainCode <- as.character(spells.comp$MainCode)
spells.comp$SuppCode <- as.character(spells.comp$SuppCode)
spells.comp <- spells.comp[order(spells.comp$SPELL, spells.comp$EPI_ID),]

cat("spells with complication written in code normalised order to spells.comp:\n")
cat('  ', nrow(spells.comp), "code chunks\n")
cat('  ', length(unique(spells.comp$SPELL)), "unique spells\n")
cat('  ', length(unique(spells.comp$EPI_ID)), "unique episodes\n")

rm(ss)

# -------------------------------------------------------------
# Look for 'late' (other) operation codes, suggesting that
# procedures may have been done as rescue
# -------------------------------------------------------------
cat("\n")
cat("Index spells with 'late' operations\n")
cat("-----------------------------------\n")

# look for presence of late codes in index spells
late.codes <- quote(Y712 | Y713 | Y716 | Y717)
IEP.SUI$isLate <- sapply(IEP.SUI$OPERTN, FUN=epimatch, e.codes=late.codes)
df <- with(IEP.SUI, {aggregate(isLate, by=list(SPELL), FUN=any)})
names(df) <- c("SPELL", "isLate")
SP.SUI <- merge(SP.SUI, df, by="SPELL", sort=T)
rm(df)
IEP.SUI$isLate <- NULL
cat(sum(SP.SUI$isLate), "index spells with late operation code (Y712, Y713, Y716, Y717)\n")

# -------------------------------------------------------------------
# draw activity graph
# ------------------------------------------------------------------------

cat("\n")
cat("Generating activity graph\n")
cat("-------------------------\n")
keep <- c("TOT", "TVT", "MS")
FYFreq <- aggregate(SP.SUI[,keep], by=list(SP.SUI$FYEAR), FUN=sum)

pubfig("activity")
gp <- par(no.readonly=T)
layout(matrix(c(1,2), 2, 1, byrow=T), heights=c(1.5,1))

par(mar=c(0,4,4,2))
x <- c(1.5,2.5,3.5,4.5,5.5,6.5,7.5,8.5)
plot(x, FYFreq$TOT, type="b", pch=17,
     xaxt="n",
     xlab="",
     ylab="No. of procedures (TVT/TOT)",
     ylim = c(0,8500),
     cex.axis=0.7,
     cex.lab=1.0,
     las=1)
points(x, FYFreq$TVT, pch=16, type='b')

par(mar=c(5,4,0,2))
```

```
plot(x,FYFreq$MS, type="b", pch=3, xaxt="n", xlab="Financial Year",
     ylab="No. of procedures (SS)",
     ylim=c(0,225), cex.lab=1.0, cex.axis=0.7, las=1)
xlabels = c("2007/08","2008/09","2009/10","2010/11","2011/12","2012/13","2013/14", "2014/15")
axis(side=1, at=x, labels=xlabels, las=1, cex.axis=0.7)

par(gp)
dev.off()
cat("Activity graph written to activity.[pdf|eps]")
cat("\n")

# -----------------------------------------
# additional information about index spells
# -----------------------------------------

cat("\n")
cat("Index spells with cystoscopy\n")
cat("----------------------------\n")

# Number with endoscopic examination of bladder/urethra
bladder.codes <- quote(M45X|M450|M451|M452|M453|M454|M455|M456|M457|M458|M459)
urethra.codes <- quote(M77X|M770|M771|M772|M773|M774|M775|M776|M777|M778|M779)

IEP.SUI$M45 <- sapply(IEP.SUI$OPERTN, FUN=epimatch, e.codes=bladder.codes)
df <- with(IEP.SUI, {aggregate(M45, by=list(SPELL), FUN=any)})
names(df) <- c("SPELL", "M45")
SP.SUI <- merge(SP.SUI, df, by="SPELL", sort=T)
rm(df)
IEP.SUI$M45 <- NULL
cat(sum(SP.SUI$M45), "index spells with endoscopy of bladder\n")

IEP.SUI$M77 <- sapply(IEP.SUI$OPERTN, FUN=epimatch, e.codes=urethra.codes)
df <- with(IEP.SUI, {aggregate(M77, by=list(SPELL), FUN=any)})
names(df) <- c("SPELL", "M77")
SP.SUI <- merge(SP.SUI, df, by="SPELL", sort=T)
rm(df)
IEP.SUI$M77 <- NULL
cat(sum(SP.SUI$M77), "index spells with endoscopy of urethra\n")

# either
SP.SUI$M45M77 <- SP.SUI$M45 | SP.SUI$M77
cat(sum(SP.SUI$M45M77), "index spells with endoscopy of either bladder or urethra\n")

# catheterisations
cath.codes <- quote(M302 | M382 | M471 | M474 | M478 | M479 | M481)
IEP.SUI$cath <- sapply(IEP.SUI$OPERTN, FUN=epimatch, e.codes=cath.codes)
df <- with(IEP.SUI, {aggregate(cath, by=list(SPELL), FUN=any)})
names(df) <- c("SPELL", "cath")
SP.SUI <- merge(SP.SUI, df, by="SPELL", sort=T)
rm(df)
IEP.SUI$cath <- NULL
cat(sum(SP.SUI$cath), "index spells with catheterization\n")

# ------------------
# save spell details
# ------------------
save(SP.SUI, IEP.SUI, which.comp, spells.comp, file="data/indexprocs.Rdata")
rm(list=ls())
```

S5.2.7 split.R

```r
# ---------------------------------------------------------------------------
# split.R
#
# Description:
# ============
# Splits cohort into confounded and unconfounded.
#
# History:
# ========
# 12.05.2016. A.J. Sims. Created.
# 19.05.2016. AJS/KK.    Added rescue procedures to whitelist for spells with
#                        a complication.
# 20.05.2016. AJS/KK.    Updated whitelist.
# ---------------------------------------------------------------------------

rm(list=ls())
source("R/inChapters.R")
source("R/inSections.R")
source("R/opcssplit.R")
source("R/opcs_has_supp.R")
source("R/icdsplit.R")

load("data/indexprocs.Rdata")

# --------------------------------------------------------------------------
# confounding operations
# --------------------------------------------------------------------------
cat("\n")
cat("Split cohort by presence of confounding procedure\n")
cat("------------------------------------------------\n")

# 'white-list' chapters
wlChapters <- c("U")

# 'white-list' sections
wlSections <- c("L72", "L95",
                "M11", "M30", "M45", "M77","M85",
                "Q18", "Q39", "Q50",
                "A55",
                "Q55",
                "E85", "E86", "E87", "E88", "E89", "E90", "E91",
                "E92", "E93", "E94", "E95", "E96", "E97", "E98",
                "A52",
                "Y02", "Y21","Y22","Y25","Y31","Y32","Y41","Y50",
                "Y52","Y53","Y70","Y75","Y76","Y80","Y81","Y82",
                "Y83","Y84","Y85","Y86","Y87","Y88","Y89","Y90",
                "Y91","Y92","Y93","Y94","Y97","Y98","Y99",
                "Z31","Z41","Z42","Z44","Z53",
                "M47")

# 'white-list' codes
wlCodes <- c("A843",
             "H444",
             "M496",
             "P273",
             "M832",
             "P278",
             "S578",
             "T317",
             "O161",
             "L912","L914","L997",
             "M492","M493",
             "S421","S422","S423","S428","S429",
```

```r
              "S476",
              "S574","S575","S576","S577",
              "X292","X308","X309","X312","X569"
)

# SUI codes
suiCodes <- c("M521", "M533", "M536")

# 'rescue'procedures
rescueCodes <- c(
  'E423',        #Temporary tracheostomy
  'E427',        #Removal of tracheostomy tube
  'L703',        #Ligation of artery NEC
  'M373',        #Repair of rupture of bladder
  'M378',        #Other specified other repair of bladder
  'M379',        #Unspecified other repair of bladder
  'M392',        #Open removal of foreign body from bladder
  'M422',        #Endoscopic cauterisation of lesion of bladder
  'M443',        #Endoscopic removal of foreign body from bladder
  'M444',        #Endoscopic removal of blood clot from bladder
  'M737',        #Repair of rupture of urethra NEC
  'M738',        #Other specified repair of urethra
  'M739',        #Unspecified repair of urethra
  'P203',        #Cauterisation of lesion of vagina
  'P271',        #Evacuation of haematoma from vagina
  'P294',        #Removal of foreign body from vagina
  'S111',        #Cauterisation of lesion of skin NEC
  'S424',        #Resuture of skin NEC
  'S571',        #Debridement of skin NEC
  'T301',        #Reopening of abdomen and re-exploration of intra-abdominal operation site and
          #surgical arrest of postoperative bleeding
  'T302',        #Reopening of abdomen and re-exploration of intra-abdominal operation site NEC
  'T303',        #Reopening of abdomen NEC
  'T304',        #Opening of abdomen and exploration of groin
  'T308',        #Other specified opening of abdomen
  'T309',        #Unspecified opening of abdomen
  'X332',        #Intravenous blood transfusion of packed cells
  'X339',        #Unspecified other blood transfusion
  'X341',        #Transfusion of coagulation factor
  'X342',        #Transfusion of plasma NEC
  'X403',        #Haemodialysis NEC
  'X404',        #Haemofiltration
  'X501',        #Direct current cardioversion
  'X502',        #External cardioversion NEC
  'X503',        #Advanced cardiac pulmonary resuscitation
  'X509',        #Unspecified external resuscitation
  'X355'         #Intravenous injection of antimicrobial therapy'
)

# Function to apply whitelists for confounding operations
confounding.ops <- function(codes, allow.rescue=F) {

  # an empty character vector (if no confounding ops)
  no.ops <- character(0)

  # to start, all codes might confound
  confcodes <- codes
  if (length(confcodes)==0) return(no.ops)

  # remove NA codes
  mat <- (confcodes %in% c(NA))
  confcodes <- confcodes[which(mat==F)]
  if (length(confcodes)==0) return(no.ops)
```

```
  # remove any sui codes
  mat <- (confcodes %in% suiCodes)
  confcodes <- confcodes[which(mat==F)]
  if (length(confcodes)==0) return(no.ops)

  # remove rescue codes, if they are allowed
  if (allow.rescue) {
    mat <- (confcodes %in% rescueCodes)
    confcodes <- confcodes[which(mat==F)]
    if (length(confcodes)==0) return(no.ops)
  }

  # are any codes in white list chapters?
  mat <- (confcodes %inChapters% wlChapters)
  confcodes <- confcodes[which(mat==F)]
  if (length(confcodes)==0) return(no.ops)

  # are any codes in white list sections?
  mat <- (confcodes %inSections% wlSections)
  confcodes <- confcodes[which(mat==F)]
  if (length(confcodes)==0) return(no.ops)

  # are any codes in white list codes?
  mat <- (confcodes %in% wlCodes)
  confcodes <- confcodes[which(mat==F)]
  if (length(confcodes)==0) return(no.ops)

  return(confcodes)
}

# apply function to eligible spells (ignoring rescue procedures)
IEP.SUI$confound.norescue <- sapply(IEP.SUI$OPERTN, confounding.ops)
IEP.SUI$confound.norescue.n <- sapply(IEP.SUI$confound.norescue, FUN=length)

# apply to eligible spells accounting for rescue procedures
IEP.SUI$confound.rescue <- sapply(IEP.SUI$OPERTN, confounding.ops, allow.rescue=T)
IEP.SUI$confound.rescue.n <- sapply(IEP.SUI$confound.rescue, FUN=length)

# mark whether episode is in a bad spell (one with a complication)
IEP.SUI$inBadSpell <- IEP.SUI$SPELL %in% SP.SUI$SPELL[SP.SUI$hasComplication]
cat(sum(IEP.SUI$inBadSpell), 'episodes in bad spells\n')

# apply confounding logic
keep <- c('confound.norescue.n', 'confound.rescue.n', 'inBadSpell')
IEP.SUI$isConfounded <- apply(IEP.SUI[,keep], 1, FUN=function(row) {
  is.conf <- F
  if (!row['inBadSpell']) {
    is.conf <- (row['confound.norescue.n'] > 0)
  }
  else {
    is.conf <- (row['confound.rescue.n'] > 0)
  }
  return(is.conf)
})

# identify spells with confounding operations (in any episode)
df <- with(IEP.SUI, {aggregate(isConfounded, by=list(SPELL), FUN=any)})
names(df) <- c("SPELL", "isConfounded")
SP.SUI <- merge(SP.SUI, df, by="SPELL", sort=T)
rm(df)
cat(nrow(SP.SUI), "eligible index spells\n")
cat('  ', sum(!SP.SUI$isConfounded), "index spells without confounding operation\n")
```

```
cat('  ', sum(SP.SUI$isConfounded), "index spells with confounding operation\n")
cat("\n")

# treat combinations as confounded
cat("Unconfounded spells with combination treated as confounded\n")
SP.SUI$isConfounded <- SP.SUI$isConfounded==T | SP.SUI$comb==T
cat('  ', sum(!SP.SUI$isConfounded), "index spells without confounding operation\n")
cat('  ', sum(SP.SUI$isConfounded), "index spells with confounding operation\n")
cat("\n")

# summary of episodes
cat(sum(IEP.SUI$isConfounded & IEP.SUI$eligible), "eligible index spell episodes",
    "with confounding operation\n")
cat(sum(!IEP.SUI$isConfounded & IEP.SUI$eligible), "eligible index spell episodes",
    "without confounding operation\n")
cat('\n')


# tabulate the list of confounding operations
allconf.4 <- unlist(IEP.SUI$confound.norescue)
allconf.3 <- substr(allconf.4, 1, 3)
df <- as.data.frame(table(allconf.3))
names(df) <- c("OPCS section", "Frequency")
cat("Confounding procedures in index spells (by OPCS-4 section)\n")
cat("----------------------------------------------------------\n")
cat("(Potential rescue procedures included in this list)\n")
print(df, row.names=F)
cat("\n")

allconf.1 <- substr(allconf.4, 1, 1)
df <- as.data.frame(table(allconf.1))
names(df) <- c("OPCS chapter", "Frequency")
cat("Confounding procedures in index spells (by OPCS-4 chapter)\n")
cat("----------------------------------------------------------\n")
cat("(Potential rescue procedures included in this list)\n")
print(df, row.names=F)
cat("\n")

cat("\n")
cat("Summary of split cohorts\n")
cat("------------------------\n")


# summary
cat(sum(SP.SUI$TOT), "index spells with TOT introduction only\n")
cat('  ', sum(SP.SUI$TOT & !SP.SUI$isConfounded),
    "unconfounded index spells with TOT introduction only\n")
cat('  ', sum(SP.SUI$TOT & SP.SUI$isConfounded),
    "confounded index spells with TOT introduction only\n")

cat(sum(SP.SUI$TVT), "index spells with TVT introduction only\n")
cat('  ', sum(SP.SUI$TVT & !SP.SUI$isConfounded),
    "unconfounded index spells with TVT introduction only\n")
cat('  ', sum(SP.SUI$TVT & SP.SUI$isConfounded),
    "confounded index spells with TVT introduction only\n")

cat(sum(SP.SUI$MS), "index spells with minisling introduction only\n")
cat('  ', sum(SP.SUI$MS & !SP.SUI$isConfounded),
    "unconfounded index spells with minisling introduction only\n")
cat('  ', sum(SP.SUI$MS & SP.SUI$isConfounded),
    "confounded index spells with minisling introduction only\n")

cat(sum(SP.SUI$comb), "index spells with combination (or none)\n")
```

```r
cat('  ', sum(SP.SUI$comb & !SP.SUI$isConfounded),
    "unconfounded index spells with combination (or none)\n")
cat('  ', sum(SP.SUI$comb & SP.SUI$isConfounded),
    "confounded index spells with combination (or none)\n")

# -----------------------------------------------------
# characteristics of confounded and unconfounded cohorts
# -----------------------------------------------------

cat("\n")
cat("Unconfounded cohort characteristics\n")
cat("-----------------------------------\n")
ss <- subset(SP.SUI, (!SP.SUI$isConfounded))
cat(nrow(ss), "all patients\n")
q <- quantile(ss$STARTAGE, probs=c(0.25, 0.5, 0.75))
cat("  ", q[2], "(", q[1], "to", q[3],") median (IQR) age, years\n")
cat("  ", length(unique(ss$PROCODE3)), "different providers\n")
cat("  ", sum(ss$ADMIMETH %in% c('11', '12', '13', '81')), "elective admissions\n")
q <- quantile(ss$LoS, probs=c(0.25, 0.5, 0.75),na.rm=T)
cat("  ", q[2], "(", q[1], "to", q[3],") median (IQR) LoS, days\n")
los2 <- length(which(ss$LoS>1))
cat('  ', los2, "/", length(ss$LoS),
    "(", round(100.0*los2/length(ss$LoS),2), "%) staying longer than 1 night\n")
cat("   LoS distribution:")
ct <- as.data.frame(table(ss$LoS))
ct <- cbind(rep(c('  '), nrow(ct)), ct)
names(ct) <- c('', 'LoS', 'freq')
print(ct, row.names=F)

ss <- subset(SP.SUI, (!SP.SUI$comb & !SP.SUI$isConfounded))
cat(nrow(ss), "patients with one of TVT, TOT or MS\n")
q <- quantile(ss$STARTAGE, probs=c(0.25, 0.5, 0.75))
cat("  ", q[2], "(", q[1], "to", q[3],") median (IQR) age, years\n")
cat("  ", length(unique(ss$PROCODE3)), "different providers\n")
cat("  ", sum(ss$ADMIMETH %in% c('11', '12', '13', '81')), "elective admissions\n")
q <- quantile(ss$LoS, probs=c(0.25, 0.5, 0.75),na.rm=T)
cat("  ", q[2], "(", q[1], "to", q[3],") median (IQR) LoS, days\n")
los2 <- length(which(ss$LoS>1))
cat('  ', los2, "/", length(ss$LoS),
    "(", round(100.0*los2/length(ss$LoS),2), "%) staying longer than 1 night\n")
cat("   LoS distribution:")
ct <- as.data.frame(table(ss$LoS))
ct <- cbind(rep(c('  '), nrow(ct)), ct)
names(ct) <- c('', 'LoS', 'freq')
print(ct, row.names=F)

ss <- subset(SP.SUI, (SP.SUI$TOT & !SP.SUI$isConfounded))
cat(nrow(ss), "patients with TOT only\n")
q <- quantile(ss$STARTAGE, probs=c(0.25, 0.5, 0.75))
cat("  ", q[2], "(", q[1], "to", q[3],") median (IQR) age, years\n")
cat("  ", length(unique(ss$PROCODE3)), "different providers\n")
cat("  ", sum(ss$ADMIMETH %in% c('11', '12', '13', '81')), "elective admissions\n")
q <- quantile(ss$LoS, probs=c(0.25, 0.5, 0.75),na.rm=T)
cat("  ", q[2], "(", q[1], "to", q[3],") median (IQR) LoS, days\n")
cat("  ", sum(ss$DISMETH %in% c('4')), "in-hospital deaths\n")
cat("  ", sum(ss$M45), "admissions with endoscopic examination of bladder (M45)\n")
cat("  ", sum(ss$M77), "admissions with endoscopic examination of urethra (M77)\n")
cat("  ", sum(ss$M45M77), "admissions with endoscopic examination of urethra or bladder\n")
cat("  ", sum(ss$cath), "admissions with catheterization\n")

ss <- subset(SP.SUI, (SP.SUI$TVT & !SP.SUI$isConfounded))
cat(nrow(ss), "patients with TVT only\n")
q <- quantile(ss$STARTAGE, probs=c(0.25, 0.5, 0.75))
```

```
cat("  ", q[2], "(", q[1], "to", q[3],") median (IQR) age, years\n")
cat("  ", length(unique(ss$PROCODE3)), "different providers\n")
cat("  ", sum(ss$ADMIMETH %in% c('11', '12', '13', '81')), "elective admissions\n")
q <- quantile(ss$LoS, probs=c(0.25, 0.5, 0.75),na.rm=T)
cat("  ", q[2], "(", q[1], "to", q[3],") median (IQR) LoS, days\n")
cat("  ", sum(ss$DISMETH %in% c('4')), "in-hospital deaths\n")
cat("  ", sum(ss$M45), "admissions with endoscopic examination of bladder (M45)\n")
cat("  ", sum(ss$M77), "admissions with endoscopic examination of urethra (M77)\n")
cat("  ", sum(ss$M45M77), "admissions with endoscopic examination of urethra or bladder\n")
cat("  ", sum(ss$cath), "admissions with catheterization\n")

ss <- subset(SP.SUI, (SP.SUI$MS & !SP.SUI$isConfounded))
cat(nrow(ss), "patients with minisling only\n")
q <- quantile(ss$STARTAGE, probs=c(0.25, 0.5, 0.75))
cat("  ", q[2], "(", q[1], "to", q[3],") median (IQR) age, years\n")
cat("  ", length(unique(ss$PROCODE3)), "different providers\n")
cat("  ", sum(ss$ADMIMETH %in% c('11', '12', '13', '81')), "elective admissions\n")
q <- quantile(ss$LoS, probs=c(0.25, 0.5, 0.75),na.rm=T)
cat("  ", q[2], "(", q[1], "to", q[3],") median (IQR) LoS, days\n")
cat("  ", sum(ss$DISMETH %in% c('4')), "in-hospital deaths\n")
cat("  ", sum(ss$M45), "admissions with endoscopic examination of bladder (M45)\n")
cat("  ", sum(ss$M77), "admissions with endoscopic examination of urethra (M77)\n")
cat("  ", sum(ss$M45M77), "admissions with endoscopic examination of urethra or bladder\n")
cat("  ", sum(ss$cath), "admissions with catheterization\n")

ss <- subset(SP.SUI, (SP.SUI$comb & !SP.SUI$isConfounded))
cat(nrow(ss), "patients with combination of TVT, TOT, MS\n")
q <- quantile(ss$STARTAGE, probs=c(0.25, 0.5, 0.75))
cat("  ", q[2], "(", q[1], "to", q[3],") median (IQR) age, years\n")
cat("  ", length(unique(ss$PROCODE3)), "different providers\n")
cat("  ", sum(ss$ADMIMETH %in% c('11', '12', '13', '81')), "elective admissions\n")
q <- quantile(ss$LoS, probs=c(0.25, 0.5, 0.75),na.rm=T)
cat("  ", q[2], "(", q[1], "to", q[3],") median (IQR) LoS, days\n")
cat("  ", sum(ss$DISMETH %in% c('4')), "in-hospital deaths\n")
cat("  ", sum(ss$M45), "admissions with endoscopic examination of bladder (M45)\n")
cat("  ", sum(ss$M77), "admissions with endoscopic examination of urethra (M77)\n")
cat("  ", sum(ss$M45M77), "admissions with endoscopic examination of urethra or bladder\n")
cat("  ", sum(ss$cath), "admissions with catheterization\n")

cat("\n")

cat("Confounded cohort characteristics\n")
cat("---------------------------------\n")
ss <- subset(SP.SUI, (SP.SUI$isConfounded))
cat(nrow(ss), "all patients\n")
q <- quantile(ss$STARTAGE, probs=c(0.25, 0.5, 0.75))
cat("  ", q[2], "(", q[1], "to", q[3],") median (IQR) age, years\n")
cat("  ", length(unique(ss$PROCODE3)), "different providers\n")
cat("  ", sum(ss$ADMIMETH %in% c('11', '12', '13', '81')), "elective admissions\n")
q <- quantile(ss$LoS, probs=c(0.25, 0.5, 0.75),na.rm=T)
cat("  ", q[2], "(", q[1], "to", q[3],") median (IQR) LoS, days\n")
los2 <- length(which(ss$LoS>1))
cat('  ', los2, "/", length(ss$LoS),
    "(", round(100.0*los2/length(ss$LoS),2), "%) staying longer than 1 night\n")
cat("   LoS distribution:")
ct <- as.data.frame(table(ss$LoS))
ct <- cbind(rep(c('  '), nrow(ct)), ct)
names(ct) <- c('', 'LoS', 'freq')
print(ct, row.names=F)

ss <- subset(SP.SUI, (!SP.SUI$comb & SP.SUI$isConfounded))
cat(nrow(ss), "patients with one of TVT, TOT or MS\n")
q <- quantile(ss$STARTAGE, probs=c(0.25, 0.5, 0.75))
```

```
cat("  ", q[2], "(", q[1], "to", q[3],") median (IQR) age, years\n")
cat("  ", length(unique(ss$PROCODE3)), "different providers\n")
cat("  ", sum(ss$ADMIMETH %in% c('11', '12', '13', '81')), "elective admissions\n")
q <- quantile(ss$LoS, probs=c(0.25, 0.5, 0.75),na.rm=T)
cat("  ", q[2], "(", q[1], "to", q[3],") median (IQR) LoS, days\n")
los2 <- length(which(ss$LoS>1))
cat('  ', los2, "/", length(ss$LoS),
    "(", round(100.0*los2/length(ss$LoS),2), "%) staying longer than 1 night\n")
cat("   LoS distribution:")
ct <- as.data.frame(table(ss$LoS))
ct <- cbind(rep(c('  '), nrow(ct)), ct)
names(ct) <- c('', 'LoS', 'freq')
print(ct, row.names=F)

ss <- subset(SP.SUI, (SP.SUI$TOT & SP.SUI$isConfounded))
cat(nrow(ss), "patients with TOT only\n")
q <- quantile(ss$STARTAGE, probs=c(0.25, 0.5, 0.75))
cat("  ", q[2], "(", q[1], "to", q[3],") median (IQR) age, years\n")
cat("  ", length(unique(ss$PROCODE3)), "different providers\n")
cat("  ", sum(ss$ADMIMETH %in% c('11', '12', '13', '81')), "elective admissions\n")
q <- quantile(ss$LoS, probs=c(0.25, 0.5, 0.75),na.rm=T)
cat("  ", q[2], "(", q[1], "to", q[3],") median (IQR) LoS, days\n")
cat("  ", sum(ss$DISMETH %in% c('4')), "in-hospital deaths\n")
cat("  ", sum(ss$M45), "admissions with endoscopic examination of bladder (M45)\n")
cat("  ", sum(ss$M77), "admissions with endoscopic examination of urethra (M77)\n")
cat("  ", sum(ss$M45M77), "admissions with endoscopic examination of urethra or bladder\n")
cat("  ", sum(ss$cath), "admissions with catheterization\n")

ss <- subset(SP.SUI, (SP.SUI$TVT & SP.SUI$isConfounded))
cat(nrow(ss), "patients with TVT only\n")
q <- quantile(ss$STARTAGE, probs=c(0.25, 0.5, 0.75))
cat("  ", q[2], "(", q[1], "to", q[3],") median (IQR) age, years\n")
cat("  ", length(unique(ss$PROCODE3)), "different providers\n")
cat("  ", sum(ss$ADMIMETH %in% c('11', '12', '13', '81')), "elective admissions\n")
q <- quantile(ss$LoS, probs=c(0.25, 0.5, 0.75),na.rm=T)
cat("  ", q[2], "(", q[1], "to", q[3],") median (IQR) LoS, days\n")
cat("  ", sum(ss$DISMETH %in% c('4')), "in-hospital deaths\n")
cat("  ", sum(ss$M45), "admissions with endoscopic examination of bladder (M45)\n")
cat("  ", sum(ss$M77), "admissions with endoscopic examination of urethra (M77)\n")
cat("  ", sum(ss$M45M77), "admissions with endoscopic examination of urethra or bladder\n")
cat("  ", sum(ss$cath), "admissions with catheterization\n")

ss <- subset(SP.SUI, (SP.SUI$MS & SP.SUI$isConfounded))
cat(nrow(ss), "patients with minisling only\n")
q <- quantile(ss$STARTAGE, probs=c(0.25, 0.5, 0.75))
cat("  ", q[2], "(", q[1], "to", q[3],") median (IQR) age, years\n")
cat("  ", length(unique(ss$PROCODE3)), "different providers\n")
cat("  ", sum(ss$ADMIMETH %in% c('11', '12', '13', '81')), "elective admissions\n")
q <- quantile(ss$LoS, probs=c(0.25, 0.5, 0.75),na.rm=T)
cat("  ", q[2], "(", q[1], "to", q[3],") median (IQR) LoS, days\n")
cat("  ", sum(ss$DISMETH %in% c('4')), "in-hospital deaths\n")
cat("  ", sum(ss$M45), "admissions with endoscopic examination of bladder (M45)\n")
cat("  ", sum(ss$M77), "admissions with endoscopic examination of urethra (M77)\n")
cat("  ", sum(ss$M45M77), "admissions with endoscopic examination of urethra or bladder\n")
cat("  ", sum(ss$cath), "admissions with catheterization\n")

ss <- subset(SP.SUI, (SP.SUI$comb & SP.SUI$isConfounded))
cat(nrow(ss), "patients with combination of TVT, TOT, MS\n")
q <- quantile(ss$STARTAGE, probs=c(0.25, 0.5, 0.75))
cat("  ", q[2], "(", q[1], "to", q[3],") median (IQR) age, years\n")
cat("  ", length(unique(ss$PROCODE3)), "different providers\n")
cat("  ", sum(ss$ADMIMETH %in% c('11', '12', '13', '81')), "elective admissions\n")
q <- quantile(ss$LoS, probs=c(0.25, 0.5, 0.75),na.rm=T)
```

```
cat("  ", q[2], "(", q[1], "to", q[3],") median (IQR) LoS, days\n")
cat("  ", sum(ss$DISMETH %in% c('4')), "in-hospital deaths\n")
cat("  ", sum(ss$M45), "admissions with endoscopic examination of bladder (M45)\n")
cat("  ", sum(ss$M77), "admissions with endoscopic examination of urethra (M77)\n")
cat("  ", sum(ss$M45M77), "admissions with endoscopic examination of urethra or bladder\n")
cat("  ", sum(ss$cath), "admissions with catheterization\n")

# ------------------
# save spell details
# ------------------
save(SP.SUI, file="data/split.Rdata")
rm(list=ls())
```

S5.2.8 pericomp.R

```
# ----------------------------------------------------------------------------
# pericomp.R
#
# Description:
# ============
# Estimates peri-procedural complication rates.
#
# History:
# ========
# 29.02.2015. A.J. Sims. Created.
# 18.08.2015. A.J. Sims. Uses updated epimatch.
# 07.12.2015. A.J. Sims. Presence of additional main codes for repairs,
#                        renewals (e.g. M528) now tested in which.tape
#                        function.
# 10.12.2015. A.J. Sims. Added KK's additional analysis from perianalyis.R
#                        to write out additional details of cohorts and
#                        complications in confounded cohorts.
# 14.12.2015. A.J. Sims. Part to identify index spells moved to indexprocs.R.
# 15.12.2015. A.J. Sims. Speeded up identification of complications.
# ----------------------------------------------------------------------------

rm(list=ls())
source("R/epimatch.R")
source("R/inChapters.R")
source("R/inSections.R")
source("R/opcssplit.R")
source("R/opcs_has_supp.R")
source("R/icdsplit.R")
source("R/is_complication.R")
source("R/icdSubchapters.R")
source("printcomp.R")

load("data/indexprocs.Rdata")
rm(SP.SUI)
load("data/split.Rdata")

cat("\n")
cat("Summary of peri-procedural complications in both parts of cohort\n")
cat("----------------------------------------------------------------\n")

# summary statistics
cat(sum(SP.SUI$hasComplication), "spells with complication\n")
cat('  ', sum(SP.SUI$hasComplication & !SP.SUI$isConfounded),
    "spells in unconfounded operations\n")
cat('    ', sum(SP.SUI$TOT & !SP.SUI$isConfounded & SP.SUI$hasComplication),
    "TOT\n")
```

```r
cat('    ', sum(SP.SUI$TVT & !SP.SUI$isConfounded & SP.SUI$hasComplication),
    "TVT\n")
cat('    ', sum(SP.SUI$MS & !SP.SUI$isConfounded & SP.SUI$hasComplication),
    "minisling\n")
cat('    ', sum(SP.SUI$comb & !SP.SUI$isConfounded & SP.SUI$hasComplication),
    "combination\n")
cat('  ', sum(SP.SUI$hasComplication & SP.SUI$isConfounded),
    "spells in confounded operations\n")
cat('    ', sum(SP.SUI$TOT & SP.SUI$isConfounded & SP.SUI$hasComplication),
    "TOT\n")
cat('    ', sum(SP.SUI$TVT & SP.SUI$isConfounded & SP.SUI$hasComplication),
    "TVT\n")
cat('    ', sum(SP.SUI$MS & SP.SUI$isConfounded & SP.SUI$hasComplication),
    "minisling\n")
cat('    ', sum(SP.SUI$comb & SP.SUI$isConfounded & SP.SUI$hasComplication),
    "combination\n")

# -------------------------------------------------------------------------
# add extra columns and tidy up which.comp so it can be analysed separately
# -------------------------------------------------------------------------

# add information about spells
keep <- c("PSEUDO_HESID", "TOT", "TVT", "MS", "comb", "isConfounded")
which.comp <- merge(which.comp, SP.SUI[,keep], by=c("PSEUDO_HESID"), all.x=T)

# add ICD-10 subchapter and descriptions
which.comp$ICD <- with(which.comp, ifelse(is.na(MainCode), CompCode, MainCode))
which.comp$subchapter <- sapply(which.comp$ICD, FUN=icd10.subchapter)
which.comp$Description <- sapply(which.comp$ICD, FUN=icd10.description)

# print complication summary
which.comp <- printcomp(which.comp)

# -----------------------------------------------------------------------------
# additional complication analysis, aggregated by subchapter and total combined
# -----------------------------------------------------------------------------
cat("additional complication analysis - see 'table' folder\n")

# all complication codes - Unconfounded
ss <- which.comp[(which.comp$isConfounded==F & is.na(which.comp$SuppCode)) |
                 (which.comp$isConfounded==F & !is.na(which.comp$SuppCode)) |
                 (which.comp$isConfounded==F),]

if (nrow(ss) > 0) {
  ct <- with(ss, {aggregate(cbind(TVT, TOT, MS), by=list(subchapter,Description), FUN=sum)})
  if (nrow(ct)>0) {
    names(ct) <- c("subchapter", "Description","TVT", "TOT", "MS")
    Comp_Unconf_ALL <- ct[order(ct$subchapter),]
    write.csv(Comp_Unconf_ALL,"table/Comp_Unconf_ALL.csv",row.names=F)
  }
}
cat("\n")

# all complication codes - Confounded
ss <- which.comp[(which.comp$isConfounded==T & is.na(which.comp$SuppCode)) |
                   (which.comp$isConfounded==T & !is.na(which.comp$SuppCode)) |
                   (which.comp$isConfounded==T),]
if (nrow(ss) > 0) {
  ct <- with(ss, {aggregate(cbind(TVT, TOT, MS), by=list(subchapter,Description), FUN=sum)})
  if (nrow(ct)>0) {
    names(ct) <- c("subchapter", "Description","TVTc", "TOTc", "MSc")
    Comp_Conf_ALL <- ct[order(ct$subchapter),]
    write.csv(Comp_Conf_ALL,"table/Comp_Conf_ALL.csv",row.names=F)
```

```
  }
}
cat("\n")

# combined
ALL_COMP_subchap = merge(Comp_Unconf_ALL, Comp_Conf_ALL,
                         by=c("subchapter","Description"),all=T)
ALL_COMP_subchap[is.na(ALL_COMP_subchap)] <- 0
write.csv(ALL_COMP_subchap,"table/ALL_COMP_subchap.csv")

ALL_COMP_subchap$Chapter = substr(ALL_COMP_subchap$subchapter,1,1)
ALL_COMP_chap = with(ALL_COMP_subchap,
                     { aggregate(cbind(TVT,TOT,MS,TVTc,TOTc,MSc), by=list(Chapter),
                                 FUN=sum)})
names(ALL_COMP_chap)[1] = c("Chapter")
write.csv(ALL_COMP_chap,"table/ALL_COMP_chap.csv", row.names=F)

# ---------------------------------------------
# save peri-procedural complications information
# ---------------------------------------------
save(SP.SUI, which.comp, file="data/pericomp.Rdata")

rm(list=ls())
```

S5.2.9 thirty.R

```
# ------------------------------------------------------------------------
# thirty.R
#
# Description:
# ============
# Analysis of 30 day readmissions.
#
# History:
# ========
# 06.06.2016. A.J. Sims. Created from periproc.R.
# 07.06.2016. A.J. Sims. Added analysis of non-complication 30 day
#                        admissions from KK code.
# 14.06.2016. A.J. Sims. Use functions in interventions.R to detect 30 day
#                        admissions for further intervention.
# 24.06.2016. A.J. Sims. Added reasons for routine admission by mesh type.
# ------------------------------------------------------------------------

# clear
rm(list=ls())

# libraries & functions
source("R/epimatch.R")
source("R/opcs_has_supp.R")
source("R/opcssplit.R")
source("R/icdsplit.R")
source("R/inSections.R")
source("R/is_complication.R")
source("R/inChapters.R")
source("R/inSections.R")
source("printcomp.R")
source("interventions.R")

# load data frames
load(file="data/indexprocs.Rdata")
rm(which.comp)
```

```r
rm(SP.SUI)
load(file="data/split.Rdata")
load(file="data/sui-cleaned.Rdata")

# ----------------------------
# 30 day complication analysis
# ----------------------------

# header
cat('\n')
cat('----------------------------\n')
cat('30 day complication analysis\n')
cat('----------------------------\n')
cat("\n")

# add index admission dates for each subject
keep.ind <- c('PSEUDO_HESID', 'SPELL', 'ADMIDATE', 'TOT', 'TVT', 'MS', 'comb', 'isConfounded')
df.idate <- SP.SUI[, keep.ind]
names(df.idate) <- c('PSEUDO_HESID', 'SPELL.INDEX', 'ADMIDATE.INDEX',
                     'TOT', 'TVT', 'MS', 'comb', 'isConfounded')
cat(nrow(df.idate), "eligible index spells with admission date\n")
keep.ep <- c('EPI_ID', 'SPELL', 'PSEUDO_HESID', 'ADMIDATE', 'OPERTN', 'DIAG')
TH <- merge(SUI[, keep.ep], df.idate, by='PSEUDO_HESID')
TH$POD <- TH$ADMIDATE - TH$ADMIDATE.INDEX
cat(nrow(TH), "total episodes for patients with an eligible index spell\n")
rm(df.idate)

# remove episodes before index, or more than 30 days afterwards
TH <- TH[(TH$POD >= 0) & (TH$POD <= 30),]
cat(nrow(TH), "episodes on day of index spell or within 30 days\n")
cat("  ", length(unique(TH$PSEUDO_HESID)), "patients\n")

# mark those having catheter procedures
cath.codes <- quote(M472 | M473 | M475 |
                    M302 | M471 | M474 | M478 | M479 |
                    M382 | M481 | M492 | M493)
TH$cath <- sapply(TH$OPERTN, FUN=epimatch, e.codes=cath.codes)

# and those having further mesh interventions
TH$reop <- sapply(TH$OPERTN, FUN=int.anysui)

# remove index spells
TH <- TH[TH$SPELL != TH$SPELL.INDEX,]

# summary stats
cat(nrow(TH), "episodes on day of index spell or within 30 days,",
              "not including index episodes\n")
cat("  ", length(unique(TH$PSEUDO_HESID)), "patients\n")
cat("    ", length(unique(TH$PSEUDO_HESID[TH$isConfounded==F])), 'unconfounded patients\n')
cat("      ", length(unique(TH$PSEUDO_HESID[TH$isConfounded==F & TH$TOT])), 'TOT\n')
cat("      ", length(unique(TH$PSEUDO_HESID[TH$isConfounded==F & TH$TVT])), 'TVT\n')
cat("      ", length(unique(TH$PSEUDO_HESID[TH$isConfounded==F & TH$MS])), 'MS\n')
cat("      ", length(unique(TH$PSEUDO_HESID[TH$isConfounded==F & TH$comb])), 'comb\n')
cat("    ", length(unique(TH$PSEUDO_HESID[TH$isConfounded==T])), 'confounded patients\n')
cat("      ", length(unique(TH$PSEUDO_HESID[TH$isConfounded==T & TH$TOT])), 'TOT\n')
cat("      ", length(unique(TH$PSEUDO_HESID[TH$isConfounded==T & TH$TVT])), 'TVT\n')
cat("      ", length(unique(TH$PSEUDO_HESID[TH$isConfounded==T & TH$MS])), 'MS\n')
cat("      ", length(unique(TH$PSEUDO_HESID[TH$isConfounded==T & TH$comb])), 'comb\n')
cat("  ", nrow(TH[TH$POD==0,]), "episodes on day zero (day of index admission)\n")
#ct <- table(TH£POD)
#cat("Distribution of admission times (days):\n")
#print(ct)
cat('  ', length(unique(TH$SPELL)), "spells of admission within 30 days\n")
```

```r
cat("    ", length(unique(TH$SPELL[TH$isConfounded==F])), 'spells for unconfounded patients\n')
cat("      ", length(unique(TH$SPELL[TH$isConfounded==F & TH$TOT])), 'TOT\n')
cat("      ", length(unique(TH$SPELL[TH$isConfounded==F & TH$TVT])), 'TVT\n')
cat("      ", length(unique(TH$SPELL[TH$isConfounded==F & TH$MS])), 'MS\n')
cat("      ", length(unique(TH$SPELL[TH$isConfounded==F & TH$comb])), 'comb\n')
cat("    ", length(unique(TH$SPELL[TH$isConfounded==T])), 'spells for confounded patients\n')
cat("      ", length(unique(TH$SPELL[TH$isConfounded==T & TH$TOT])), 'TOT\n')
cat("      ", length(unique(TH$SPELL[TH$isConfounded==T & TH$TVT])), 'TVT\n')
cat("      ", length(unique(TH$SPELL[TH$isConfounded==T & TH$MS])), 'MS\n')
cat("      ", length(unique(TH$SPELL[TH$isConfounded==T & TH$comb])), 'comb\n')

# create normalised complication table
comp.30 <- data.frame(PSEUDO_HESID=NA, SPELL=NA, MainCode=NA, CompCode=NA, SuppCode=NA)

# identify complications from diagnostic codes, one episode per row
is.comp <- function(row) {
  chunks <- icdsplit(unlist(row['DIAG'], use.names=F))  # break into sequences
  chunks <- chunks[1]                                   # only the first
  if (length(chunks) == 0) {
    return(F)
  }
  chunk.MT <- sapply(chunks, FUN=is.complication) # main and trigger codes, if any
  chunk.MT <- as.data.frame(t(chunk.MT))          # in columns
  chunk.MT <- chunk.MT[!is.na(chunk.MT$MainCode) | !is.na(chunk.MT$CompCode),]
  if (nrow(chunk.MT)>0) {
    chunk.MT <- cbind(SPELL=row['SPELL'], PSEUDO_HESID=row['PSEUDO_HESID'], chunk.MT)
    comp.30 <<- rbind(comp.30, chunk.MT)
  }
  return(nrow(chunk.MT)>0)
}

# find episodes with complication
TH$hasComplication <- apply(TH, 1, FUN=is.comp)

# tidy up comp.30
narow <- apply(comp.30, 1, function(x) all(is.na(x)))
comp.30 <- comp.30[!narow, ]

# add information about patients (merge with index spells on PSEUDO_HESID)
keep <- c("PSEUDO_HESID", "TOT", "TVT", "MS", "comb", "isConfounded")
comp.30 <- merge(comp.30, SP.SUI[,keep], by=c("PSEUDO_HESID"), all.x=T)

cat(sum(TH$hasComplication), "thirty day episodes admitted for a complication\n")
bad.spells <- unique(TH$SPELL[TH$hasComplication])
cat("  ", length(bad.spells), "spells\n")
cat("  ", length(unique(TH$PSEUDO_HESID[TH$hasComplication])), "patients\n")
cat("  ", nrow(TH[TH$POD==0 & TH$hasComplication,]),
    "episodes on day zero (day of index admission)\n")

# summary stats
cat(sum(TH$hasComplication & !TH$isConfounded),
    "thirty day episodes admitted for complication following unconfounded operation:\n")
ne <- sum(TH$TOT & !TH$isConfounded & TH$hasComplication)
ns <- length(unique(TH$SPELL[TH$TOT & !TH$isConfounded & TH$hasComplication]))
np <- length(unique(TH$PSEUDO_HESID[TH$TOT & !TH$isConfounded & TH$hasComplication]))
cat('  ', ne, "episodes,", ns, "spells,", np, "patients (TOT)\n")
nrs <- length(unique(TH$SPELL[TH$TOT & !TH$isConfounded & TH$hasComplication & TH$reop]))
nrp <- length(unique(TH$PSEUDO_HESID[TH$TOT & !TH$isConfounded & TH$hasComplication & TH$reop]))
cat('    ', nrs, "spells,", nrp, "patients have re-intervention (TOT)\n")

ne <- sum(TH$TVT & !TH$isConfounded & TH$hasComplication)
ns <- length(unique(TH$SPELL[TH$TVT & !TH$isConfounded & TH$hasComplication]))
np <- length(unique(TH$PSEUDO_HESID[TH$TVT & !TH$isConfounded & TH$hasComplication]))
```

```
cat('  ', ne, "episodes,", ns, "spells,", np, "patients (TVT)\n")
nrs <- length(unique(TH$SPELL[TH$TVT & !TH$isConfounded & TH$hasComplication & TH$reop]))
nrp <- length(unique(TH$PSEUDO_HESID[TH$TVT & !TH$isConfounded & TH$hasComplication & TH$reop]))
cat('    ', nrs, "spells,", nrp, "patients have re-intervention (TVT)\n")

ne <- sum(TH$MS & !TH$isConfounded & TH$hasComplication)
ns <- length(unique(TH$SPELL[TH$MS & !TH$isConfounded & TH$hasComplication]))
np <- length(unique(TH$PSEUDO_HESID[TH$MS & !TH$isConfounded & TH$hasComplication]))
cat('  ', ne, "episodes,", ns, "spells,", np, "patients (MS)\n")
nrs <- length(unique(TH$SPELL[TH$MS & !TH$isConfounded & TH$hasComplication & TH$reop]))
nrp <- length(unique(TH$PSEUDO_HESID[TH$MS & !TH$isConfounded & TH$hasComplication & TH$reop]))
cat('    ', nrs, "spells,", nrp, "patients have re-intervention (MS)\n")

ne <- sum(TH$comb & !TH$isConfounded & TH$hasComplication)
ns <- length(unique(TH$SPELL[TH$comb & !TH$isConfounded & TH$hasComplication]))
np <- length(unique(TH$PSEUDO_HESID[TH$comb & !TH$isConfounded & TH$hasComplication]))
cat('  ', ne, "episodes,", ns, "spells,", np, "patients (comb)\n")
nrs <- length(unique(TH$SPELL[TH$comb & !TH$isConfounded & TH$hasComplication & TH$reop]))
nrp <- length(unique(TH$PSEUDO_HESID[TH$comb & !TH$isConfounded & TH$hasComplication & TH$reop]))
cat('    ', nrs, "spells,", nrp, "patients have re-intervention (comb)\n")

cat(sum(TH$hasComplication & TH$isConfounded),
    "thirty day episodes admitted for complication following confounded operation:\n")
ne <- sum(TH$TOT & TH$isConfounded & TH$hasComplication)
ns <- length(unique(TH$SPELL[TH$TOT & TH$isConfounded & TH$hasComplication]))
np <- length(unique(TH$PSEUDO_HESID[TH$TOT & TH$isConfounded & TH$hasComplication]))
cat('  ', ne, "episodes,", ns, "spells,", np, "patients (TOT)\n")
nrs <- length(unique(TH$SPELL[TH$TOT & TH$isConfounded & TH$hasComplication & TH$reop]))
nrp <- length(unique(TH$PSEUDO_HESID[TH$TOT & TH$isConfounded & TH$hasComplication & TH$reop]))
cat('    ', nrs, "spells,", nrp, "patients have re-intervention (TOT)\n")

ne <- sum(TH$TVT & TH$isConfounded & TH$hasComplication)
ns <- length(unique(TH$SPELL[TH$TVT & TH$isConfounded & TH$hasComplication]))
np <- length(unique(TH$PSEUDO_HESID[TH$TVT & TH$isConfounded & TH$hasComplication]))
cat('  ', ne, "episodes,", ns, "spells,", np, "patients (TVT)\n")
nrs <- length(unique(TH$SPELL[TH$TVT & TH$isConfounded & TH$hasComplication & TH$reop]))
nrp <- length(unique(TH$PSEUDO_HESID[TH$TVT & TH$isConfounded & TH$hasComplication & TH$reop]))
cat('    ', nrs, "spells,", nrp, "patients have re-intervention (TVT)\n")

ne <- sum(TH$MS & TH$isConfounded & TH$hasComplication)
ns <- length(unique(TH$SPELL[TH$MS & TH$isConfounded & TH$hasComplication]))
np <- length(unique(TH$PSEUDO_HESID[TH$MS & TH$isConfounded & TH$hasComplication]))
cat('  ', ne, "episodes,", ns, "spells, ", np, "patients (MS)\n")
nrs <- length(unique(TH$SPELL[TH$MS & TH$isConfounded & TH$hasComplication & TH$reop]))
nrp <- length(unique(TH$PSEUDO_HESID[TH$MS & TH$isConfounded & TH$hasComplication & TH$reop]))
cat('    ', nrs, "spells,", nrp, "patients have re-intervention (MS)\n")

ne <- sum(TH$comb & TH$isConfounded & TH$hasComplication)
ns <- length(unique(TH$SPELL[TH$comb & TH$isConfounded & TH$hasComplication]))
np <- length(unique(TH$PSEUDO_HESID[TH$comb & TH$isConfounded & TH$hasComplication]))
cat('  ', ne, "episodes,", ns, "spells,", np, "patients (comb)\n")
nrs <- length(unique(TH$SPELL[TH$comb & TH$isConfounded & TH$hasComplication & TH$reop]))
nrp <- length(unique(TH$PSEUDO_HESID[TH$comb & TH$isConfounded & TH$hasComplication & TH$reop]))
cat('    ', nrs, "spells,", nrp, "patients have re-intervention (comb)\n")


# print complication summary
comp.30 <- printcomp(comp.30)

# analysis of those admitted within 30 days without a complication
cat("\n")
cat("30 day admissions without complication\n")
cat("--------------------------------------\n")
```

```
"%w/o%" <- function(x, y) x[!x %in% y]
good.spells <- unique(TH$SPELL) %w/o% bad.spells
cat(length(good.spells), "thirty day spells without admission for complication\n")
cohort <- unique(TH$PSEUDO_HESID[TH$SPELL %in% good.spells])
cat("  ", length(cohort), "patients\n")
THNC <- TH[TH$SPELL %in% good.spells,]
THNC <- THNC[order(THNC$PSEUDO_HESID,THNC$ADMIDATE),]
cat("  ", nrow(THNC), "episodes\n")
cat("\n")

# function to summarise 30 day readmissions without complication
th.routine <- function(df) {

  # total number of admissions
  cat(nrow(df), "thirty day episodes\n")
  cat('  ', length(unique(df$SPELL)), "spells\n")
  cat('  ', length(unique(df$PSEUDO_HESID)), "patients\n")

  # those with catheter procedures
  cat(sum(df$cath), "thirty day episodes with catheter-related procedures\n")
  df.cath <- aggregate(df[,"cath"], by=list(df$SPELL), FUN=any)
  names(df.cath) <- c("SPELL", "cath")
  cat('  ', sum(df.cath$cath), "spells involving (but not limited to) catheter procedures\n")

  # those having further mesh interventions
  cat(sum(df$reop), "thirty day episodes with mesh-related re-interventions\n")
  df.reop <- aggregate(df[,"reop"], by=list(df$SPELL), FUN=any)
  names(df.reop) <- c("SPELL", "reop")
  cat('  ', sum(df.reop$reop), "spells involving (but not limited to) mesh re-interventions\n")

  # those with no procedure
  df$noop <- is.na(df$OPERTN)
  cat(sum(df$noop), "thirty day episodes with no procedure\n")
  df.noop <- aggregate(df[,"noop"], by=list(df$SPELL), FUN=all)
  names(df.noop) <- c("SPELL", "noop")
  cat('  ', sum(df.noop$noop), "spells with no procedure\n")

  # discount those with no confounding therapeutic procedures
  df$confound <- sapply(df$OPERTN, FUN=function(codes) {
    wlChapters <- c("U","Y","Z")
    wlSections <- c("A18","A55","E25","E36","E37","E49","E51","E63","G16","G19",
                    "G45","G55","G65","G80","H22","H25","H28","H68","H69","H70",
                    "J09","J13","J25","J43","J44","J45","J67","K51","K58","L72",
                    "L95","M11","M45","M77","M85","Q18","Q39","Q50","R02",
                    "R05","T11","T43","W36","W87","W88",
                    "Q55",
                    "E85", "E86", "E87", "E88", "E89", "E90", "E91",
                    "E92", "E93", "E94", "E95", "E96", "E97", "E98",
                    "O11","O12","O13","O14","O16","O28","O30","O31","O33")
    wlCodes <- c("A843",
                 "H444",
                 "M496",
                 "P273",
                 "M301","M303","M304","M305","M306","M308","M309")

    # an empty character vector (if no confounding ops)
    no.ops <- character(0)

    # to start, all codes might confound
    confcodes <- codes
    if (length(confcodes)==0) return(no.ops)
```

```
    # remove NA codes
    mat <- (confcodes %in% c(NA))
    confcodes <- confcodes[which(mat==F)]
    if (length(confcodes)==0) return(no.ops)

    # are any codes in white list chapters?
    mat <- (confcodes %inChapters% wlChapters)
    confcodes <- confcodes[which(mat==F)]
    if (length(confcodes)==0) return(no.ops)

    # are any codes in white list sections?
    mat <- (confcodes %inSections% wlSections)
    confcodes <- confcodes[which(mat==F)]
    if (length(confcodes)==0) return(no.ops)

    # are any codes in white list codes?
    mat <- (confcodes %in% wlCodes)
    confcodes <- confcodes[which(mat==F)]
    if (length(confcodes)==0) return(no.ops)
    return(confcodes)
  })
  df$noconf <- sapply(df$confound, FUN=function(confcodes) {return(length(confcodes)==0)})
  cat(sum(df$noconf), "episodes with no confounding procedure\n")
  df.noconf <- aggregate(df[,"noconf"], by=list(df$SPELL), FUN=all)
  names(df.noconf) <- c("SPELL", "noconf")
  cat('  ', sum(df.noconf$noconf), "spells no confounding procedure \n")
  cat('  ', sum(df.noconf$noconf)-sum(df.noop$noop),
      "spells with procedures but no confounding procedures \n")
}

cat("30 day readmissions with no complication - whole cohort:\n")
cat("--------------------------------------------------------\n")
th.routine(THNC)
cat("\n")

cat("30 day readmissions with no complication - unconfounded cohort:\n")
cat("---------------------------------------------------------------\n")
th.routine(THNC[!THNC$isConfounded,])
cat("\n")

cat("30 day readmissions with no complication - unconfounded TVT:\n")
cat("------------------------------------------------------------\n")
th.routine(THNC[!THNC$isConfounded & THNC$TVT,])
cat("\n")

cat("30 day readmissions with no complication - unconfounded TOT:\n")
cat("------------------------------------------------------------\n")
th.routine(THNC[!THNC$isConfounded & THNC$TOT,])
cat("\n")

cat("30 day readmissions with no complication - unconfounded MS:\n")
cat("------------------------------------------------------------\n")
th.routine(THNC[!THNC$isConfounded & THNC$MS,])
cat("\n")

cat("30 day readmissions with no complication - confounded cohort:\n")
cat("-------------------------------------------------------------\n")
th.routine(THNC[THNC$isConfounded,])
cat("\n")

cat("30 day readmissions with no complication - confounded TVT:\n")
cat("----------------------------------------------------------\n")
th.routine(THNC[THNC$isConfounded & THNC$TVT,])
```

```
cat("\n")

cat("30 day readmissions with no complication - confounded TOT:\n")
cat("-----------------------------------------------------------\n")
th.routine(THNC[THNC$isConfounded & THNC$TOT,])
cat("\n")

cat("30 day readmissions with no complication - confounded MS:\n")
cat("----------------------------------------------------------\n")
th.routine(THNC[THNC$isConfounded & THNC$MS,])
cat("\n")


# save thirty day details
save(TH, comp.30, file="data/thirty.Rdata")

# --------
# clean up
# --------
rm(list=ls())
```

S5.2.10 postproc.R

```
# --------------------------------------------------------------------------
# postproc.R
#
# Description:
# ============
# Creates post-procedure event tables for survival and history analysis.
#
# History:
# ========
# 29.09.2015. A.J. Sims. Created.
# 03.12.2015. A.J. Sims. Includes all event types; table generation only.
# 06.06.2016. A.J. Sims. 30 day readmisson analysis moved to thirty.R.
# --------------------------------------------------------------------------

# clear
rm(list=ls())

# libraries & functions
source("R/epimatch.R")
source("R/opcs_has_supp.R")
source("R/opcssplit.R")
source("R/icdsplit.R")
source("R/inSections.R")
source("R/is_complication.R")
source("printcomp.R")
source("interventions.R")

# load data frames
load(file="data/indexprocs.Rdata")
rm(which.comp)
rm(SP.SUI)
load(file="data/split.Rdata")
load(file="data/sui-cleaned.Rdata")
load(file="data/thirty.Rdata")
rm(comp.30)

# header
cat('\n')
```

```r
cat('------------------------\n')
cat('Post-procedural analysis\n')
cat('------------------------\n')
cat("\n")

# -------------------------------------------
# count episodes before and after index spells
# -------------------------------------------
cat("Counting episodes pre- and post- index\n")
cat("--------------------------------------\n")

# index admission dates by person
keep.ind <- c('PSEUDO_HESID', 'SPELL', 'ADMIDATE')
df.idate <- SUI[SUI$Index==T, keep.ind]
names(df.idate) <- c('PSEUDO_HESID', 'SPELL.INDEX', 'ADMIDATE.INDEX')

# add index admission details for later comparison
keep.ep <- c('EPI_ID', 'SPELL', 'PSEUDO_HESID', 'ADMIDATE')
NIEP.SUI <- merge(SUI[, keep.ep], df.idate, by='PSEUDO_HESID')
cat(nrow(NIEP.SUI), "total episodes\n")
rm(df.idate)

# count pre-and post (and index) episodes
# nb this assumes anything on or before the data of index is a pre-index
# spell (not always true, but very small error)
lv.pre <- with(NIEP.SUI, (SPELL != SPELL.INDEX) & (ADMIDATE <= ADMIDATE.INDEX))
lv.pst <- with(NIEP.SUI, (SPELL != SPELL.INDEX) & (ADMIDATE > ADMIDATE.INDEX))
lv.ndx <- with(NIEP.SUI, (SPELL == SPELL.INDEX))
cat(sum(lv.ndx), "episodes in index spells\n")
cat(sum(lv.pre), "episodes before index spells\n")
cat(sum(lv.pst), "episodes after index spells\n")
cat("\n")

rm(NIEP.SUI)
rm(lv.ndx)
rm(lv.pre)
rm(lv.pst)

# ---------------------------------
# event table for in-hospital deaths
# ---------------------------------
cat("In-hospital deaths\n")
cat("------------------\n")

# episodes
keep <- c('PSEUDO_HESID', 'SPELL', 'DISDATE')
event.death <- SUI[SUI$DISMETH==4, keep]
cat(nrow(event.death), "in-hospital death episodes\n")

# spells
event.death <- with(event.death, aggregate(DISDATE, by=list(PSEUDO_HESID, SPELL), FUN=min))
names(event.death) <- c('PSEUDO_HESID', 'SPELL', 'date.death')
cat(nrow(event.death), "in-hospital death spells\n")

# add start times (admission date of index procedure)
keep <- c('PSEUDO_HESID', 'ADMIDATE')
event.ndx <- with(SP.SUI, SP.SUI[PSEUDO_HESID %in% event.death$PSEUDO_HESID, keep])
names(event.ndx) <- c('PSEUDO_HESID', 'date.index')
event.death <- merge(event.death, event.ndx, by='PSEUDO_HESID', sort=F)
cat(nrow(event.death), "in-hospital death spells for SUI cohort\n")

# add time and status for survival analysis
event.death$time <- as.numeric(event.death$date.death-event.death$date.index)
```

```
event.death$status <- 0

# order it
event.death <- event.death[order(event.death$PSEUDO_HESID, event.death$time),]

# remove patients who died before index (should be none!)
before <- (event.death$time <= 0)
cat(sum(before), "patients died before index procedure\n")
if (sum(before) > 0) {
  print(event.death[before,])
  event.death <- event.death[!before, , drop=T]
}

# check for patients who died twice
dup <- duplicated(event.death$PSEUDO_HESID)
cat(sum(dup), "duplicated PSEUDO_HESID in death events\n")
if (any(dup)) {
  #who <- event.death£PSEUDO_HESID[dup, drop=T]
  #print(event.death[event.death£PSEUDO_HESID %in% who,])
  event.death <- event.death[!dup,]
}

cat(nrow(event.death), "in-hospital death spells\n")
cat("  ", length(unique(event.death$PSEUDO_HESID)), "different patients\n")
cat(nrow(event.death[event.death$time <= 30,]),
    "in-hospital death spells within 30 days\n")
cat("   ", length(unique(event.death$PSEUDO_HESID[event.death$time<=30])),
    "different patients\n")
cohort.unconf <- with(SP.SUI, PSEUDO_HESID[isConfounded==F])
cat(nrow(event.death[event.death$time <= 30 &
                     event.death$PSEUDO_HESID %in% cohort.unconf,]),
    "in-hospital death spells within 30 days (unconfounded cohort)\n")
cat("   ",
    length(unique(event.death$PSEUDO_HESID[event.death$time<=30 &
                                           event.death$PSEUDO_HESID %in% cohort.unconf])),
    "different patients (unconfounded cohort)\n")

cat("\n")

# -----------------------------------------------------------------------
# event table for time-in-study for subjects who did not die in-hospital
# -----------------------------------------------------------------------
cat("Patients who survived to the end of study\n")
cat("-----------------------------------------\n")

# all patients who survived to study end
keep <- c('PSEUDO_HESID', 'ADMIDATE')
event.alive <- SP.SUI[!(SP.SUI$PSEUDO_HESID %in% event.death$PSEUDO_HESID), keep]
names(event.alive) <- c('PSEUDO_HESID', 'date.index')

# latest date of follow-up
event.alive$date.censor <- max(SUI$ADMIDATE)
event.alive$time <- as.numeric(event.alive$date.censor - event.alive$date.index)
event.alive$status <- 0
cat(nrow(event.alive), "patients alive at end of study period\n")

# anyone in both cohorts?
da <- intersect(event.alive$PSEUDO_HESID, event.death$PSEUDO_HESID)
cat(length(da), "patients who were in both dead and alive cohorts\n")
if (length(da) > 0) {
  print(da)
}
```

```r
# total time in study
n.fu <- nrow(event.death)+nrow(event.alive)
cat(n.fu, "patients in SUI cohort with index procedure\n")
t.fu <- sum(event.death$time)+sum(event.alive$time)
cat(t.fu, "total follow-up (days)\n")
cat(round(t.fu/n.fu, digits=1), "days mean follow-up\n")
cat("\n")

# --------------------------------------------------
# event table for admissions due to relevant symptoms
# --------------------------------------------------
cat("Admissions due to relevant symptoms\n")
cat("-----------------------------------\n")

# admissions for pain
SUI$Pain <- sapply(SUI$DIAG, FUN=function(diag){
  pain.codes <- c('R100', 'R102', 'R103', 'R104', 'R10X',
                  'R300', 'R301', 'R309', 'R30X',
                  'N941')
  pain <- F
  if (diag[1] %in% pain.codes) {
    pain <- T
  }
  return(pain)
})
cat(sum(SUI$Pain), "episodes admitted for pain\n")

# admissions for abnormal bleeding
SUI$Bleeding <- sapply(SUI$DIAG, FUN=function(diag){
  bleeding.codes <- c('N930', 'N938', 'N939', 'N93X')
  bleed <- F
  if (diag[1] %in% bleeding.codes) {
    bleed <- T
  }
  return(bleed)
})
cat(sum(SUI$Bleeding), "episodes admitted for abnormal bleeding\n")

# complications relating to the procedure, including injuries
SUI$Complication <- sapply(SUI$DIAG, FUN=function(diag){
  compcodes <- c('T812', 'T817',
                 'T810', 'T811', 'T813', 'T814', 'T815', 'T816', 'T818', 'T819')
  maincodes <- c('S345', 'S346', 'S348', 'S357', 'S358', 'S359',
                 'S365', 'S367', 'S368', 'S369',
                 'S370', 'S371', 'S372', 'S373', 'S377', 'S378', 'S379',
                 'S390', 'S396', 'S397', 'S398', 'S399')
  suppcodes <- c('Y600', 'Y608')
  chunks <- icdsplit(diag)
  comp <- F
  if (any(compcodes %in% chunks[[1]])) {
    comp <- T
  }
  if (any(maincodes %in% chunks[[1]]) & any(suppcodes %in% chunks[[1]])) {
    comp <- T
  }
  return(comp)
})
cat(sum(SUI$Complication), "episodes admitted for previous procedural complication\n")

# complications of devices, implants and grafts
SUI$Device <- sapply(SUI$DIAG, FUN=function(diag){
  compcodes <- c('T831', 'T834', 'T835', 'T836', 'T837', 'T838', 'T839')
  suppcodes <- c('Y732', 'Y733')
```

```r
  chunks <- icdsplit(diag)
  dig <- F
  if (any(compcodes %in% chunks[[1]])) {
    dig <- T
  }
  if (any(suppcodes %in% chunks[[1]])) {
    dig <- T
  }
  return(dig)
})
cat(sum(SUI$Device), "episodes admitted for previous complication of devices\n")
cat("\n")

# episodes of admission for relevant symptoms (device complications only)
keep <- c('PSEUDO_HESID', 'SPELL', 'ADMIDATE', 'DIAG',
          'Pain', 'Bleeding', 'Complication', 'Device')
event.symptoms <- SUI[SUI$Device==T, keep]

# spells of admission for relevant symptoms
event.symptoms <- event.symptoms[order(event.symptoms$SPELL, event.symptoms$ADMIDATE),]
keep <- c("SPELL", "ADMIDATE")
dup <- duplicated(event.symptoms[,keep])
event.symptoms <- event.symptoms[!dup,]
names(event.symptoms) <- c('PSEUDO_HESID', 'SPELL', 'date.symptoms', 'DIAG',
                           'Pain', 'Bleeding', 'Complication', 'Device')
cat(nrow(event.symptoms),
    "spells with admission for relevant symptoms (device complications only)\n")

# add start times (admission date of index procedure)
keep <- c('PSEUDO_HESID', 'ADMIDATE')
event.ndx <- with(SP.SUI, SP.SUI[PSEUDO_HESID %in% event.symptoms$PSEUDO_HESID, keep])
names(event.ndx) <- c('PSEUDO_HESID', 'date.index')
event.symptoms <- merge(event.symptoms, event.ndx, by='PSEUDO_HESID')
cat(nrow(event.symptoms), "spells admitted for relevant symptoms in SUI cohort\n")

# add time and status for survival analysis
event.symptoms$time <- with(event.symptoms, as.numeric(date.symptoms-date.index))
event.symptoms$status <- 1

# remove patients admitted for pain before index
before <- which(event.symptoms$time < 0)
cat(length(before), "spells admitted for relevant symptoms before index procedure\n")
if (length(before > 0)) {
  event.symptoms <- event.symptoms[-before,]
}
cat(nrow(event.symptoms), "spells admitted for relevant symptoms after index procedure\n")

# remove spells which are index episodes
pain.at.index <- event.symptoms$SPELL %in% SP.SUI$SPELL
cat(sum(pain.at.index),
    "spells admitted for relevant symptoms which are also index episodes - removed\n")
event.symptoms <- event.symptoms[!pain.at.index,]

# remove spells which are admissions for complication within 30 days
pain.at.30 <- event.symptoms$SPELL %in% unique(TH$SPELL[TH$hasComplication])
cat(sum(pain.at.30),
    "spells admitted for relevant symptoms which are 30 day complication episodes - removed\n")
event.symptoms <- event.symptoms[!pain.at.30,]

cat(nrow(event.symptoms),
    "qualifying spells admitted for relevant symptoms for analysis\n")

# get the reason for admission for each spell
```

```r
cat("\n")

cat(sum(event.symptoms$Pain), "spells for pain;",
    length(unique(event.symptoms$PSEUDO_HESID[event.symptoms$Pain==T])),"patients\n")
cat(sum(event.symptoms$Bleeding), "spells for bleeding;",
    length(unique(event.symptoms$PSEUDO_HESID[event.symptoms$Bleeding==T])),"patients\n")
cat(sum(event.symptoms$Complication), "spells for previous complication;",
    length(unique(event.symptoms$PSEUDO_HESID[event.symptoms$Complication==T])),"patients\n")
cat(sum(event.symptoms$Device), "spells for previous device complication;",
    length(unique(event.symptoms$PSEUDO_HESID[event.symptoms$Device==T])),"patients\n")

cat("\n")

# ---------------------------------------------------
# event table for admissions with pain (in any field)
# ---------------------------------------------------
cat("Admissions with pain (in any field)\n")
cat("-----------------------------------\n")

pain.codes <- c('R100', 'R101', 'R102', 'R103', 'R104', 'R10X',
                'N930', 'N938', 'N939', 'N93X',
                'N941',
                'M726',
                'K613')

# find episodes
SUI$WithPain <- sapply(SUI$DIAG, FUN=function(diag) {
  ispain <- diag %in% pain.codes
  return(any(ispain))
})
cat(sum(SUI$WithPain), "episodes admitted with pain (", pain.codes, ")\n")

# episodes of admission with pain
keep <- c('PSEUDO_HESID', 'SPELL', 'ADMIDATE', 'DIAG')
event.withpain <- SUI[SUI$WithPain==T, keep]

# spells of admission with pain
event.withpain <- event.withpain[order(event.withpain$SPELL, event.withpain$ADMIDATE),]
keep <- c("SPELL", "ADMIDATE")
dup <- duplicated(event.withpain[,keep])
event.withpain <- event.withpain[!dup,]
names(event.withpain) <- c('PSEUDO_HESID', 'SPELL', 'date.withpain', "DIAG")
cat(nrow(event.withpain), "spells with admission with pain\n")

# change diagnosis to reason (R10, N93, N94) - use first pain code
event.withpain$reason <- sapply(event.withpain$DIAG, FUN=function(diag) {
  z <- substr(diag,1,3) %in% c('R10', 'N93', 'N94', 'M72', 'K61')
  first <- which(z)[1]
  return(substr(diag[first],1,3))
})
event.withpain$DIAG <- NULL

# add start times (admission date of index procedure)
keep <- c('PSEUDO_HESID', 'ADMIDATE')
event.ndx <- with(SP.SUI, SP.SUI[PSEUDO_HESID %in% event.withpain$PSEUDO_HESID, keep])
names(event.ndx) <- c('PSEUDO_HESID', 'date.index')
event.withpain <- merge(event.withpain, event.ndx, by='PSEUDO_HESID')
cat(nrow(event.withpain), "spells admitted with pain in SUI cohort\n")

# add time and status for survival analysis
event.withpain$time <- with(event.withpain, as.numeric(date.withpain-date.index))
event.withpain$status <- 1
```

```r
# remove patients admitted for pain before index
before <- which(event.withpain$time < 0)
cat(length(before), "spells admitted with pain before index procedure\n")
if (length(before > 0)) {
  event.withpain <- event.withpain[-before,]
}
cat(nrow(event.withpain), "spells admitted with pain after index procedure\n")

# remove spells which are index episodes
pain.at.index <- event.withpain$SPELL %in% SP.SUI$SPELL
cat(sum(pain.at.index),
    "spells admitted with pain which are also index episodes - removed\n")
event.withpain <- event.withpain[!pain.at.index,]

# remove spells which are admissions for complication within 30 days
pain.at.30 <- event.withpain$SPELL %in% unique(TH$SPELL[TH$hasComplication])
cat(sum(pain.at.30),
    "spells admitted with pain which are 30 day complication episodes - removed\n")
event.withpain <- event.withpain[!pain.at.30,]

cat(nrow(event.withpain),
    "qualifying spells admitted with pain for analysis\n")

# get the reason for admission for each spell
lv.R10 <- event.withpain$reason=='R10'
lv.N93 <- event.withpain$reason=='N93'
lv.N94 <- event.withpain$reason=='N94'
lv.M72 <- event.withpain$reason=='M72'
lv.K61 <- event.withpain$reason=='K61'
cat(sum(lv.R10), "spells with abdo+pelvic pain (R10);",
    length(unique(event.withpain$PSEUDO_HESID[lv.R10])),"patients\n")
cat(sum(lv.N93), "spells with vaginal bleeding (N93);",
    length(unique(event.withpain$PSEUDO_HESID[lv.N93])),"patients\n")
cat(sum(lv.N94), "spells with dyspareunia (N94);",
    length(unique(event.withpain$PSEUDO_HESID[lv.N94])),"patients\n")
cat(sum(lv.M72), "spells with necrotising fasciitis (M72.6);",
    length(unique(event.withpain$PSEUDO_HESID[lv.M72])),"patients\n")
cat(sum(lv.K61), "spells with ischiorectal abscess (K61.3);",
    length(unique(event.withpain$PSEUDO_HESID[lv.K61])),"patients\n")
cat("\n")

# ------------------------------------------------------
# event table for admissions including a re-intervention
# ------------------------------------------------------
cat("Admissions including re-intervention\n")
cat("------------------------------------\n")

# find all episodes with qualifying intervention
SUI$Op <- sapply(SUI$OPERTN, FUN=int.anysui)
cat(sum(SUI$Op), "episodes including SUI mesh intervention\n")

# select episodes of admission including re-intervention
keep <- c('PSEUDO_HESID', 'SPELL', 'ADMIDATE', 'OPERTN')
event.op <- SUI[SUI$Op==T, keep]

# spells of admission including re-intervention
event.op <- event.op[order(event.op$SPELL, event.op$ADMIDATE),]
keep <- c("SPELL", "ADMIDATE")
dup <- duplicated(event.op[,keep])
event.op <- event.op[!dup,]
names(event.op) <- c('PSEUDO_HESID', 'SPELL', 'date.op', "OPERTN")
cat(nrow(event.op), "spells with SUI mesh intervention\n")
```

```r
# add start times (admission date of index procedure)
keep <- c('PSEUDO_HESID', 'ADMIDATE')
event.ndx <- with(SP.SUI, SP.SUI[PSEUDO_HESID %in% event.op$PSEUDO_HESID, keep])
names(event.ndx) <- c('PSEUDO_HESID', 'date.index')
event.op <- merge(event.op, event.ndx, by='PSEUDO_HESID')
cat(nrow(event.op), "spells with SUI mesh intervention in SUI cohort\n")

# add time and status for survival analysis
event.op$time <- with(event.op, as.numeric(date.op-date.index))
event.op$status <- 1

# remove patients admitted for intervention before index (should be none!)
before <- which(event.op$time < 0)
cat(length(before), "spells with intervention for SUI mesh before index procedure\n")
if (length(before > 0)) {
  print(event.op[before,])
  event.op <- event.op[-before,]
}
cat(nrow(event.op), "spells with SUI intervention remaining\n")

# remove spells which are index episodes
op.at.index <- event.op$SPELL %in% SP.SUI$SPELL
cat(sum(op.at.index),
    "spells with SUI intervention which are also index episodes - removed\n")
event.op <- event.op[!op.at.index,]

# remove spells which are admissions for complication within 30 days
op.at.30 <- event.op$SPELL %in% unique(TH$SPELL[TH$hasComplication])
cat(sum(op.at.30),
    "spells admitted for intervention which are 30 day complication spells - removed\n")
event.op <- event.op[!op.at.30,]

cat(nrow(event.op),
    "qualifying spells admitted with SUI intervention for analysis\n")

# give reason for re-intervention (repair, re-do etc)

# (new introductions)
event.op$intro <- sapply(event.op$OPERTN, FUN=int.introduction)
cat('  ', sum(event.op$intro), "introductions\n")

# (specific repairs)
event.op$repair <- sapply(event.op$OPERTN, FUN=int.repair)
cat('  ', sum(event.op$repair), "repairs\n")

# (removals)
event.op$removal <- sapply(event.op$OPERTN, FUN=int.removal)
cat('  ', sum(event.op$removal), "removals\n")

# (renewals)
event.op$renewal <- sapply(event.op$OPERTN, FUN=int.renewal)
cat('  ', sum(event.op$renewal), "renewals\n")

cat("\n")

# -----------------
# save event tables
# -----------------
save(event.alive, event.death, event.symptoms, event.withpain, event.op,
     file="data/events.Rdata")

# --------
# clean up
```

```
# --------
rm(list=ls())
```

S5.2.11 history.R

```
# ---------------------------------------------------------------------------
# history.R
#
# Description:
# ============
# Survival and history analysis using event tables.
#
# History:
# ========
# 03.12.2015. A.J. Sims. Created.
# 07.12.2015. A.J. Sims. Report patient numbers for events in each cohort.
# 14.12.2015. A.J. Sims. Added KK's history illustration figure, and changed
#                        graphs to 5 y survival.
# 05.01.2016. A.J. Sims. Added grouping of insertions, repairs etc.
# 27.06.2016. A.J. Sims. Added distribution of admissions for all events.
# 30.06.2016. A.J. Sims. Added hazard plots.
# ---------------------------------------------------------------------------

rm(list=ls())
library("survival")
library("muhaz")
source("pubfig.R")

# ----------------
# read data frames
# ----------------
load(file="data/indexprocs.Rdata")
rm(SP.SUI)
load("data/split.Rdata")
load(file="data/events.Rdata")

# ------------------------------
# figure to illustrate histories
# ------------------------------
cat("\n")
cat("draw example history figure\n")
cat("---------------------------\n")

# patients to include
egp <- c("04016F8DDB727A30470F85C3F259A013",
         "2C9C9A9ECF90CD919362EC8CF3B6E824",
         "16483772CDE7ABF200AB3868ED90E804",
         "047DAC7480FC448A0332A1BAA40861CF")

# build data frames of times
ev <- subset(event.op, PSEUDO_HESID %in% egp, select=c(PSEUDO_HESID, time, status))
ev$status <- 4
evt <- subset(event.symptoms, PSEUDO_HESID %in% egp, select=c(PSEUDO_HESID, time, status))
evt$status <- 1
ev <- rbind(ev, evt)
evt <- subset(event.alive, PSEUDO_HESID %in% egp, select=c(PSEUDO_HESID, time, status))
evt$status <- 15
ev <- rbind(ev, evt)
evt <- subset(event.death, PSEUDO_HESID %in% egp, select=c(PSEUDO_HESID, time, status))
evt$status <- 0
ev <- rbind(ev, evt)
```

```
rm(evt)
ev <- ev[order(ev$time),]

# set up plot frame
pubfig("history")
pv <- par(no.readonly=T)
par(las=1, mar=c(5,5,0.5,0.75))
y <- 0.5
plot(NA,
    type="l",
    xlab="Time (days)",
    ylab="Example patient",
    xlim=c(0,2500),
    ylim=c(0,2.25),
    yaxt = "n")
yv <- c(0.5,1,1.5,2)
axis(2,at=yv,labels=c("4","3","2","1"))

for (p in 1:length(egp)) {
  pts <- with(ev[ev$PSEUDO_HESID==egp[p],], data.frame(x=time, y=yv[p], pch=status))
  pts <- rbind(pts, c(x=0, y=yv[p], pch=124))    # start character '|'
  with(pts, lines(x, y=y, pch=pch, type='l'))
  with(pts, points(x, y=y, pch=pch))
}

par(pv)
dev.off()
cat("see figures/history.[pdf|eps]\n")

# ----------------------------------------------------------------------------
# function subgroup
#
# Description:
# ============
# Function to do uncorrected tabulation and survival analysis on a sub-group
# of patients in the SUI cohort
#
# Arguments:
# ==========
# cohort is a vector of PSEUDO_HESIDs
# label is a string label
# ----------------------------------------------------------------------------
subgroup <- function(cohort, label) {

  event.death.ss <- event.death[event.death$PSEUDO_HESID %in% cohort,]
  event.alive.ss <- event.alive[event.alive$PSEUDO_HESID %in% cohort,]
  event.symptoms.ss <- event.symptoms[event.symptoms$PSEUDO_HESID %in% cohort,]
  event.op.ss <- event.op[event.op$PSEUDO_HESID %in% cohort,]

  cat(length(cohort), "patients in cohort\n")
  cat(nrow(event.death.ss), "died in hospital\n")
  cat(nrow(event.alive.ss), "alive at end of study\n")
  cat(nrow(event.symptoms.ss), "spells due to relevant symptoms\n")
  cat(nrow(event.op.ss), "spells with SUI mesh intervention\n")

  # total time in study
  n.fu <- nrow(event.death.ss)+nrow(event.alive.ss)
  cat(n.fu, "patients in SUI cohort with index procedure\n")
  t.fu <- sum(event.death.ss$time)+sum(event.alive.ss$time)
  cat(t.fu, "total follow-up (days),", t.fu/365.25, "years\n")
  cat(round(t.fu/n.fu, digits=1), "days mean follow-up,",
      round(t.fu/(365.25*n.fu), digits=1), "years\n")
  fu <- c(event.death.ss$time, event.alive.ss$time)
```

```
cat(round(median(fu, na.rm=T), digits=1), "days median follow-up,",
    round(median(fu, na.rm=T)/365.25, digits=1), "years\n")
for (y in 1:8) {
  cat('  ', round(100.0*sum(fu>(y*365.25))/n.fu,2),"% followed up for >", y, "year(s)\n" )
}

# admissions due to relevant symptoms
# -----------------------------------
cat("\n")
cat("Relevant symptom events:\n")
keep <- c('PSEUDO_HESID', 'time', 'status')
symptoms.all <- rbind(
  event.death.ss[, keep],
  event.alive.ss[, keep],
  event.symptoms.ss[, keep]
)
symptoms.all <- symptoms.all[order(symptoms.all$PSEUDO_HESID, symptoms.all$time),]
cat('  ', nrow(symptoms.all), "total events\n")
cat('  ', length(unique(symptoms.all$PSEUDO_HESID)), "unique patients\n")
cat('  ', sum(symptoms.all$status), "admissions for symptom events\n")
cat('  ', round(sum(symptoms.all$status)/(t.fu/365250.0),2), "per 1000 person years crude IR\n")
cat('  ', length(unique(symptoms.all$PSEUDO_HESID[symptoms.all$status==1])),
    "unique patients\n")
ct <- aggregate(symptoms.all$status, by=list(symptoms.all$PSEUDO_HESID), FUN=sum)
names(ct) <- c("PSEUDO_HESID", "n")
ct <- as.data.frame(table(ct$n))
ct <- cbind(rep(c('  '), nrow(ct)), ct)
names(ct) <- c('  ', 'n', 'freq')
cat("  Distribution of admissions for relevant symptoms:\n")
print(ct, row.names=F)

# tabulate the readmissions for symptoms
cat("  Breakdown of admissions for symptoms:\n")
cat('    ', sum(event.symptoms.ss$Pain), "pain (",
    length(unique(event.symptoms.ss$PSEUDO_HESID[event.symptoms.ss$Pain])),
    "patients)\n")
cat('    ', sum(event.symptoms.ss$Bleeding), "bleeding (",
    length(unique(event.symptoms.ss$PSEUDO_HESID[event.symptoms.ss$Bleeding])),
    "patients)\n")
cat('    ', sum(event.symptoms.ss$Complication), "complication (",
    length(unique(event.symptoms.ss$PSEUDO_HESID[event.symptoms.ss$Complication])),
    "patients)\n")
cat('    ', sum(event.symptoms.ss$Device), "device (",
    length(unique(event.symptoms.ss$PSEUDO_HESID[event.symptoms.ss$Device])),
    "patients)\n")

# admissions including further intervention
# -----------------------------------------
cat("\n")
cat("Further SUI interventions:\n")
keep <- c('PSEUDO_HESID', 'time', 'status')
op.all <- rbind(
  event.death.ss[, keep],
  event.alive.ss[, keep],
  event.op.ss[, keep]
)
op.all <- op.all[order(op.all$PSEUDO_HESID, op.all$time),]
cat('  ', nrow(op.all), "total events\n")
cat('  ', length(unique(op.all$PSEUDO_HESID)), "unique patients\n")
cat('  ', sum(op.all$status), "admissions for reintervention events\n")
cat('  ', round(sum(op.all$status)/(t.fu/365250.0),2), "per 1000 person years crude IR\n")
cat('  ', length(unique(op.all$PSEUDO_HESID[op.all$status==1])),
    "unique patients\n")
```

```r
# distribution of re-interventions
# ---------------------------------
ct <- aggregate(op.all$status, by=list(op.all$PSEUDO_HESID), FUN=sum)
names(ct) <- c("PSEUDO_HESID", "n")
ct <- as.data.frame(table(ct$n))
ct <- cbind(rep(c('  '), nrow(ct)), ct)
names(ct) <- c('  ', 'n', 'freq')
cat("   Distribution of admissions for further mesh interventions:\n")
print(ct, row.names=F)

# type of re-interventions
# ------------------------
cat("  Breakdown of further mesh interventions:\n")
cat('    ', sum(event.op.ss$intro), "introductions (",
    length(unique(event.op.ss$PSEUDO_HESID[event.op.ss$intro])), "patients)\n")

# (specific repairs)
cat("    ", sum(event.op.ss$repair), "repairs (",
    length(unique(event.op.ss$PSEUDO_HESID[event.op.ss$repair])), "patients)\n")

# (removals)
cat("    ", sum(event.op.ss$removal), "removals (",
    length(unique(event.op.ss$PSEUDO_HESID[event.op.ss$removal])), "patients)\n")

# (renewals)
cat("    ", sum(event.op.ss$renewal), "renewals (",
    length(unique(event.op.ss$PSEUDO_HESID[event.op.ss$renewal])), "patients)\n")

# cross-tabulation of symptoms and re-intervention
# ------------------------------------------------
cat("\n")
cat("Cross-tabulation of symptoms and re-intervention:\n")
keep <- c('PSEUDO_HESID', 'status')
dup <- duplicated(symptoms.all[, 'PSEUDO_HESID'])
p.first <- symptoms.all[!dup, keep]
names(p.first) <- c('PSEUDO_HESID', 'Symptoms')
dup <- duplicated(op.all[, 'PSEUDO_HESID'])
o.first <- op.all[!dup, keep]
names(o.first) <- c('PSEUDO_HESID', 'Reintervention')
op.first <- merge(x=p.first, y=o.first, by="PSEUDO_HESID", all=T)
ct <- table(Symptoms=op.first$Symptoms, Reop=op.first$Reintervention)
print(ct)

# re-intervention or symptoms event survival analysis
# ---------------------------------------------------------
cat("\n")
cat("Re-intervention or symptoms event analysis:\n")

# combine reinterventions and admissions for symptoms, but only in
# unique spells (so no double counting)
keep <- c('PSEUDO_HESID', 'SPELL', 'time', 'status')
opsym.all <- rbind(
  event.symptoms.ss[,keep],
  event.op.ss[, keep]
)
dup <- duplicated(opsym.all[,c('PSEUDO_HESID', 'SPELL')])
opsym.all <- opsym.all[!dup,]

# add censored details
keep <- c('PSEUDO_HESID', 'time', 'status')
opsym.all <- rbind(
  event.death.ss[, keep],
```

```r
    event.alive.ss[, keep],
    opsym.all[,keep]
  )
  opsym.all <- opsym.all[order(opsym.all$PSEUDO_HESID, opsym.all$time),]
  cat('  ', sum(opsym.all$status), "admissions for relevant events (in unique spells)\n")
  cat('  ', length(unique(opsym.all$PSEUDO_HESID[opsym.all$status==1])), "patients\n")
  cat('  ', round(sum(opsym.all$status)/(t.fu/365250.0),2), "per 1000 person years crude IR\n")

  # tabulate
  ct <- aggregate(opsym.all$status, by=list(opsym.all$PSEUDO_HESID), FUN=sum)
  names(ct) <- c("PSEUDO_HESID", "n")
  ct <- as.data.frame(table(ct$n))
  ct <- cbind(rep(c('  '), nrow(ct)), ct)
  names(ct) <- c('  ', 'n', 'freq')
  cat("   Distribution of admissions for relevant events:\n")
  print(ct, row.names=F)

  # hazard function for interventions and symptoms
  fit <- muhaz(opsym.all$time, opsym.all$status)
  pubfig(file=paste(label,'-hazard-either', sep='', collapse=''))
  plot(fit$est.grid/365.25, fit$haz.est*(1000*365.25),
       xlab="Follow-up time (years)",
       ylab="Hazard rate (events/1000 person years)",
       type='l',
       xlim=c(0,8), ylim=c(0,40), las=1)
  dev.off()

  # first symptoms event survival analysis
  # --------------------------------------
  cat("\n")
  cat("First relevant symptoms event analysis:\n")

  # first event K-M
  keep <- c('PSEUDO_HESID')
  dup <- duplicated(symptoms.all[, keep])
  cat('  ', sum(dup), "events after first event ",
      "(e.g. censor after first admission for relevant symptoms)\n")
  symptoms.first <- symptoms.all[!dup,]
  cat('  ', nrow(symptoms.first), "first events\n")
  cat('  ', length(unique(symptoms.first$PSEUDO_HESID)), "unique patients\n")
  cat('  ', sum(symptoms.first$status), "rows with symptom event\n")
  cat('  ', nrow(symptoms.first[symptoms.first$time==0,]), "rows with time=0 -> 0.5\n")
  cat('  ', nrow(symptoms.first[symptoms.first$time==0 & symptoms.first$status==1,]),
      "events with time=0 -> 0.5\n")
  symptoms.first$time[symptoms.first$time==0] <- 0.5 # fix at 0.5 days

  # first event survival modelling
  surv.first <- with(symptoms.first, Surv(time, status))
  km <- survfit(surv.first ~ 1, data=symptoms.first)

  # 95% survival times
  ci <- quantile(km, probs=c(0.05), conf.int=T)
  cat('  ', round(ci$quantile/365.25,2), "(", round(ci$lower/365.25,2), "to",
      round(ci$upper/365.25,2), ") years, 95% survival\n")

  # 5 year survival times
  s <- summary(km, times=c(365.25*5))
  cat('  ', round(s$surv,3), "(", round(s$lower,3), "to", round(s$upper,3),
      ") 5 year survival probability\n")

  # Kaplan-Meier and fitted survival curve plot
  pubfig(file=paste(label,'-symptoms', sep='', collapse=''))
  plot(km,
```

```
     mark.time=F,
     xscale=365.25,
     yscale=100,
     ylim=c(90,100),
     xlab="Time to first admission for relevant symptoms (years)",
     ylab="Proportion symptom free (%)", las=1)

# 5y survival
abline(v=5.0*365.25, col='black',lty='dotdash')
abline(h=s$surv, col='black', lty='dotdash')
abline(h=s$lower, col='black', lty='dotdash')
abline(h=s$upper, col='black', lty='dotdash')
dev.off()

# first re-intervention event survival analysis
# ---------------------------------------------
cat("\n")
cat("First re-intervention event analysis:\n")

# first event K-M
keep <- c('PSEUDO_HESID')
dup <- duplicated(op.all[, keep])
cat('  ', sum(dup), "events after first event (e.g. censor after re-intervention)\n")
op.first <- op.all[!dup,]
cat('  ', nrow(op.first), "first events\n")
cat('  ', length(unique(op.first$PSEUDO_HESID)), "unique patients\n")
cat('  ', sum(op.first$status), "rows with re-intervention event\n")
cat('  ', nrow(op.first[op.first$time==0,]), "rows with time=0 -> 0.5\n")
cat('  ', nrow(op.first[op.first$time==0 & op.first$status==1,]), "events with time=0 -> 0.5\n")
op.first$time[op.first$time==0] <- 0.5 # fix at 0.5 days

# first event survival modelling
surv.first <- with(op.first, Surv(time, status))
km <- survfit(surv.first ~ 1, data=op.first)

# 95% survival times
ci <- quantile(km, probs=c(0.05), conf.int=T)
cat('  ', round(ci$quantile/365.25,2), "(", round(ci$lower/365.25,2), "to",
    round(ci$upper/365.25,2), ") years, 95% survival\n")

# 5 year survival times
s <- summary(km, times=c(365.25*5))
cat('  ', round(s$surv,3), "(", round(s$lower,3), "to", round(s$upper,3),
    ") 5 year survival probability\n")

# Kaplan-Meier and fitted survival curve plot
pubfig(file=paste(label,'-reop', sep='', collapse=''))
plot(km,
     mark.time=F,
     xscale=365.25,
     yscale=100,
     ylim=c(90,100),
     xlab="Time to first SUI mesh re-intervention (years)",
     ylab="Proportion without re-intervention (%)", las=1)

# 5y survival
abline(v=5.0*365.25, col='black',lty='dotdash')
abline(h=s$surv, col='black', lty='dotdash')
abline(h=s$lower, col='black', lty='dotdash')
abline(h=s$upper, col='black', lty='dotdash')
dev.off()
```

```
  # first relevant event survival analysis
  # -------------------------------------
  cat("\n")
  cat("First relevant event survival analysis:\n")

  # first event K-M
  keep <- c('PSEUDO_HESID')
  dup <- duplicated(opsym.all[, keep])
  cat('  ', sum(dup), "events after first event ",
      "(e.g. censor after first symptoms or re-intervention)\n")
  opsym.first <- opsym.all[!dup,]
  cat('  ', nrow(opsym.first), "first events\n")
  cat('  ', length(unique(opsym.first$PSEUDO_HESID)), "unique patients\n")
  cat('  ', sum(opsym.first$status), "rows with symptoms or re-intervention event\n")
  cat('  ', nrow(opsym.first[opsym.first$time==0,]), "rows with time=0 -> 0.5\n")
  cat('  ', nrow(opsym.first[opsym.first$time==0 & opsym.first$status==1,]),
      "events with time=0 -> 0.5\n")
  opsym.first$time[opsym.first$time==0] <- 0.5 # fix at 0.5 days

  # first event survival modelling
  surv.first <- with(opsym.first, Surv(time, status))
  km <- survfit(surv.first ~ 1, data=opsym.first)

  # 95% survival times
  ci <- quantile(km, probs=c(0.05), conf.int=T)
  cat('  ', round(ci$quantile/365.25,2), "(", round(ci$lower/365.25,2), "to",
      round(ci$upper/365.25,2), ") years, 95% survival\n")

  # 5 year survival times
  s <- summary(km, times=c(365.25*5))
  cat('  ', round(s$surv,3), "(", round(s$lower,3), "to", round(s$upper,3),
      ") 5 year survival probability\n")

  # Kaplan-Meier and fitted survival curve plot
  pubfig(file=paste(label,'-either', sep='', collapse=''))
  plot(km,
       mark.time=F,
       xscale=365.25,
       yscale=100,
       ylim=c(90,100),
       xlab="Time to first mesh-related re-admission (years)",
       ylab="Proportion without mesh-related re-admission (%)", las=1)

  # 5y survival
  abline(v=5.0*365.25, col='black',lty='dotdash')
  abline(h=s$surv, col='black', lty='dotdash')
  abline(h=s$lower, col='black', lty='dotdash')
  abline(h=s$upper, col='black', lty='dotdash')
  dev.off()
}

# -------------------------------------
# survival analysis unconfounded cohort
# -------------------------------------
cat("\n")
cat("Unconfounded cohort\n")
cat("-------------------\n")
cohort <- with(SP.SUI, PSEUDO_HESID[isConfounded==F, drop=T])
subgroup(cohort, "Unconfounded cohort")

# --------------------------------------------------------------
# survival analysis unconfounded TVT, TOT or MS (no combinations)
# --------------------------------------------------------------
```

```
cat("\n")
cat("Unconfounded TVT, TOT or MS\n")
cat("---------------------------\n")
cohort <- with(SP.SUI, PSEUDO_HESID[isConfounded==F & comb==F, drop=T])
subgroup(cohort, "Unconfounded TVT, TOT or MS (no combinations)")

# --------------------------------------------
# survival analysis for TVT unconfounded cohort
# --------------------------------------------
cat("\n")
cat("Unconfounded TVT cohort\n")
cat("-----------------------\n")
cohort <- with(SP.SUI, PSEUDO_HESID[isConfounded==F & TVT==T, drop=T])
subgroup(cohort, "Unconfounded TVT")

# --------------------------------------------
# survival analysis for TOT unconfounded cohort
# --------------------------------------------
cat("\n")
cat("Unconfounded TOT cohort\n")
cat("-----------------------\n")
cohort <- with(SP.SUI, PSEUDO_HESID[isConfounded==F & TOT==T, drop=T])
subgroup(cohort, "Unconfounded TOT")

# --------------------------------------------------
# survival analysis for minisling unconfounded cohort
# --------------------------------------------------
cat("\n")
cat("Unconfounded minisling cohort\n")
cat("-----------------------------\n")
cohort <- with(SP.SUI, PSEUDO_HESID[isConfounded==F & MS==T, drop=T])
subgroup(cohort, "Unconfounded minisling")

# ----------------------------------
# survival analysis confounded cohort
# ----------------------------------
cat("\n")
cat("Confounded cohort\n")
cat("-----------------\n")
cohort <- with(SP.SUI, PSEUDO_HESID[isConfounded==T, drop=T])
subgroup(cohort, "Confounded cohort")

# ------------------------------------------------------------
# survival analysis confounded TVT, TOT or MS (no combinations)
# ------------------------------------------------------------
cat("\n")
cat("Confounded TVT, TOT or MS\n")
cat("-------------------------\n")
cohort <- with(SP.SUI, PSEUDO_HESID[isConfounded==T & comb==F, drop=T])
subgroup(cohort, "Confounded TVT, TOT or MS (no combinations)")

# --------------------------------------------
# survival analysis for TVT confounded cohort
# --------------------------------------------
cat("\n")
cat("Confounded TVT cohort\n")
cat("-----------------------\n")
cohort <- with(SP.SUI, PSEUDO_HESID[isConfounded==T & TVT==T, drop=T])
subgroup(cohort, "Confounded TVT")

# --------------------------------------------
# survival analysis for TOT confounded cohort
# --------------------------------------------
```

```
cat("\n")
cat("Confounded TOT cohort\n")
cat("------------------------\n")
cohort <- with(SP.SUI, PSEUDO_HESID[isConfounded==T & TOT==T, drop=T])
subgroup(cohort, "Confounded TOT")

# --------------------------------------------------
# survival analysis for minisling confounded cohort
# --------------------------------------------------
cat("\n")
cat("Confounded minisling cohort\n")
cat("-----------------------------\n")
cohort <- with(SP.SUI, PSEUDO_HESID[isConfounded==T & MS==T, drop=T])
subgroup(cohort, "Confounded minisling")

# --------
# clean up
# --------
rm(list=ls())
```

S5.2.12 safety.R

```
# -------------------------------------------------------------------------
# safety.R
#
# Description:
# ============
# Analysis of overall safety outcomes, taking account of peri-procedural,
# 30 day and post-procedural outcomes.
#
# History:
# ========
# 21.06.2016. A.J. Sims.
# -------------------------------------------------------------------------

# clear
rm(list=ls())

# header
cat('\n')
cat('-----------------------\n')
cat('overall safety analysis\n')
cat('-----------------------\n')
cat("\n")

# load intermediate data from each phase
load(file="data/split.Rdata")
load(file='data/thirty.Rdata')
rm(TH)
load(file='data/events.Rdata')
rm(event.alive)
rm(event.death)
rm(event.withpain)

# spells with peri-procedural complications
keep <- c('SPELL', 'PSEUDO_HESID')
spells.peri <- SP.SUI[SP.SUI$hasComplication==T,keep]

# spells with 30 day complication
keep <- c('SPELL', 'PSEUDO_HESID')
spells.30 <- comp.30[,keep]
```

```
# spells with long-term complication
keep <- c('SPELL', 'PSEUDO_HESID')
spells.lt <- rbind(event.op[,keep], event.symptoms[,keep])
dup <- duplicated(spells.lt)
spells.lt <- spells.lt[!dup,]

# print summary
cat(nrow(spells.peri), "spells with peri-procedural complication\n")
cat(nrow(spells.30), "spells with admission within 30 days for complication\n")
cat(nrow(spells.lt), "unique spells with long-term admission for surgery or symptoms\n")

# check for overlaps
spells.peri.30 <- intersect(spells.peri$SPELL, spells.30$SPELL)
cat(length(spells.peri.30), "spells common to peri-procedure and 30 day admissions\n")
spells.30.lt <- intersect(spells.30$SPELL, spells.lt$SPELL)
cat(length(spells.30.lt), "spells common to 30 day admissions and long-term admission\n")
spells.peri.lt <- intersect(spells.peri$SPELL, spells.lt$SPELL)
cat(length(spells.peri.lt), "spells common to peri-procedure and long-term admission\n")
cat("\n")

# details of cohort
keep <- c('PSEUDO_HESID', 'TVT', 'TOT', 'MS', 'comb', 'isConfounded')
cohort <- SP.SUI[,keep]

# note which (if any) event types each patient has suffered
cohort$hasComplication <- cohort$PSEUDO_HESID %in% spells.peri$PSEUDO_HESID
cohort$has30 <- cohort$PSEUDO_HESID %in% spells.30$PSEUDO_HESID
#lt <- unique(intersect(event.op£PSEUDO_HESID, event.symptoms£PSEUDO_HESID))
cohort$hasLT <- cohort$PSEUDO_HESID %in% spells.lt$PSEUDO_HESID
rm(comp.30)
#rm(lt)
rm(event.op)
rm(event.symptoms)

# print summary for a cohort
crosstab <- function(ss) {
  cat('  ', nrow(ss), "patients with first-time mesh insertion\n")
  cat('  ', sum(ss$hasComplication), "with peri-procedural complication\n")
  cat('  ', sum(ss$has30), "with 30 day readmission for complication\n")
  cat('  ', sum(ss$hasLT),
      "with post-procedural admission for device or relevant surgery\n")
  ss$early <- ss$has30 | ss$hasComplication
  cat('  ', sum(ss$early), "with peri-procedural or 30 day complication,",
      round(100*sum(ss$early)/nrow(ss),2), "%\n")
  ss$any <- ss$hasLT | ss$has30 | ss$hasComplication
  cat('  ', sum(ss$any), "with at least one of these,",
      round(100*sum(ss$any)/nrow(ss),2), "%\n")
  ct <- with(ss, table(hasComplication, has30, hasLT))
  #print(ct)
  cat("\n")
}

# unconfounded
cat("Unconfounded cohort:\n")
crosstab(cohort[!cohort$isConfounded,])

cat("Unconfounded TVT:\n")
crosstab(cohort[!cohort$isConfounded & cohort$TVT,])

cat("Unconfounded TOT:\n")
crosstab(cohort[!cohort$isConfounded & cohort$TOT,])
```

```r
cat("Unconfounded MS:\n")
crosstab(cohort[!cohort$isConfounded & cohort$MS,])

cat("Confounded cohort:\n")
crosstab(cohort[cohort$isConfounded,])

cat("Confounded TVT:\n")
crosstab(cohort[cohort$isConfounded & cohort$TVT,])

cat("Confounded TOT:\n")
crosstab(cohort[cohort$isConfounded & cohort$TOT,])

cat("Confounded MS:\n")
crosstab(cohort[cohort$isConfounded & cohort$MS,])

cat("Combinations:\n")
crosstab(cohort[cohort$isConfounded & cohort$comb,])

# clear
rm(list=ls())
```

S5.3 Support functions

S5.3.1 interventions.R

```r
# ---------------------------------------------------------------------------
# interventions.R
#
# Description:
# ============
# Functions to detect code combinations for a mesh intervention. Use
# these as functions to sapply with OPERTN lists.
#
# History:
# ========
# 14.06.2016. A.J. Sims. Created.
# ---------------------------------------------------------------------------

# episodes with introductions
int.introduction <- function(codes) {
  if (epimatch(codes, e.codes=quote(M521 | M533 | M536))) {
    supp.codes <- quote(!(Y712 | Y713 | Y716 | Y717 |
                          Y264 | Y265 |
                          Y031 | Y032 | Y033 | Y034 | Y036 | Y037 | Y038 | Y039))
    if (opcs.has.supp(codes, main=c('M521', 'M533', 'M536'), supp=supp.codes, if.none=T)) {
      return(T)
    }
  }
  return(F)
}

# episodes with removals
int.removal <- function(codes) {
  if (epimatch(codes, e.codes=quote(M534 | M535 | M537))) {
    return(T)
  }
  if (epimatch(codes, e.codes=quote(M533 | M536 | M521 | M528 | M538))) {
    supp.codes <- quote(Y037 | Y264)
    if (opcs.has.supp(codes, main=c("M533", "M536", "M521", "M528", "M538"), supp=supp.codes)) {
      return(T)
    }
```

```r
  }
  return(F)
}

# episodes with repairs
int.repair <- function(codes) {
  if (epimatch(codes, e.codes=quote(M533 | M536 | M521 | M528 | M538))) {
    supp.codes <- quote(Y031 | Y033 | Y034 | Y036 | Y038 | Y039 |
                        Y265 |
                        Y712 | Y713 | Y716 | Y717)
    if (opcs.has.supp(codes, main=c("M533", "M536", "M521", "M528", "M538"),
                      supp=supp.codes)) {
      return(T)
    }
  }
  return(F)
}

# episodes with renewals
int.renewal <- function(codes) {
  if (epimatch(codes, e.codes=quote(M533 | M536 | M521 | M528 | M538))) {
    supp.codes <- quote(Y032)
    if (opcs.has.supp(codes, main=c("M533", "M536", "M521", "M528", "M538"),
                      supp=supp.codes)) {
      return(T)
    }
  }
  return(F)
}

# POP operations (by main code)
int.pop <- function(codes) {
  pop.codes <- quote(P242 | P245 | P246 | P236 | P237 | Q544 | Q545 | Q546)
  return(epimatch(codes, e.codes=pop.codes))
}

# any SUI mesh
int.anysui <- function(codes) {
  main.codes <- quote(M521 | M533 | M536 |
                      M534 | M535 | M537)
  if (epimatch(codes, e.codes=main.codes)) {
    return(T)
  }
  if (epimatch(codes, e.codes=quote(M528 | M538))) {
    supp.codes <- quote(Y031 | Y033 | Y034 | Y036 | Y037 | Y038 | Y039 |
                          Y264 | Y265 |
                          Y712 | Y713 | Y716 | Y717)
    if (opcs.has.supp(codes, main=c("M528", "M538"), supp=supp.codes)) {
      return(T)
    }
  }
  return(F)
}

# any mesh or tape
int.anymesh <- function(codes) {
  main.codes <- quote(M521 | M533 | M536 |
                      M534 | M535 | M537 |
                      P242 | P245 | P246 | P236 | P237 | Q544 | Q545 | Q546)
  if (epimatch(codes, e.codes=main.codes)) {
    return(T)
  }
  if (epimatch(codes, e.codes=quote(M528 | M538))) {
```

```
    supp.codes <- quote(Y031 | Y033 | Y034 | Y036 | Y037 | Y038 | Y039 |
                        Y264 | Y265 |
                        Y712 | Y713 | Y716 | Y717)
    if (opcs.has.supp(codes, main=c("M528", "M538"), supp=supp.codes)) {
      return(T)
    }
  }
  return(F)
}
```

S5.3.2 printcomp.R

```
# ----------------------------------------------------------------------------
# printcomp.R
#
# Description:
# ============
# Prints and summarises complications from a normalised table of complications
# (one per row), with the following fields:
#   SPELL
#   Main Code
#   CompCode
#   SuppCode
#   TOT
#   TVT
#   MS
#   comb
#   isConfounded
#
# Returns updated normalised table with each complication classified by type.
#
# History:
# ========
# 01.06.2016. A.J. Sims. Created from pericomp.R
# ----------------------------------------------------------------------------

source("R/icdSubchapters.R")

printcomp <- function(comp.norm) {

  # add ICD-10 subchapter and descriptions
  comp.norm$ICD <- with(comp.norm, ifelse(is.na(MainCode), CompCode, MainCode))
  comp.norm$subchapter <- sapply(comp.norm$ICD, FUN=icd10.subchapter)
  comp.norm$Description <- sapply(comp.norm$ICD, FUN=icd10.description)

  # complications relating to the procedure, including injuries
  comp.norm$procedure <- apply(comp.norm, 1, FUN=function(row){
    compcodes <- c('T812', 'T817',
                   'T810', 'T811', 'T813', 'T814', 'T815', 'T816', 'T818', 'T819')

    maincodes <- c('S345', 'S346', 'S348', 'S357', 'S358', 'S359',
                   'S365', 'S367', 'S368', 'S369',
                   'S370', 'S371', 'S372', 'S373', 'S377', 'S378', 'S379',
                   'S390', 'S396', 'S397', 'S398', 'S399')

    suppcodes <- c('Y600', 'Y608')
    nbv <- F
    if (any(compcodes %in% row['CompCode'])) {
      nbv <- T
    }
    if (any(maincodes %in% row['MainCode']) & any(suppcodes %in% row['SuppCode'])) {
```

```
    nbv <- T
  }
  return(nbv)
})

# complications of devices, implants and grafts
comp.norm$device <- apply(comp.norm, 1, FUN=function(row){
  compcodes <- c('T831', 'T834', 'T835', 'T836', 'T837', 'T838', 'T839',
                 'T856', 'T857', 'T858', 'T859')
  mainsections <- c('R30', 'R31', 'R32', 'R33', 'R34', 'R35', 'R36', 'R37', 'R38', 'R39')
  suppcodes <- c('Y732', 'Y733', 'Y831')
  dig <- F
  if (any(compcodes %in% row['CompCode'])) {
    dig <- T
  }
  if (any(suppcodes %in% row['SuppCode']) &
      # if main code is NOT in chapters R30-R39
      !(row['MainCode'] %inSections% mainsections)) {
    dig <- T
  }
  return(dig)
})

# complications involving urinary symptoms arising from the procedure
comp.norm$uri <- apply(comp.norm, 1, FUN=function(row){
  mainsections <- c('R30', 'R31', 'R32', 'R33', 'R34', 'R35', 'R36', 'R37', 'R38', 'R39')
  compcodes <- c('N998', 'N999')
  uri <- F
  if (row['MainCode'] %inSections% mainsections) {
    uri <- T
  }
  if (any(compcodes %in% row['CompCode'])) {
    uri <- T
  }
  return(uri)
})

# ---------------------------------------
# complications in unconfounded operations
# ---------------------------------------

cat('\n')
cat("Complications in unconfounded operations\n")
cat("----------------------------------------\n")

cat("All reported complications:\n")
ss <- comp.norm[comp.norm$isConfounded==F,]
cat('  ', sum(ss$TVT), "TVT\n")
cat('  ', sum(ss$TOT), "TOT\n")
cat('  ', sum(ss$MS), "MS\n")
cat('  ', sum(ss$comb), "comb\n")
cat("\n")

cat("Complication code (no supplementary code):\n")
ss <- comp.norm[comp.norm$isConfounded==F & is.na(comp.norm$SuppCode),]
if (nrow(ss) > 0) {
  ct <- with(ss, {aggregate(cbind(TVT, TOT, MS, comb),
                            by=list(substr(CompCode, 1, 3)), FUN=sum)})
  if (nrow(ct)>0) {
    names(ct) <- c("CompCode", "TVT", "TOT", "MS", "comb")
    print(ct, row.names=F)
  }
}
```

```
cat("\n")

cat("Complication code (with supplementary code):\n")
ss <- comp.norm[comp.norm$isConfounded==F & !is.na(comp.norm$SuppCode),]
if (nrow(ss) > 0) {
  ct <- with(ss, {aggregate(cbind(TVT, TOT, MS, comb),
                            by=list(substr(CompCode, 1, 3)), FUN=sum)})
  if (nrow(ct) > 0) {
    names(ct) <- c("CompCode", "TVT", "TOT", "MS", "comb")
    print(ct, row.names=F)
  }
}
cat("\n")

cat("By main diagnosis code (with supplementary code):\n")
ss <- comp.norm[comp.norm$isConfounded==F,]
if (nrow(ss) > 0) {
  ct <- with(ss, {
    aggregate(cbind(TVT, TOT, MS, comb),
              by=list(substr(MainCode, 1, 3)), FUN=sum)
  })
  if (nrow(ct) > 0) {
    names(ct) <- c("MainCode", "TVT", "TOT", "MS", "comb")
    print(ct, row.names=F)
  }
}
cat("\n")

cat("By supplementary code:\n")
ss <- comp.norm[comp.norm$isConfounded==F,]
if (nrow(ss) > 0) {
  ct <- with(ss, {
    aggregate(cbind(TVT, TOT, MS, comb),
              by=list(substr(SuppCode, 1, 3)), FUN=sum)
  })
  if (nrow(ct) > 0) {
    names(ct) <- c("SuppCode", "TVT", "TOT", "MS", "comb")
    print(ct, row.names=F)
  }
}
cat("\n")

cat("By type of complication:\n")
ss <- comp.norm[comp.norm$isConfounded==F,]
nsp <- length(unique(ss$SPELL[ss$procedure==T]))
cat('  ', nsp, "spells with procedure complication (", sum(ss$procedure),
    "complications)\n")
cat('     ', length(unique(ss$SPELL[ss$procedure==T & ss$TVT==T])),
    'TVT\n')
cat('     ', length(unique(ss$SPELL[ss$procedure==T & ss$TOT==T])),
    'TOT\n')
cat('     ', length(unique(ss$SPELL[ss$procedure==T & ss$MS==T])),
    'MS\n')
cat('     ', length(unique(ss$SPELL[ss$procedure==T & ss$comb==T])),
    'combinations\n')
nsp <- length(unique(ss$SPELL[ss$device==T]))
cat('  ', nsp, "spells with device complication (", sum(ss$device),
    "complications)\n")
cat('     ', length(unique(ss$SPELL[ss$device==T & ss$TVT==T])),
    'TVT\n')
cat('     ', length(unique(ss$SPELL[ss$device==T & ss$TOT==T])),
    'TOT\n')
cat('     ', length(unique(ss$SPELL[ss$device==T & ss$MS==T])),
```

```
    'MS\n')
cat('     ', length(unique(ss$SPELL[ss$device==T & ss$comb==T])),
    'combinations\n')
nsp <- length(unique(ss$SPELL[ss$uri==T]))
cat('  ', nsp, "spells with complication with urinary symptoms (", sum(ss$uri),
    "complications)\n")
cat('     ', length(unique(ss$SPELL[ss$uri==T & ss$TVT==T])),
    'TVT\n')
cat('     ', length(unique(ss$SPELL[ss$uri==T & ss$TOT==T])),
    'TOT\n')
cat('     ', length(unique(ss$SPELL[ss$uri==T & ss$MS==T])),
    'MS\n')
cat('     ', length(unique(ss$SPELL[ss$uri==T & ss$comb==T])),
    'combinations\n')
nsp <- length(unique(ss$SPELL[ss$procedure==F & ss$device==F & ss$uri==F]))
cat('  ', nsp, "spells with other complication\n")
cat('     ',
    length(unique(ss$SPELL[ss$procedure==F & ss$device==F & ss$uri==F & ss$TVT==T])),
    'TVT\n')
cat('     ',
    length(unique(ss$SPELL[ss$procedure==F & ss$device==F & ss$uri==F & ss$TOT==T])),
    'TOT\n')
cat('     ',
    length(unique(ss$SPELL[ss$procedure==F & ss$device==F & ss$uri==F & ss$MS==T])),
    'MS\n')
cat('     ',
    length(unique(ss$SPELL[ss$procedure==F & ss$device==F & ss$uri==F & ss$comb==T])),
    'combinations\n')
cat('\n')

cat("Most common complications:\n")

cat("  Complication code (with or without supplementary code):\n")
ss <- comp.norm[comp.norm$isConfounded==F,]
ct <- with(ss, {aggregate(cbind(TVT, TOT, MS),
                          by=list(substr(CompCode, 1, 3)), FUN=sum)})
if (nrow(ct)>0) {
  names(ct) <- c("CompCode", "TVT", "TOT", "MS")
}
ct$sum <- rowSums(ct[,c("TVT", "TOT", "MS")])
ct <- ct[order(ct$sum, decreasing=T),]
print(ct, row.names=F)
cat("\n")

cat("  Main code (with supplementary code):\n")
ss <- comp.norm[comp.norm$isConfounded==F,]
ct <- with(ss, {aggregate(cbind(TVT, TOT, MS),
                          by=list(substr(MainCode, 1, 3)), FUN=sum)})
if (nrow(ct)>0) {
  names(ct) <- c("MainCode", "TVT", "TOT", "MS")
}
ct$sum <- rowSums(ct[,c("TVT", "TOT", "MS")])
ct <- ct[order(ct$sum, decreasing=T),]
print(ct, row.names=F)
cat("\n")

cat("  Complication code (with or without supplementary code) in 'other' group:\n")
ss <- comp.norm[comp.norm$isConfounded==F & comp.norm$procedure==F &
                comp.norm$device==F & comp.norm$uri==F,]
ct <- with(ss, {aggregate(cbind(TVT, TOT, MS),
                          by=list(substr(CompCode, 1, 3)), FUN=sum)})
if (nrow(ct)>0) {
  names(ct) <- c("CompCode", "TVT", "TOT", "MS")
```

```
}
ct$sum <- rowSums(ct[,c("TVT", "TOT", "MS")])
ct <- ct[order(ct$sum, decreasing=T),]
print(ct, row.names=F)
cat("\n")

cat("  Main code (with supplementary code) in 'other' group:\n")
ss <- comp.norm[comp.norm$isConfounded==F & comp.norm$procedure==F &
                 comp.norm$device==F & comp.norm$uri==F,]
ct <- with(ss, {aggregate(cbind(TVT, TOT, MS),
                          by=list(substr(MainCode, 1, 3)), FUN=sum)})
if (nrow(ct)>0) {
  names(ct) <- c("MainCode", "TVT", "TOT", "MS")
}
ct$sum <- rowSums(ct[,c("TVT", "TOT", "MS")])
ct <- ct[order(ct$sum, decreasing=T),]
print(ct, row.names=F)
cat("\n")


# --------------------------------------
# complications in confounded operations
# --------------------------------------

cat("Complications in confounded operations\n")
cat("--------------------------------------\n")

cat("All reported complications:\n")
ss <- comp.norm[comp.norm$isConfounded==T,]
cat('  ', sum(ss$TVT), "TVT\n")
cat('  ', sum(ss$TOT), "TOT\n")
cat('  ', sum(ss$MS), "MS\n")
cat('  ', sum(ss$comb), "comb\n")
cat("\n")

cat("Complication code (no supplementary code):\n")
ss <- comp.norm[comp.norm$isConfounded==T & is.na(comp.norm$SuppCode),]
if (nrow(ss) > 0) {
  ct <- with(ss, {aggregate(cbind(TVT, TOT, MS, comb),
                            by=list(substr(CompCode, 1, 3)), FUN=sum)})
  if (nrow(ct)>0) {
    names(ct) <- c("CompCode", "TVT", "TOT", "MS", "comb")
    print(ct, row.names=F)
  }
}
cat("\n")

cat("Complication code (with supplementary code):\n")
ss <- comp.norm[comp.norm$isConfounded==T & !is.na(comp.norm$SuppCode),]
if (nrow(ss) > 0) {
  ct <- with(ss, {aggregate(cbind(TVT, TOT, MS, comb),
                            by=list(substr(CompCode, 1, 3)), FUN=sum)})
  if (nrow(ct) > 0) {
    names(ct) <- c("CompCode", "TVT", "TOT", "MS", "comb")
    print(ct, row.names=F)
  }
}
cat("\n")

cat("By main diagnosis code (with supplementary code):\n")
ss <- comp.norm[comp.norm$isConfounded==T,]
if (nrow(ss) > 0) {
  ct <- with(ss, {
```

```
    aggregate(cbind(TVT, TOT, MS, comb),
              by=list(substr(MainCode, 1, 3)), FUN=sum)
  })
  if (nrow(ct) > 0) {
    names(ct) <- c("MainCode", "TVT", "TOT", "MS", "comb")
    print(ct, row.names=F)
  }
}
cat("\n")

cat("By supplementary code:\n")
ss <- comp.norm[comp.norm$isConfounded==T,]
if (nrow(ss) > 0) {
  ct <- with(ss, {
    aggregate(cbind(TVT, TOT, MS, comb), by=list(substr(SuppCode, 1, 3)), FUN=sum)
  })
  if (nrow(ct) > 0) {
    names(ct) <- c("SuppCode", "TVT", "TOT", "MS", "comb")
    print(ct, row.names=F)
 }
}
cat("\n")

cat("By type of complication:\n")
ss <- comp.norm[comp.norm$isConfounded==T,]
nsp <- length(unique(ss$SPELL[ss$procedure==T]))
cat('  ', nsp, "spells with procedure complication (", sum(ss$procedure),
    "complications)\n")
cat('     ', length(unique(ss$SPELL[ss$procedure==T & ss$TVT==T])),
    'TVT\n')

cat('     ', length(unique(ss$SPELL[ss$procedure==T & ss$TOT==T])), 'TOT\n')
cat('     ', length(unique(ss$SPELL[ss$procedure==T & ss$MS==T])),
    'MS\n')
cat('     ', length(unique(ss$SPELL[ss$procedure==T & ss$comb==T])),
    'combinations\n')
nsp <- length(unique(ss$SPELL[ss$device==T]))
cat('  ', nsp, "spells with device complication (", sum(ss$device),
    "complications)\n")
cat('     ', length(unique(ss$SPELL[ss$device==T & ss$TVT==T])),
    'TVT\n')
cat('     ', length(unique(ss$SPELL[ss$device==T & ss$TOT==T])),
    'TOT\n')
cat('     ', length(unique(ss$SPELL[ss$device==T & ss$MS==T])),
    'MS\n')
cat('     ', length(unique(ss$SPELL[ss$device==T & ss$comb==T])),
    'combinations\n')
nsp <- length(unique(ss$SPELL[ss$uri==T]))
cat('  ', nsp, "spells with complication with urinary symptoms (", sum(ss$uri),
    "complications)\n")
cat('     ', length(unique(ss$SPELL[ss$uri==T & ss$TVT==T])),
    'TVT\n')
cat('     ', length(unique(ss$SPELL[ss$uri==T & ss$TOT==T])),
    'TOT\n')
cat('     ', length(unique(ss$SPELL[ss$uri==T & ss$MS==T])),
    'MS\n')
cat('     ', length(unique(ss$SPELL[ss$uri==T & ss$comb==T])),
    'combinations\n')
nsp <- length(unique(ss$SPELL[ss$procedure==F & ss$device==F & ss$uri==F]))
cat('  ', nsp, "spells with other complication\n")
cat('     ',
    length(unique(ss$SPELL[ss$procedure==F & ss$device==F & ss$uri==F & ss$TVT==T])),
    'TVT\n')
```

```
  cat('     ',
      length(unique(ss$SPELL[ss$procedure==F & ss$device==F & ss$uri==F & ss$TOT==T])),
      'TOT\n')
  cat('     ',
      length(unique(ss$SPELL[ss$procedure==F & ss$device==F & ss$uri==F & ss$MS==T])),
      'MS\n')
  cat('     ',
      length(unique(ss$SPELL[ss$procedure==F & ss$device==F & ss$uri==F & ss$comb==T])),
      'combinations\n')
  cat('\n')

  cat("Most common complications:\n")

  cat("  Complication code (with or without supplementary code):\n")
  ss <- comp.norm[comp.norm$isConfounded==T,]
  ct <- with(ss, {aggregate(cbind(TVT, TOT, MS), by=list(substr(CompCode, 1, 3)), FUN=sum)})
  if (nrow(ct)>0) {
    names(ct) <- c("CompCode", "TVT", "TOT", "MS")
  }
  ct$sum <- rowSums(ct[,c("TVT", "TOT", "MS")])
  ct <- ct[order(ct$sum, decreasing=T),]
  print(ct, row.names=F)
  cat("\n")

  cat("  Main code (with supplementary code):\n")
  ss <- comp.norm[comp.norm$isConfounded==T,]
  ct <- with(ss, {aggregate(cbind(TVT, TOT, MS), by=list(substr(MainCode, 1, 3)), FUN=sum)})
  if (nrow(ct)>0) {
    names(ct) <- c("MainCode", "TVT", "TOT", "MS")
  }
  ct$sum <- rowSums(ct[,c("TVT", "TOT", "MS")])
  ct <- ct[order(ct$sum, decreasing=T),]
  print(ct, row.names=F)
  cat("\n")

  # return updated normalised complication table
  return(comp.norm)
}
```

S5.3.3 pubfig.R

```
pubfig <- function(file) {

  # -----------------------------------------------------------------------
  # Description:
  # ------------
  # opens graphics output to create publication quality figure. Edit this
  # file to set appropriate options according to journal requirements.
  # Places figures in /figure folder.
  #
  # History:
  # --------
  # 21.02.2017. A.J. Sims.  Created.
  # -----------------------------------------------------------------------

  # PLOS ONE pdf (for Supporting figures)
  pdf(file=paste('figure/', file, '.pdf', sep=''), width=5.2, height=5.2,
      pointsize=12*5.2/7.0)

  # PLOS ONE encapsulated postscript
  #postscript(file=paste('figure/', file, '.eps', sep=''),
```

```
#           onefile=F, horizontal=F, paper="special",
#           width=5.2, height=5.2, pointsize=12*5.2/7.0)

}
```

S5.3.4 epimatch.R

```
epimatch <- function(r, e.codes) {

  # ----------------------------------------------------------------------------
  # Description:
  # ============
  # Matching function for HES episodes. Use in conjunction with sapply function
  # on lists of lists. Arguments as follows:
  #   r      : a list of procedure or diagnostic codes
  #   e.codes: a boolean combination of (4 character) procedure codes, as an
  #            expression. For example quote((P236 & P237) | P231) will
  #            match episodes that include both P236 and P237, or P231.
  #            quote(P236 & !P237) will match episodes that include P236 excluding
  #            those that also contain P237.
  #
  # The technique works as follows. Argument e.codes is treated as an R
  # expression. When it parses the expression, R treats the codes (P123 etc)
  # as variables. Function 'all.vars' returns the (unique) variables in the
  # expression. This function then replaces (using substitute) each
  # variable (i.e. code) with an expression that searches for that particular
  # code in the list of codes. Because of the way that the expression allows
  # different searches to be combined, each code must be searched separately.
  #
  # History:
  # ========
  # 07.05.2014. A.J. Sims. Created.
  # 08.05.2014. K.K.       Modified have already restricted to procedure fields in
  #                        main code, no need to repeat, substituted 'sub' with
  #                        'gsub' function
  # 14.05.2014. A.J. Sims. Change above means that all fields in 'row' will be
  #                        searched. If this is to be restricted to operation fields
  #                        then only those columns of a data frame should be sent to
  #                        the apply function. This function will also work for
  #                        diagnostic codes if only those fields are provided. The
  #                        argument name (e.codes) has been changed to reflect that.
  # 14.05.2014. A.J. Sims. The %in% operator, used to search for the occurrence of
  #                        a (single) particular code in the list of all codes
  #                        supplied can be replaced by 'pmatch' if allow.partial
  #                        is set TRUE. This allows partial matching, e.g. 'P23' will
  #                        match 'P231', 'P232' etc.
  # 18.08.2015. A.J.Sims.  Replaced use of parse() with direct substitution into
  #                        an expression. e.codes should be passed in as an
  #                        expression, e.g. quote((P236 & P237) | P231) and
  #                        argument allow.partial has been removed. Improves
  #                        performance and safety. Note recursive use of
  #                        substitute() to allow lexical editing of an
  #                        expression stored in a variable rather than a literal.
  # ----------------------------------------------------------------------------

  # ----------------------------------------------------------------------------
  # construct and apply a boolean match expression for all procedures...
  # ----------------------------------------------------------------------------

  # build a paired list of variables to subtitute (one for each in the
  # call expression)
```

```
  sublist <- list()

  # template expression for each variable in e.codes (X is substituted with
  # each of the variables in the calling logical expression)
  template <- quote((c(X) %in% r))

  # focus on only the variables from the text expression and search for each in array
  for (v in all.vars(e.codes)) {
    rep <- eval(substitute(substitute(e, list(X=v)), list(e=template)))
    sublist[[v]] <- rep
  }
  expr <- eval(substitute(substitute(e, env=sublist), env=list(e=e.codes)))

  return(eval(expr))
}
```

S5.3.5 icdsplit.R

```
icdsplit <- function(codes) {

  # --------------------------------------------------------------------------
  # Description:
  # ============
  # Separates a list of ICD-10 codes into a sequence (list) of lists,
  # with each element of the sequence starting with a main code.
  # Needed due to the presence of supplementary codes in Chapter XX
  # of ICD-10 (range V01 to Y98). Works by detecting main codes
  # (as logical NOT of supplementary codes), uses cumsum
  # to generate a sequence of chunk labels the same length as the
  # code sequence, e.g. 1123334556 etc, then split.
  #
  # History:
  # ========
  # 11.08.2015. A.J. Sims. Created.
  #
  # Arguments:
  # ==========
  # 'codes' is a list of ICD-10 codes in HES order; it consists of
  # a sequence of main codes, each followed by zero or more
  # supplementary codes.
  #
  # Value:
  # ======
  # A list of lists of ICD-10 code sequences, with each low level list
  # starting with a main code and followed by supplementary codes. Many
  # of the low level lists will have only one entry.
  # --------------------------------------------------------------------------

  # find indices of supplementary codes (anything in chapters V, W, X, Y)
  table <- c("V", "W", "X", "Y")
  mask <- cumsum(!(substr(codes,1,1) %in% table))   # 11122345567 etc
  return(split(codes,mask))
}
```

S5.3.6 icdSubchapters.R

```
# ----------------------------------------------------------------------------
# icdSubchapters.R
#
```

```
# Description:
# ============
# Reads subchapter names into a data frame, and offers a lookup
# function to search them with a given code.
#
# History:
# ========
# 11.12.2015. A.J. Sims. Created from Sam Urwin's file.
# --------------------------------------------------------------------------

# read sub-chapter names into a data frame (will be executed when R file
# is loaded with "source")

ICD10.subchapters <- read.csv2(header=T, stringsAsFactors=F, text=[14719 chars quoted with '"'])
ICD10.subchapters <- ICD10.subchapters[order(ICD10.subchapters$Start),]

# function to return sub chapter name, based on code
icd10.subchapter <- function(code) {
  if (is.na(code)) {return(NA)}
  code <- gsub("X$", '', code)      # remove
  lvs <- (ICD10.subchapters$Start <= code)
  lve <- (ICD10.subchapters$End >= code)
  index <- intersect(which(lvs), which(lve))
  return(paste(ICD10.subchapters$Start[index],"-",ICD10.subchapters$End[index]))
}

icd10.description <- function(code) {
  if (is.na(code)) {return(NA)}
  code <- gsub("X$", '', code)      # remove
  lvs <- (ICD10.subchapters$Start <= code)
  lve <- (ICD10.subchapters$End >= code)
  index <- intersect(which(lvs), which(lve))
  return(ICD10.subchapters$Description[index])
}
```

S5.3.7 inChapters.R

```
# --------------------------------------------------------------------------
# inChapters.R
#
# Description:
# ============
# Function to test which of a vector of (3 digit) codes match
# to a list of chapters.
#
# History:
# ========
# 01.05.2015. A.J. Sims. Created.
# 19.08.2015. A.J. Sims. Now returns logical vector (like %in%).
# --------------------------------------------------------------------------

#' @title Chapter match
#'
#' Match OPCS-4 or ICD-10 3 letter codes to chapters
#'
#' @param codes vector of 3-character codes to be matched
#' @param table vector of chapters to be matched against.
#'
#' @return A logical vector of the same length as x.
#'
#' @export
```

```r
"%inChapters%" <- function(codes, table) {

  # convert codes to single (first) letter
  mat <- match(substr(codes,1,1), table, nomatch=0)
  return(mat != 0)
}
```

S5.3.8 inSections.R

```r
# ---------------------------------------------------------------------------
# inSections.R
#
# Description:
# ============
# Function to test which of a vector of (3 digit) codes match
# to a list of sections, e.g. (A01, P23).
#
# History:
# ========
# 01.05.2015. A.J. Sims. Created.
# 19.08.2015. A.J. Sims. Now returns logical vector (like %in%).
# ---------------------------------------------------------------------------

#' @title OPCS-4/ICD-10 section match
#'
#' Match OPCS-4 or ICD-10 3 letter codes to sections
#'
#' @param codes vector of 3-character codes to be matched
#' @param table vector of sections to be matched against.
#'
#' @return A logical vector of the same length as x.
#'
#' @export
"%inSections%" <- function(codes, table) {

  # convert codes to first 3 characters
  mat <-match(substr(codes,1,3), table, nomatch=0)
  return(mat != 0)
}
```

S5.3.9 is_complication.R

```r
# --------------------------------------------------------------------------
# is_complication.R
#
# Description:
# ============
# Identify episodes with in-episode complications from any code sequence
# where the code sequence (argument 'chunk') is a list of codes starting
# with a main ICD-10 code and followed by zero or more supplementary codes.
#
# Returns a list of:
#  complication code (if applicable)
#  main code         (if followed by a Y code)
#  supp code         the first supplementary qualifier code.
#
#
# Arguments:
# ==========
```

```r
# chunk:        a list of ICD-10 codes following chunk rules (i.e. starts
#               with a code from chapter A-U or Z, followed by zero or more
#               supplementary codes from chapters V, W, X, Y).
#
# History:
# ========
# 18.08.2015. A.J. Sims. Created from Sam Urwin & Kim Keltie's original
#                        based on individual codes.
# 11.09.2015. A.J. Sims. Returns main diagosis codes, complication code
#                        (in main field) and supplementary code.
# 27.05.2016. A.J. Sims. Defined post-procedure codes now listed as post.codes.
# ---------------------------------------------------------------------------------

is.complication <- function(chunk) {

  # List of T80-T88 complication codes...
  T.sections <- c(# Complications following infusion, transfusion and therapetuic injection
    'T80',
    # Complication of procedures NEC
    'T81',
    # Complications of cardiac & vascular prosthetics
    'T82',
    # Complications of genitourinary prosthetic devices, implants and grafts
    'T83',
    # Complications of internal orthopaedic prosthetic devices, implants and grafts
    'T84',
    # Complications of other internal prosthetic devices, implants and grafts
    'T85',
    # Failure and rejection of transplanted organs and tissues
    'T86',
    # Complications peculiar to reattachment and amputation
    'T87',
    # Other complications of surgical & med care NEC
    'T88')

  # List of Y complication codes...
  Y.sections <- c(
    # Drug adverse events
    'Y40',
    'Y41',
    'Y42',
    'Y43',
    'Y44',
    'Y45',
    'Y46',
    'Y47',
    'Y48',
    'Y49',
    'Y50',
    'Y51',
    'Y52',
    'Y53',
    'Y54',
    'Y55',
    'Y56',
    'Y57',
    'Y58',
    'Y59',
    # Unintentional cut, puncture, perforation or haemorrhage during surgical and medical care
    'Y60',
    # Foreign object accidentally left in body during surgical and medical care
    'Y61',
    # Failure of sterile precautions during surgical and medical care
```

```
  'Y62',
  # Failure in dosage during surgical and medical care
  'Y63',
  # Contaminated medical or biological substances
  'Y64',
  # Other misadventures during surgical and medical care
  'Y65',
  # Nonadministration of surgical and medical care
  'Y66',
  # Unspecified misadventure during surgical and medical care
  'Y69',
  # Anaesthesiology devices associated with adverse incidents
  'Y70',
  # Cardiovascular devices associated with adverse incidents
  'Y71',
  # Otorhinolaryngological devices assocaited with adverse incidents
  'Y72',
  # Gastroenterology and uroloft devices associated with adverse incidents
  'Y73',
  # General hospital and personal-use devices associated with adverse incidents
  'Y74',
  # Neurological devices associated with adverse incidents
  'Y75',
  # Obstetric and gynaecological devices associated with adverse incidents
  'Y76',
  # Opthalmic devices associated with adverse incidents
  'Y77',
  # Radiological devices associated with adverse incidents
  'Y78',
  # Orthopaedic devices assocaited with adverse events
  'Y79',
  # Physical medicine devices associated with adverse incidents
  'Y80',
  # General and plastic surgical devices assocaited with adverse incidents
  'Y81',
  # Other and unspecified medical devices associated with adverse incidents
  'Y82',
  # Surgical operation and other surgical procedures as the cause of abnormal
  # reaction of the patient, or of later complication, without mention of
  # misadventure at the time of the procedure
  'Y83',
  # Other medical procedures as the cause of abnormal reaction of the patient,
  # or of later complication, without mention of misadventure at the time of the procedure
  'Y84')

# post-procedure complication codes (n.b. context specific)
post.codes <- c(
 'G970', 'G971', 'G978', 'G979',
 'I978', 'I979',
 'J950', 'J952', 'J953', 'J954', 'J955', 'J956', 'J957', 'J958', 'J959',
 'K914', 'K918', 'K919',
 'M968', 'M969',
 'N990', 'N991', 'N992', 'N994', 'N995', 'N996', 'N998', 'N999'
)

# set output
main <- NA
comp <- NA
supp <- NA

# 'T' or 'other' as main code
if ((chunk[1] %inSections% T.sections) | (chunk[1] %in% post.codes)) {
  comp <- chunk[1]
```

```
  }

  # 'Y' in supplementary codes
  nc <- length(chunk)
  if (nc > 1) {
    ndx <- which(chunk %inSections% Y.sections)   # no need to start at 2 because 1 is not Y.
    if (length(ndx) > 0) {
      supp <- chunk[ndx[1]]                        # the first qualifying Y code
      if (is.na(comp)) {
        main <- chunk[1]
      }
    }
  }
  return(c(MainCode=main, CompCode=comp, SuppCode=supp))
}
```

S5.3.10 opcs_has_supp.R

```
# ------------------------------------------------------------------------
# opcs_has_supp.R
#
# Description:
# ============
# Takes a list of OPCS supplementary codes and checks whether the specified
# main code is followed by a supplementary code.
#
# Arguments:
# ==========
# codes:   A list of OPCS codes in HES order
# main:    Main codes of interest (must be in 'codes') - a list.
# supp:    A quote expression of candidate supplementary codes (in
#          'epimatch' syntax, e.g. (Y123 | Y124) & Y111.
# if.none  Response to return if there are no supplementary codes following
#          the main code. Normally (and by default) FALSE, because in most
#          cases the 'supp' expression will be looking for the presence of
#          one or more supplementary codes. But if 'supp' is a list of
#          codes not wanted, eg !(Y123 | Y456), then set if.none=T because
#          the main code on its own qualifies.
#
# Value:
# ======
# Logical.
#
# History:
# ========
# 17.09.2015. A.J. Sims. Created.
# 05.12.2015. A.J. Sims. Added if.none argument and changed logic in test.
# 14.06.2016. A.J. Sims. Allowed main to be a list of matching codes.
# ------------------------------------------------------------------------------------

opcs.has.supp <- function(codes, main, supp, if.none=F) {

  # split into chunks and test each one
  chunks <- opcssplit(codes)
  has.supp <- sapply(chunks, FUN=function(chunk) {
    rc <- F
    if (chunk[1] %in% main) {
      nc <- length(chunk)
      if (nc==1) {return(if.none)}               # there are no supplementary codes
      rc <- epimatch(chunk[2:nc], e.codes=supp)
    }
```

```
    return(rc)
  })
  return(any(has.supp))
}
```

S5.3.11 opcssplit.R

```
opcssplit <- function(codes) {

  # ------------------------------------------------------------------------
  # Description:
  # ============
  # Separates a list of OPCS-4 codes into a sequence (list) of lists,
  # with each element of the sequence starting with a main code.
  # Needed due to the presence of supplementary codes in OPCS-4.
  # Works by detecting main codes
  # (as logical NOT of supplementary codes), uses cumsum
  # to generate a sequence of chunk labels the same length as the
  # code sequence, e.g. 1123334556 etc, then split.
  #
  # History:
  # ========
  # 11.08.2015. A.J. Sims. Created.
  # 12.05.2016. A.J. Sims. Added O chapter as supplementary codes.
  #
  # Arguments:
  # ==========
  # 'codes' is a list of OPCS-4 codes in HES order; it consists of
  #    a sequence of main codes, each followed by zero or more
  #    supplementary codes.
  #
  # Value:
  # ======
  # A list of lists of OPCS-4 code sequences, with each low level list
  # starting with a main code and followed by supplementary codes. Many
  # of the low level lists will have only one entry.
  # ------------------------------------------------------------------------

  # find indices of subsidiary codes (anything in chapters Y or Z)
  table <- c("O", "Y", "Z")
  # these should also match (chapter 'O' codes used as overspill)
  #  O11        Other upper digestive tract
  #  O12        Branch of external carotid artery
  #  O13        Other leg region
  #  O14        Other lymph node
  #  O16        Body region
  #  O28        Other cerebral artery
  #  O30        Other large intestine
  #  O31        Other arm region
  #  O33        Bone of skull

  mask <- cumsum(!(substr(codes,1,1) %in% table))   # 11122345567 etc
  return(split(codes,mask))
}
```

Supplementary File S6: Output from analysis

```
2016-07-14 18:03:47
R version 3.3.1 (2016-06-21)

HES extract
-----------
SQL table: 20151126_RX029_MESH_0708_1415_M533M536M521_allep
564464 rows in table 20151126_RX029_MESH_0708_1415_M533M536M521_allep

Raw data
--------
564463 episodes
101081 patients

Checks on data:
--------------
ADMIDATE range:  2005-06-22 2015-03-31
DISDATE range:  2007-04-01 2015-03-31
EPIEND range:  2007-04-01 2015-03-31
EPISTART range:  2005-10-25 2015-03-31
FYEAR values: [1] 0708 0809 0910 1011 1112 1213 1314 1415
Levels: 0708 0809 0910 1011 1112 1213 1314 1415
ADMIMETH values: [1] 28 21 11 22 24 13 32 31 12 81 99 23 82 83 98 2D 25 2A 2B 2C
Levels: 11 12 13 21 22 23 24 25 28 2A 2B 2C 2D 31 32 81 82 83 98 99
DISDEST values: [1] 19 98 51 79 52 50 85 99 53 29 49 38 87 65 88 54 84 37 48 30 66 NA
DISMETH values:[1]  1  8  2  4  9  3 NA
SEX values:[1] 2 1 0 9
STARTAGE range: 0 102
120377 bad OPERTN codes out of 564463 episodes
 code   freq
    - 120209
 -         8
    &     84
 &        76
0 bad DIAG codes out of 564463 episodes
Bad ICD-10 codes:


SUI cohort
----------
103707 episodes containing M533 | M536 | M521
101081 patients
564463 episodes for these patients

POP cohort
----------
2933 episodes containing P236 | P237 | P242 | P245 | P246 | Q544 | Q545 | Q546
2743 patients
20183 episodes for these patients

Raw data
--------
564463 episodes
101081 patients

Activity - incontinence
-----------------------
63576 episodes with M533
  8820 in 0708
  8507 in 0809
  8397 in 0910
  8096 in 1011
  8174 in 1112
  7628 in 1213
  7612 in 1314
  6342 in 1415
38997 episodes with M536
  5100 in 0708
  5752 in 0809
  5571 in 0910
```

```
  5435 in 1011
  4887 in 1112
  4485 in 1213
  4366 in 1314
  3401 in 1415
1225 episodes with M521
   211 in 0708
   152 in 0809
   142 in 0910
   130 in 1011
   135 in 1112
   136 in 1213
   179 in 1314
   140 in 1415
899 episodes with M534
    66 in 0708
   104 in 0809
   100 in 0910
   121 in 1011
   130 in 1112
   124 in 1213
   124 in 1314
   130 in 1415
1868 episodes with M535
   110 in 0708
   214 in 0809
   207 in 0910
   233 in 1011
   266 in 1112
   301 in 1213
   267 in 1314
   270 in 1415
49 episodes with M533 & (Y037 | Y264)
     3 in 0708
     4 in 0809
     8 in 0910
    16 in 1011
     8 in 1112
     3 in 1213
     3 in 1314
     4 in 1415
607 episodes with M537
    31 in 0708
    65 in 0809
    96 in 0910
    81 in 1011
    72 in 1112
    96 in 1213
    84 in 1314
    82 in 1415
24 episodes with M536 & (Y037 | Y264)
     1 in 0708
     7 in 0809
     4 in 0910
     3 in 1011
     3 in 1112
     3 in 1213
     3 in 1314
     0 in 1415

Duplicated episodes
-------------------
1410 duplicated episodes removed
563053 episodes remaining

Missing data (all episodes)
--------------------------
0 episodes with missing PSEUDO_HESID
0 episodes with missing SEX
91 episodes with missing ADMIDATE
0 episodes with missing ADMIMETH
0 episodes with missing PROCODE3
0 episodes with missing FYEAR
```

```
91 unique episodes with missing essential fields
91 patients
476 episodes for these patients to be dropped
562577 remaining episodes
101004 remaining patients

Inclusion criteria (all episodes)
---------------------------------
176 episodes with SEX != 2
176 unique episodes not matching inclusion criteria
176 patients
176 episodes for these patients to be dropped
562401 remaining episodes
100914 remaining patients

Find index episodes
-------------------
109136 episodes with at least 1 main tape/mesh code in OPERTN
100914 index episodes
0 patients with no index

Missing data (index episodes)
-----------------------------
60 index episodes with missing STARTAGE
60 unique index episodes with missing essential data
60 patients
69 episodes for these patients to be dropped
562332 remaining episodes
100854 remaining patients

Find index episodes
-------------------
109076 episodes with at least 1 main tape/mesh code in OPERTN
100854 index episodes
0 patients with no index

Inclusion criteria (index episodes)
-----------------------------------
46 index episodes with STARTAGE < 18
9 index episodes with disallowed ADMIMETH
55 unique index episodes not matching inclusion criteria
55 patients
656 episodes for these patients to be dropped
561676 remaining episodes
100799 remaining patients

Patients with episodes AFTER date of death
------------------------------------------
923 in-hospital death episodes
864 in-hospital death spells
4 patients with episodes AFTER date of death
  SPELL   ADMIDATE    DISDATE
  64212 2008-05-11 2008-05-11
 209551 2010-06-10 2010-07-03
 227932 2013-02-05 2013-02-05
 416586 2012-05-10 2012-05-11
     NA       <NA>       <NA>
5 patients
45 episodes for these patients to be dropped
561631 remaining episodes
100795 remaining patients

Find index episodes
-------------------
109016 episodes with at least 1 main tape/mesh code in OPERTN
100795 index episodes
0 patients with no index

Bad OPCS and ICD-10 codes
-------------------------
0 bad OPERTN codes out of 561631 episodes
0 bad DIAG codes out of 561631 episodes
```

```
SUI index spells
----------------
101364 episodes in index spells
100795 index episodes
569 non-index episodes

Information about index spells
------------------------------
100795 index spells
446 index spells with >1 episode
93911 index spells with SUI diagnosis
6884 index spells without SUI diagnosis
100795 index spells with demographic details found
1 spells with LoS < 0 days; LoS set to NA
93911 spells with SUI diagnosis and demographic details

Classify index spells by type of mesh operation
-----------------------------------------------
391 index episodes with removal of mesh
284 index episodes with repair of mesh
10 index episodes with renewal of mesh
2139 index episodes involving POP surgery
2810 index episodes with mesh removal/repair/renewal or pop surgery
1665 'index' spells with repair/removal/renewal or POP surgery - excluded
92246 remaining index spells (92246 patients)

37796 index episodes including TOT main code
61017 index episodes including TVT main code
995 index episodes including MS main code
Check for combinations of TVT, TOT, MS:
   0 spells with no TVT, TOT or MS
   92186 spells with one of TVT, TOT or MS
   60 spells with more than one TVT, TOT or MS
   34704 index spells with TOT only
   56648 index spells with TVT only
   834 index spells with MS only
   60 index spells with combination

92724 episodes in eligible spells marked

Identify spells with complication
---------------------------------
92724 index episodes in eligible spells
2906 index episodes in eligible spells with complication
2866 eligible index spells with complication
spells with complication written in code normalised order to spells.comp:
   20020 code chunks
   2866 unique spells
   2947 unique episodes

Index spells with 'late' operations
-----------------------------------
62 index spells with late operation code (Y712, Y713, Y716, Y717)

Generating activity graph
-------------------------
Activity graph written to figure/activity.pdf

Index spells with cystoscopy
----------------------------
22736 index spells with endoscopy of bladder
31 index spells with endoscopy of urethra
22747 index spells with endoscopy of either bladder or urethra
2494 index spells with catheterization

Split cohort by presence of confounding procedure
-------------------------------------------------
2947 episodes in bad spells
92246 eligible index spells
   68045 index spells without confounding operation
   24201 index spells with confounding operation

Unconfounded spells with combination treated as confounded
```

```
   68002 index spells without confounding operation
   24244 index spells with confounding operation

24230 eligible index spell episodes with confounding operation
68494 eligible index spell episodes without confounding operation

Confounding procedures in index spells (by OPCS-4 section)
----------------------------------------------------------
(Potential rescue procedures included in this list)
 OPCS section Frequency
          A48         1
          A70         1
          A73        30
          A79         1
          B28         5
          B34         2
          B35         1
          C17         1
          C71         1
          D15         1
          E04         1
          E33         1
          E36         2
          E37         1
          E42         4
          E48         1
          E59         1
          F09         1
          F10         1
          F17         1
          G28         1
          G43         1
          G45         9
          G47         1
          G69         6
          G74         5
          G78        12
          H02         2
          H03         1
          H04         1
          H07         1
          H10         1
          H11         1
          H15         8
          H19         4
          H20         2
          H22         1
          H24         1
          H25         4
          H28         1
          H29         1
          H30         1
          H33        16
          H35       103
          H36        87
          H41        18
          H42        44
          H44         1
          H46         9
          H48        42
          H50        26
          H51        12
          H52         6
          H55         3
          H56        12
          H57         3
          H58         1
          H62         4
          H69         1
          J18         8
          J20         1
          J45         4
          K63         5
```

K66	1
K75	2
L13	2
L68	6
L70	9
L71	7
L91	2
L93	4
L94	1
L97	5
M02	1
M06	2
M10	1
M13	1
M15	1
M16	1
M17	2
M18	1
M19	11
M20	1
M22	2
M25	18
M27	10
M29	25
M32	7
M33	3
M34	3
M35	1
M36	17
M37	138
M38	589
M39	2
M41	5
M42	34
M43	316
M44	61
M48	11
M49	82
M51	13
M52	86
M53	663
M54	40
M55	4
M56	32
M58	10
M64	1
M72	42
M73	65
M75	4
M76	195
M79	160
M81	44
O14	1
O23	3
O26	2
O30	2
P01	3
P03	52
P05	392
P06	40
P07	18
P09	231
P11	53
P13	1744
P14	14
P15	23
P17	9
P18	119
P19	13
P20	296
P21	37
P22	160
P23	22044

P24	2433
P25	521
P26	144
P27	30
P29	206
P31	57
P32	13
Q01	198
Q02	117
Q03	25
Q05	16
Q07	545
Q08	4923
Q09	13
Q10	105
Q11	2
Q12	2338
Q15	14
Q16	405
Q17	480
Q20	76
Q22	671
Q23	221
Q24	14
Q25	5
Q27	11
Q28	1
Q31	6
Q32	3
Q35	400
Q36	3
Q37	4
Q38	6
Q41	8
Q43	39
Q44	2
Q45	5
Q47	19
Q49	90
Q51	3
Q52	8
Q54	250
S02	18
S03	2
S04	1
S06	109
S08	1
S11	4
S13	27
S15	6
S17	1
S23	3
S24	2
S25	2
S27	2
S31	1
S36	1
S39	20
S40	1
S41	2
S42	9
S43	2
S47	9
S50	1
S52	55
S53	2
S57	9
S60	52
S62	18
S70	1
T12	2
T20	13
T21	2

T24	14
T25	12
T26	1
T27	6
T28	6
T29	4
T30	61
T31	4
T33	3
T34	12
T36	16
T41	114
T42	206
T43	236
T46	22
T48	4
T50	2
T57	32
T87	3
T96	3
T97	1
W06	1
W20	1
W24	1
W28	1
W33	1
W47	1
X33	9
X34	4
X35	2
X37	1
X38	5
X40	3
X41	1
X49	1
X50	5
X52	1
X70	6
X83	13
X85	216
X87	1
X90	2
X92	2
X95	1
X96	1
Y03	199
Y05	10
Y06	33
Y07	8
Y08	4
Y10	1
Y11	108
Y12	1
Y13	50
Y14	2
Y15	2
Y16	1
Y17	1
Y18	35
Y20	125
Y26	65
Y27	55
Y30	3
Y36	21
Y37	4
Y38	12
Y39	1
Y40	5
Y44	5
Y51	1
Y56	2
Y59	1
Y60	81

Y61	3
Y62	1
Y63	1
Y67	5
Y69	8
Y71	302
Y78	1
Y95	2
Z02	1
Z06	1
Z11	78
Z15	1
Z18	1
Z24	2
Z27	9
Z28	5
Z29	9
Z37	5
Z38	1
Z39	1
Z40	50
Z45	44
Z46	13
Z47	1
Z48	2
Z49	75
Z50	41
Z57	2
Z58	2
Z60	3
Z62	1
Z66	35
Z67	1
Z72	1
Z75	3
Z76	1
Z85	1
Z86	1
Z87	1
Z89	1
Z90	7
Z91	3
Z92	115
Z93	5
Z94	1053
Z95	4
Z96	11
Z98	2
Z99	1

Confounding procedures in index spells (by OPCS-4 chapter)

(Potential rescue procedures included in this list)

OPCS chapter	Frequency
A	33
B	8
C	2
D	1
E	11
F	3
G	35
H	418
J	13
K	8
L	36
M	2705
O	8
P	28652
Q	11026
S	361
T	779
W	6
X	274

```
           Y      1159
           Z      1594


Summary of split cohorts
------------------------
34704 index spells with TOT introduction only
   25509 unconfounded index spells with TOT introduction only
   9195 confounded index spells with TOT introduction only
56648 index spells with TVT introduction only
   41880 unconfounded index spells with TVT introduction only
   14768 confounded index spells with TVT introduction only
834 index spells with minisling introduction only
   613 unconfounded index spells with minisling introduction only
   221 confounded index spells with minisling introduction only
60 index spells with combination (or none)
   0 unconfounded index spells with combination (or none)
   60 confounded index spells with combination (or none)

Unconfounded cohort characteristics
-----------------------------------
68002 all patients
   50 ( 44 to 61 ) median (IQR) age, years
   179 different providers
   67932 elective admissions
   1 ( 0 to 1 ) median (IQR) LoS, days
   9537 / 68002 ( 14.02 %) staying longer than 1 night
   LoS distribution:    LoS  freq
      0 31445
      1 26937
      2  6386
      3  1762
      4   701
      5   317
      6   131
      7    95
      8    54
      9    36
     10    14
     11     6
     12     8
     13     3
     14     2
     15    10
     16     3
     17     1
     19     2
     20     1
     21     1
     25     2
     31     1
     33     1
68002 patients with one of TVT, TOT or MS
   50 ( 44 to 61 ) median (IQR) age, years
   179 different providers
   67932 elective admissions
   1 ( 0 to 1 ) median (IQR) LoS, days
   9537 / 68002 ( 14.02 %) staying longer than 1 night
   LoS distribution:    LoS  freq
      0 31445
      1 26937
      2  6386
      3  1762
      4   701
      5   317
      6   131
      7    95
      8    54
      9    36
     10    14
     11     6
     12     8
     13     3
```

```
     14    2
     15   10
     16    3
     17    1
     19    2
     20    1
     21    1
     25    2
     31    1
     33    1
25509 patients with TOT only
   51 ( 44 to 61 ) median (IQR) age, years
   170 different providers
   25490 elective admissions
   1 ( 0 to 1 ) median (IQR) LoS, days
   0 in-hospital deaths
   5473 admissions with endoscopic examination of bladder (M45)
   14 admissions with endoscopic examination of urethra (M77)
   5477 admissions with endoscopic examination of urethra or bladder
   444 admissions with catheterization
41880 patients with TVT only
   50 ( 44 to 60 ) median (IQR) age, years
   175 different providers
   41831 elective admissions
   1 ( 0 to 1 ) median (IQR) LoS, days
   0 in-hospital deaths
   11460 admissions with endoscopic examination of bladder (M45)
   13 admissions with endoscopic examination of urethra (M77)
   11466 admissions with endoscopic examination of urethra or bladder
   928 admissions with catheterization
613 patients with minisling only
   52 ( 45 to 62 ) median (IQR) age, years
   67 different providers
   611 elective admissions
   2 ( 0 to 4 ) median (IQR) LoS, days
   0 in-hospital deaths
   125 admissions with endoscopic examination of bladder (M45)
   0 admissions with endoscopic examination of urethra (M77)
   125 admissions with endoscopic examination of urethra or bladder
   11 admissions with catheterization
0 patients with combination of TVT, TOT, MS
   NA ( NA to NA ) median (IQR) age, years
   0 different providers
   0 elective admissions
   NA ( NA to NA ) median (IQR) LoS, days
   0 in-hospital deaths
   0 admissions with endoscopic examination of bladder (M45)
   0 admissions with endoscopic examination of urethra (M77)
   0 admissions with endoscopic examination of urethra or bladder
   0 admissions with catheterization

Confounded cohort characteristics
-----------------------------------
24244 all patients
   51 ( 44 to 62 ) median (IQR) age, years
   181 different providers
   24182 elective admissions
   2 ( 1 to 3 ) median (IQR) LoS, days
   15853 / 24244 ( 65.39 %) staying longer than 1 night
   LoS distribution:    LoS freq
      0 2563
      1 5750
      2 6940
      3 4807
      4 2131
      5  922
      6  439
      7  217
      8  150
      9   79
     10   39
     11   37
     12   16
```

```
      13   10
      14   10
      15    9
      16    3
      17    9
      18    5
      19    3
      20    3
      21    4
      22    5
      23    1
      24    2
      25    1
      26    1
      28    1
      29    1
      30    2
      31    1
      32    1
      38    1
      73    1
      78    1
      90    1
24184 patients with one of TVT, TOT or MS
   51 ( 44 to 62 ) median (IQR) age, years
   181 different providers
   24122 elective admissions
   2 ( 1 to 3 ) median (IQR) LoS, days
   15829 / 24184 ( 65.45 %) staying longer than 1 night
   LoS distribution:    LoS freq
       0 2544
       1 5733
       2 6928
       3 4802
       4 2128
       5  921
       6  436
       7  217
       8  150
       9   79
      10   39
      11   37
      12   16
      13   10
      14   10
      15    9
      16    3
      17    9
      18    5
      19    3
      20    3
      21    4
      22    5
      23    1
      24    2
      25    1
      26    1
      28    1
      29    1
      30    2
      31    1
      32    1
      38    1
      73    1
      78    1
      90    1
9195 patients with TOT only
   51 ( 45 to 63 ) median (IQR) age, years
   158 different providers
   9180 elective admissions
   2 ( 1 to 3 ) median (IQR) LoS, days
   0 in-hospital deaths
   1825 admissions with endoscopic examination of bladder (M45)
```

```
   2 admissions with endoscopic examination of urethra (M77)
   1825 admissions with endoscopic examination of urethra or bladder
   324 admissions with catheterization
14768 patients with TVT only
   51 ( 44 to 62 ) median (IQR) age, years
   172 different providers
   14722 elective admissions
   2 ( 1 to 3 ) median (IQR) LoS, days
   0 in-hospital deaths
   3780 admissions with endoscopic examination of bladder (M45)
   2 admissions with endoscopic examination of urethra (M77)
   3781 admissions with endoscopic examination of urethra or bladder
   759 admissions with catheterization
221 patients with minisling only
   50 ( 41 to 63 ) median (IQR) age, years
   54 different providers
   220 elective admissions
   3 ( 2 to 7 ) median (IQR) LoS, days
   0 in-hospital deaths
   53 admissions with endoscopic examination of bladder (M45)
   0 admissions with endoscopic examination of urethra (M77)
   53 admissions with endoscopic examination of urethra or bladder
   27 admissions with catheterization
60 patients with combination of TVT, TOT, MS
   49 ( 42 to 63.25 ) median (IQR) age, years
   35 different providers
   60 elective admissions
   1 ( 0 to 2 ) median (IQR) LoS, days
   0 in-hospital deaths
   20 admissions with endoscopic examination of bladder (M45)
   0 admissions with endoscopic examination of urethra (M77)
   20 admissions with endoscopic examination of urethra or bladder
   1 admissions with catheterization

Summary of peri-procedural complications in both parts of cohort
----------------------------------------------------------------
2866 spells with complication
   1618 spells in unconfounded operations
     356 TOT
     1232 TVT
     30 minisling
     0 combination
   1248 spells in confounded operations
     342 TOT
     880 TVT
     23 minisling
     3 combination

Complications in unconfounded operations
----------------------------------------
All reported complications:
   1271 TVT
   365 TOT
   34 MS
   0 comb

Complication code (no supplementary code):
 CompCode TVT TOT MS comb
      G97   1   0  0    0
      I97   0   3  0    0
      J95   0   2  0    0
      N99  43  11  0    0
      T81 409  69 18    0
      T82   2   0  0    0
      T83  15   9  2    0
      T88   8   6  0    0

Complication code (with supplementary code):
 CompCode TVT TOT MS comb
      K91   1   0  0    0
      N99   3   0  0    0
      T80   1   0  0    0
      T81 234  22  3    0
```

T83	11	2	0	0
T85	1	0	0	0
T88	6	3	0	0

By main diagnosis code (with supplementary code):

MainCode	TVT	TOT	MS	comb
A09	1	0	0	0
B96	2	1	0	0
D64	3	1	0	0
E03	1	2	0	0
E27	2	0	0	0
E87	3	0	0	0
E89	0	2	0	0
G21	0	1	0	0
G40	2	0	0	0
G62	0	1	0	0
G81	0	1	0	0
H83	1	0	0	0
I10	4	1	0	0
I48	2	3	1	0
I64	0	1	0	0
I80	1	0	0	0
I95	6	4	0	0
J18	0	1	1	0
J22	2	1	0	0
J44	1	0	0	0
J45	1	0	0	0
J98	3	0	0	0
K21	1	0	0	0
K44	0	1	0	0
K52	1	0	0	0
K59	0	1	1	0
L02	0	1	0	0
L27	2	0	0	0
L29	2	1	0	0
M25	1	4	0	0
M54	0	3	0	0
M79	4	7	0	0
N19	2	0	0	0
N32	2	1	0	0
N39	13	5	2	0
N81	1	0	0	0
N84	0	1	0	0
N89	0	2	0	0
N93	0	3	0	0
N94	0	1	0	0
N99	1	0	0	0
R00	7	1	0	0
R03	6	1	0	0
R07	5	2	0	0
R10	7	3	0	0
R11	9	2	1	0
R14	1	0	0	0
R20	0	1	0	0
R21	1	0	0	0
R25	0	1	0	0
R29	0	1	0	0
R30	1	0	1	0
R31	12	1	0	0
R32	3	0	0	0
R33	350	152	3	0
R35	0	1	0	0
R39	12	10	0	0
R40	2	0	0	0
R42	5	3	0	0
R46	0	0	1	0
R47	1	0	0	0
R50	2	0	0	0
R51	5	0	0	0
R52	1	0	0	0
R55	14	4	0	0
S31	1	0	0	0
S37	23	2	0	0

T31	1	0	0	0
T45	0	1	0	0
T78	1	0	0	0
Z86	0	1	0	0
Z92	1	0	0	0

By supplementary code:

SuppCode	TVT	TOT	MS	comb
Y40	3	1	0	0
Y41	0	1	0	0
Y42	3	1	0	0
Y43	3	1	0	0
Y45	7	5	1	0
Y47	1	0	0	0
Y48	6	1	0	0
Y49	1	1	0	0
Y52	2	0	0	0
Y56	0	1	0	0
Y57	3	1	0	0
Y60	165	12	0	0
Y69	1	0	0	0
Y73	4	0	0	0
Y76	2	0	0	0
Y81	2	0	0	0
Y82	1	0	0	0
Y83	577	237	13	0
Y84	12	3	0	0

By type of complication:

765 spells with procedure complication (774 complications)
- 656 TVT
- 90 TOT
- 19 MS
- 0 combinations

115 spells with device complication (116 complications)
- 83 TVT
- 28 TOT
- 4 MS
- 0 combinations

592 spells with complication with urinary symptoms (594 complications)
- 416 TVT
- 172 TOT
- 4 MS
- 0 combinations

222 spells with other complication
- 140 TVT
- 76 TOT
- 6 MS
- 0 combinations

Most common complications:

Complication code (with or without supplementary code):

CompCode	TVT	TOT	MS	sum
T81	643	91	21	755
N99	46	11	0	57
T83	26	11	2	39
T88	14	9	0	23
I97	0	3	0	3
J95	0	2	0	2
T82	2	0	0	2
G97	1	0	0	1
K91	1	0	0	1
T80	1	0	0	1
T85	1	0	0	1

Main code (with supplementary code):

MainCode	TVT	TOT	MS	sum
R33	350	152	3	505
S37	23	2	0	25
R39	12	10	0	22
N39	13	5	2	20
R55	14	4	0	18
R31	12	1	0	13

```
     R11   9   2  1  12
     M79   4   7  0  11
     I95   6   4  0  10
     R10   7   3  0  10
     R00   7   1  0   8
     R42   5   3  0   8
     R03   6   1  0   7
     R07   5   2  0   7
     I48   2   3  1   6
     I10   4   1  0   5
     M25   1   4  0   5
     R51   5   0  0   5
     D64   3   1  0   4
     B96   2   1  0   3
     E03   1   2  0   3
     E87   3   0  0   3
     J22   2   1  0   3
     J98   3   0  0   3
     L29   2   1  0   3
     M54   0   3  0   3
     N32   2   1  0   3
     N93   0   3  0   3
     R32   3   0  0   3
     E27   2   0  0   2
     E89   0   2  0   2
     G40   2   0  0   2
     J18   0   1  1   2
     K59   0   1  1   2
     L27   2   0  0   2
     N19   2   0  0   2
     N89   0   2  0   2
     R30   1   0  1   2
     R40   2   0  0   2
     R50   2   0  0   2
     A09   1   0  0   1
     G21   0   1  0   1
     G62   0   1  0   1
     G81   0   1  0   1
     H83   1   0  0   1
     I64   0   1  0   1
     I80   1   0  0   1
     J44   1   0  0   1
     J45   1   0  0   1
     K21   1   0  0   1
     K44   0   1  0   1
     K52   1   0  0   1
     L02   0   1  0   1
     N81   1   0  0   1
     N84   0   1  0   1
     N94   0   1  0   1
     N99   1   0  0   1
     R14   1   0  0   1
     R20   0   1  0   1
     R21   1   0  0   1
     R25   0   1  0   1
     R29   0   1  0   1
     R35   0   1  0   1
     R46   0   0  1   1
     R47   1   0  0   1
     R52   1   0  0   1
     S31   1   0  0   1
     T31   1   0  0   1
     T45   0   1  0   1
     T78   1   0  0   1
     Z86   0   1  0   1
     Z92   1   0  0   1

 Complication code (with or without supplementary code) in 'other' group:
CompCode TVT TOT MS sum
     T88  14   9  0  23
     T83   4   5  1  10
     N99   7   2  0   9
     I97   0   3  0   3
```

J95	0	2	0	2
T82	2	0	0	2
G97	1	0	0	1
K91	1	0	0	1
T80	1	0	0	1

Main code (with supplementary code) in 'other' group:

MainCode	TVT	TOT	MS	sum
N39	12	3	2	17
R55	11	3	0	14
M79	4	6	0	10
R11	6	2	0	8
I95	5	2	0	7
R00	6	1	0	7
R07	5	2	0	7
R10	5	1	0	6
R42	3	3	0	6
I48	2	2	1	5
R51	5	0	0	5
S37	4	1	0	5
I10	3	1	0	4
R03	4	0	0	4
D64	2	1	0	3
E03	1	2	0	3
J22	2	1	0	3
L29	2	1	0	3
M25	1	2	0	3
M54	0	3	0	3
N32	2	1	0	3
B96	1	1	0	2
E27	2	0	0	2
E87	2	0	0	2
E89	0	2	0	2
G40	2	0	0	2
J18	0	1	1	2
J98	2	0	0	2
L27	2	0	0	2
N19	2	0	0	2
N93	0	2	0	2
R40	2	0	0	2
A09	1	0	0	1
G21	0	1	0	1
G81	0	1	0	1
H83	1	0	0	1
J45	1	0	0	1
K21	1	0	0	1
K44	0	1	0	1
K52	1	0	0	1
K59	0	1	0	1
L02	0	1	0	1
N81	1	0	0	1
N84	0	1	0	1
N89	0	1	0	1
N94	0	1	0	1
N99	1	0	0	1
R14	1	0	0	1
R20	0	1	0	1
R21	1	0	0	1
R25	0	1	0	1
R29	0	1	0	1
R46	0	0	1	1
R47	1	0	0	1
R50	1	0	0	1
S31	1	0	0	1
T31	1	0	0	1
T45	0	1	0	1
T78	1	0	0	1
Z86	0	1	0	1
Z92	1	0	0	1

Complications in confounded operations

All reported complications:

979 TVT
363 TOT
32 MS
3 comb

Complication code (no supplementary code):

CompCode	TVT	TOT	MS	comb
G97	1	0	0	0
I97	2	0	0	0
J95	0	2	0	0
K91	1	0	0	0
N99	40	16	0	0
T80	1	0	0	0
T81	344	125	16	1
T82	1	1	0	0
T83	31	7	2	0
T85	5	0	0	0
T88	10	3	0	0

Complication code (with supplementary code):

CompCode	TVT	TOT	MS	comb
G97	1	0	0	0
K91	1	0	0	0
T81	140	41	4	1
T83	10	9	1	1
T84	1	0	0	0
T85	0	3	0	0
T88	2	2	0	0

By main diagnosis code (with supplementary code):

MainCode	TVT	TOT	MS	comb
A09	1	0	0	0
B37	0	0	3	0
B96	5	1	0	0
D50	1	0	0	0
D62	1	0	0	0
D64	8	0	0	0
E03	2	0	0	0
E11	1	0	0	0
E86	1	0	0	0
E87	1	0	0	0
E89	1	0	0	0
G40	1	0	0	0
G80	0	1	0	0
H00	1	0	0	0
I10	1	0	0	0
I21	1	0	0	0
I26	3	0	0	0
I44	1	0	0	0
I46	1	1	0	0
I47	1	1	0	0
I48	4	0	0	0
I95	7	3	0	0
J06	1	0	0	0
J18	8	1	0	0
J22	9	1	1	0
J44	0	0	1	0
J69	1	0	0	0
J90	1	0	0	0
J96	2	1	0	0
J98	1	1	1	0
K21	1	0	0	0
K29	1	0	0	0
K52	1	2	0	0
K56	3	0	0	0
K59	5	2	0	0
K62	1	0	0	0
K82	0	1	0	0
L03	1	0	0	0
L27	2	0	0	0
L50	1	0	0	0
L90	2	0	0	0
M54	1	0	0	0

M79	2	1	0	0
N17	1	1	0	0
N28	1	0	0	0
N32	1	0	0	0
N39	29	10	1	0
N81	1	1	0	0
N82	1	0	0	0
N85	0	1	0	0
N86	1	0	0	0
N89	1	1	0	0
N90	4	2	0	0
N93	2	1	0	0
R00	8	3	0	0
R03	3	1	0	0
R06	3	3	0	0
R07	2	1	0	0
R10	6	3	0	0
R11	6	4	0	0
R14	0	1	0	0
R19	1	0	0	0
R20	1	1	0	0
R21	1	1	0	0
R25	0	1	0	0
R30	0	1	0	0
R31	3	0	0	0
R32	1	0	0	0
R33	185	86	2	0
R35	1	0	0	0
R39	3	1	0	0
R42	0	1	0	0
R50	6	2	0	0
R51	1	0	0	0
R55	2	3	0	0
R73	1	0	0	0
R94	1	0	0	0
S00	2	0	0	0
S05	0	3	0	0
S30	1	0	0	0
S31	2	1	0	0
S37	13	2	0	0
S39	2	0	0	0
T17	1	0	0	0
T19	1	0	0	0
T24	1	0	0	0
T45	0	1	0	0
T91	1	0	0	0

By supplementary code:

SuppCode	TVT	TOT	MS	comb
Y40	3	1	0	0
Y41	1	0	0	0
Y43	0	1	0	0
Y44	0	1	0	0
Y45	2	6	0	0
Y48	6	1	0	0
Y51	1	2	0	0
Y52	2	0	0	0
Y58	0	1	0	0
Y60	74	17	2	0
Y61	0	1	0	0
Y65	1	1	0	0
Y69	1	0	0	0
Y70	2	0	0	0
Y73	0	1	0	0
Y76	1	2	0	0
Y81	1	0	0	0
Y82	1	0	0	0
Y83	439	170	12	2
Y84	8	4	0	0

By type of complication:

633 spells with procedure complication (682 complications)

453 TVT

161 TOT
17 MS
2 combinations
84 spells with device complication (86 complications)
62 TVT
21 TOT
0 MS
1 combinations
311 spells with complication with urinary symptoms (312 complications)
210 TVT
99 TOT
2 MS
0 combinations
286 spells with other complication
204 TVT
76 TOT
6 MS
0 combinations

Most common complications:
Complication code (with or without supplementary code):

CompCode	TVT	TOT	MS	sum
T81	484	166	20	670
T83	41	16	3	60
N99	40	16	0	56
T88	12	5	0	17
T85	5	3	0	8
G97	2	0	0	2
I97	2	0	0	2
J95	0	2	0	2
K91	2	0	0	2
T82	1	1	0	2
T80	1	0	0	1
T84	1	0	0	1

Main code (with supplementary code):

MainCode	TVT	TOT	MS	sum
R33	185	86	2	273
N39	29	10	1	40
S37	13	2	0	15
J22	9	1	1	11
R00	8	3	0	11
I95	7	3	0	10
R11	6	4	0	10
J18	8	1	0	9
R10	6	3	0	9
D64	8	0	0	8
R50	6	2	0	8
K59	5	2	0	7
B96	5	1	0	6
N90	4	2	0	6
R06	3	3	0	6
R55	2	3	0	5
I48	4	0	0	4
R03	3	1	0	4
R39	3	1	0	4
B37	0	0	3	3
I26	3	0	0	3
J96	2	1	0	3
J98	1	1	1	3
K52	1	2	0	3
K56	3	0	0	3
M79	2	1	0	3
N93	2	1	0	3
R07	2	1	0	3
R31	3	0	0	3
S05	0	3	0	3
S31	2	1	0	3
E03	2	0	0	2
I46	1	1	0	2
I47	1	1	0	2
L27	2	0	0	2
L90	2	0	0	2

```
      N17   1   1  0   2
      N81   1   1  0   2
      N89   1   1  0   2
      R20   1   1  0   2
      R21   1   1  0   2
      S00   2   0  0   2
      S39   2   0  0   2
      A09   1   0  0   1
      D50   1   0  0   1
      D62   1   0  0   1
      E11   1   0  0   1
      E86   1   0  0   1
      E87   1   0  0   1
      E89   1   0  0   1
      G40   1   0  0   1
      G80   0   1  0   1
      H00   1   0  0   1
      I10   1   0  0   1
      I21   1   0  0   1
      I44   1   0  0   1
      J06   1   0  0   1
      J44   0   0  1   1
      J69   1   0  0   1
      J90   1   0  0   1
      K21   1   0  0   1
      K29   1   0  0   1
      K62   1   0  0   1
      K82   0   1  0   1
      L03   1   0  0   1
      L50   1   0  0   1
      M54   1   0  0   1
      N28   1   0  0   1
      N32   1   0  0   1
      N82   1   0  0   1
      N85   0   1  0   1
      N86   1   0  0   1
      R14   0   1  0   1
      R19   1   0  0   1
      R25   0   1  0   1
      R30   0   1  0   1
      R32   1   0  0   1
      R35   1   0  0   1
      R42   0   1  0   1
      R51   1   0  0   1
      R73   1   0  0   1
      R94   1   0  0   1
      S30   1   0  0   1
      T17   1   0  0   1
      T19   1   0  0   1
      T24   1   0  0   1
      T45   0   1  0   1
      T91   1   0  0   1

additional complication analysis - see 'table' folder



----------------------------
30 day complication analysis
----------------------------

92246 eligible index spells with admission date
505428 total episodes for patients with an eligible index spell
101979 episodes on day of index spell or within 30 days
   92246 patients
9255 episodes on day of index spell or within 30 days, not including index episodes
   7195 patients
     4850 unconfounded patients
       1485 TOT
       3255 TVT
       110 MS
       0 comb
     2345 confounded patients
```

```
       785 TOT
       1502 TVT
       54 MS
       4 comb
   109 episodes on day zero (day of index admission)
   8807 spells of admission within 30 days
     5904 spells for unconfounded patients
       1749 TOT
       4025 TVT
       130 MS
       0 comb
     2903 spells for confounded patients
       930 TOT
       1898 TVT
       70 MS
       5 comb
2180 thirty day episodes admitted for a complication
   2082 spells
   1876 patients
   21 episodes on day zero (day of index admission)
1330 thirty day episodes admitted for complication following unconfounded operation:
   326 episodes, 309 spells, 275 patients (TOT)
     31 spells, 30 patients have re-intervention (TOT)
   965 episodes, 931 spells, 833 patients (TVT)
     144 spells, 141 patients have re-intervention (TVT)
   39 episodes, 33 spells, 29 patients (MS)
     0 spells, 0 patients have re-intervention (MS)
   0 episodes, 0 spells, 0 patients (comb)
     0 spells, 0 patients have re-intervention (comb)
850 thirty day episodes admitted for complication following confounded operation:
   281 episodes, 268 spells, 250 patients (TOT)
     22 spells, 22 patients have re-intervention (TOT)
   557 episodes, 529 spells, 479 patients (TVT)
     61 spells, 61 patients have re-intervention (TVT)
   10 episodes, 10 spells,  9 patients (MS)
     0 spells, 0 patients have re-intervention (MS)
   2 episodes, 2 spells, 1 patients (comb)
     1 spells, 1 patients have re-intervention (comb)

Complications in unconfounded operations
----------------------------------------
All reported complications:
   965 TVT
   326 TOT
   39 MS
   0 comb

Complication code (no supplementary code):
 CompCode TVT TOT MS comb
      G97   3   4  0    0
      J95   0   2  0    0
      N99  31  11  0    0
      T81  78  26  6    0
      T82   1   0  0    0
      T83  69  17  4    0
      T84   0   1  1    0
      T85   3   5  0    0

Complication code (with supplementary code):
 CompCode TVT TOT MS comb
      K91   0   1  0    0
      N99   3   0  0    0
      T80   1   0  0    0
      T81 190  64 16    0
      T83 106  40  3    0
      T84   2   0  0    0
      T85   9   1  0    0
      T88   7   2  0    0

By main diagnosis code (with supplementary code):
 MainCode TVT TOT MS comb
      A40   1   0  0    0
      A41   1   0  0    0
```

E10	2	0	0	0
E86	1	0	0	0
E87	1	0	0	0
I26	1	0	0	0
I42	1	0	0	0
I74	1	0	0	0
I80	0	1	0	0
K22	1	0	0	0
K52	1	0	0	0
K59	7	2	0	0
K65	0	0	1	0
L03	3	0	0	0
L27	1	0	0	0
L53	1	0	0	0
M25	0	1	0	0
M54	0	1	0	0
M62	0	1	0	0
M72	0	2	0	0
M79	1	8	0	0
N12	3	0	0	0
N30	1	0	0	0
N35	1	0	0	0
N39	32	9	2	0
N76	2	0	0	0
N81	1	0	0	0
N89	4	5	0	0
N90	2	0	0	0
N93	9	6	0	0
N94	1	2	0	0
R03	1	0	0	0
R07	1	1	0	0
R10	46	14	0	0
R11	8	0	0	0
R14	1	0	0	0
R18	0	1	0	0
R29	1	0	0	0
R30	2	4	0	0
R31	9	2	1	0
R32	4	4	0	0
R33	255	77	5	0
R35	5	0	0	0
R39	37	6	0	0
R50	1	0	0	0
R52	1	0	0	0
R55	1	0	0	0
R69	0	1	0	0
S30	1	0	0	0
S31	1	0	0	0
S37	1	0	0	0
S70	0	1	0	0
T37	0	1	0	0
T39	1	0	0	0
T78	1	0	0	0
Z03	0	1	0	0
Z45	1	0	0	0
Z46	3	1	0	0

By supplementary code:

SuppCode	TVT	TOT	MS	comb
Y40	2	0	0	0
Y41	3	1	0	0
Y44	1	0	0	0
Y45	2	1	0	0
Y48	1	0	0	0
Y49	2	0	0	0
Y51	1	0	0	0
Y53	0	1	0	0
Y57	1	0	0	0
Y60	3	0	0	0
Y73	2	0	0	0
Y76	5	3	0	0
Y81	2	0	0	0
Y83	728	236	26	0

```
      Y84  27  18  2    0

By type of complication:
   357 spells with procedure complication ( 381 complications)
      253 TVT
      86 TOT
      18 MS
      0 combinations
   319 spells with device complication ( 332 complications)
      233 TVT
      81 TOT
      5 MS
      0 combinations
   445 spells with complication with urinary symptoms ( 454 complications)
      340 TVT
      99 TOT
      6 MS
      0 combinations
   217 spells with other complication
      151 TVT
      60 TOT
      6 MS
      0 combinations

Most common complications:
  Complication code (with or without supplementary code):
 CompCode TVT TOT MS sum
      T81 268  90 22 380
      T83 175  57  7 239
      N99  34  11  0  45
      T85  12   6  0  18
      T88   7   2  0   9
      G97   3   4  0   7
      T84   2   1  1   4
      J95   0   2  0   2
      K91   0   1  0   1
      T80   1   0  0   1
      T82   1   0  0   1

  Main code (with supplementary code):
 MainCode TVT TOT MS sum
      R33 255  77  5 337
      R10  46  14  0  60
      N39  32   9  2  43
      R39  37   6  0  43
      N93   9   6  0  15
      R31   9   2  1  12
      K59   7   2  0   9
      M79   1   8  0   9
      N89   4   5  0   9
      R11   8   0  0   8
      R32   4   4  0   8
      R30   2   4  0   6
      R35   5   0  0   5
      Z46   3   1  0   4
      L03   3   0  0   3
      N12   3   0  0   3
      N94   1   2  0   3
      E10   2   0  0   2
      M72   0   2  0   2
      N76   2   0  0   2
      N90   2   0  0   2
      R07   1   1  0   2
      A40   1   0  0   1
      A41   1   0  0   1
      E86   1   0  0   1
      E87   1   0  0   1
      I26   1   0  0   1
      I42   1   0  0   1
      I74   1   0  0   1
      I80   0   1  0   1
      K22   1   0  0   1
      K52   1   0  0   1
```

K65	0	0	1	1
L27	1	0	0	1
L53	1	0	0	1
M25	0	1	0	1
M54	0	1	0	1
M62	0	1	0	1
N30	1	0	0	1
N35	1	0	0	1
N81	1	0	0	1
R03	1	0	0	1
R14	1	0	0	1
R18	0	1	0	1
R29	1	0	0	1
R50	1	0	0	1
R52	1	0	0	1
R55	1	0	0	1
R69	0	1	0	1
S30	1	0	0	1
S31	1	0	0	1
S37	1	0	0	1
S70	0	1	0	1
T37	0	1	0	1
T39	1	0	0	1
T78	1	0	0	1
Z03	0	1	0	1
Z45	1	0	0	1

Complication code (with or without supplementary code) in 'other' group:

CompCode	TVT	TOT	MS	sum
T83	31	10	3	44
G97	3	4	0	7
T88	6	1	0	7
J95	0	2	0	2
N99	1	1	0	2
T84	0	1	1	2
K91	0	1	0	1
T80	1	0	0	1
T82	1	0	0	1

Main code (with supplementary code) in 'other' group:

MainCode	TVT	TOT	MS	sum
R10	33	10	0	43
N39	27	8	2	37
N89	4	4	0	8
N93	6	2	0	8
M79	0	7	0	7
R11	7	0	0	7
K59	4	2	0	6
Z46	3	1	0	4
N12	3	0	0	3
E10	2	0	0	2
L03	2	0	0	2
M72	0	2	0	2
N76	2	0	0	2
R07	1	1	0	2
A41	1	0	0	1
E86	1	0	0	1
E87	1	0	0	1
I26	1	0	0	1
I42	1	0	0	1
I74	1	0	0	1
K22	1	0	0	1
K52	1	0	0	1
L27	1	0	0	1
L53	1	0	0	1
M25	0	1	0	1
M54	0	1	0	1
M62	0	1	0	1
N81	1	0	0	1
N90	1	0	0	1
N94	1	0	0	1
R14	1	0	0	1
R18	0	1	0	1

R29	1	0	0	1
R50	1	0	0	1
R52	1	0	0	1
R55	1	0	0	1
R69	0	1	0	1
S30	1	0	0	1
S70	0	1	0	1
T37	0	1	0	1
T39	1	0	0	1
T78	1	0	0	1
Z03	0	1	0	1

Complications in confounded operations

All reported complications:
- 557 TVT
- 281 TOT
- 10 MS
- 2 comb

Complication code (no supplementary code):

CompCode	TVT	TOT	MS	comb
G97	4	2	0	0
N99	17	4	0	0
T81	61	34	3	0
T83	31	16	0	0
T84	0	2	0	0
T85	0	1	0	0
T88	1	0	0	0

Complication code (with supplementary code):

CompCode	TVT	TOT	MS	comb
G97	0	1	0	0
N99	1	0	0	0
T81	210	117	3	0
T82	1	0	0	0
T83	41	19	2	0
T84	0	1	0	0
T85	4	1	0	0
T88	3	2	0	0

By main diagnosis code (with supplementary code):

MainCode	TVT	TOT	MS	comb
D64	1	0	0	0
G58	1	1	0	0
H91	1	0	0	0
I21	1	0	0	0
I80	0	1	0	0
J18	1	0	0	0
J98	1	0	0	0
K43	0	1	0	0
K52	0	1	0	0
K56	2	0	0	0
K59	6	2	0	0
K62	3	0	0	0
L03	2	0	0	0
M25	0	1	0	0
M54	0	1	0	0
M79	1	4	0	0
N12	1	0	0	0
N31	2	0	0	0
N32	1	1	0	0
N35	1	0	0	0
N39	19	5	0	0
N73	4	0	0	0
N76	1	0	0	0
N82	0	1	0	0
N89	4	2	0	0
N93	13	4	0	0
N94	0	2	0	0
R10	9	8	0	0
R18	1	0	0	0
R21	1	0	0	0

R30	3	0	0	0
R31	0	0	1	0
R32	2	0	0	0
R33	83	42	1	2
R39	13	2	0	0
R51	0	1	0	0
R53	1	0	0	0
R94	1	0	0	0
S34	1	0	0	0
Z46	2	1	0	0

By supplementary code:

SuppCode	TVT	TOT	MS	comb
Y40	0	1	0	0
Y41	1	0	0	0
Y44	2	0	0	0
Y48	0	1	0	0
Y51	1	0	0	0
Y82	1	0	0	0
Y83	423	215	7	2
Y84	15	5	0	0

By type of complication:

- 407 spells with procedure complication (428 complications)
  - 256 TVT
  - 145 TOT
  - 6 MS
  - 0 combinations
- 132 spells with device complication (138 complications)
  - 89 TVT
  - 42 TOT
  - 1 MS
  - 0 combinations
- 163 spells with complication with urinary symptoms (169 complications)
  - 112 TVT
  - 47 TOT
  - 2 MS
  - 2 combinations
- 139 spells with other complication
  - 93 TVT
  - 44 TOT
  - 2 MS
  - 0 combinations

Most common complications:

Complication code (with or without supplementary code):

CompCode	TVT	TOT	MS	sum
T81	271	151	6	428
T83	72	35	2	109
N99	18	4	0	22
G97	4	3	0	7
T85	4	2	0	6
T88	4	2	0	6
T84	0	3	0	3
T82	1	0	0	1

Main code (with supplementary code):

MainCode	TVT	TOT	MS	sum
R33	83	42	1	126
N39	19	5	0	24
N93	13	4	0	17
R10	9	8	0	17
R39	13	2	0	15
K59	6	2	0	8
N89	4	2	0	6
M79	1	4	0	5
N73	4	0	0	4
K62	3	0	0	3
R30	3	0	0	3
Z46	2	1	0	3
G58	1	1	0	2
K56	2	0	0	2
L03	2	0	0	2

N31	2	0	0	2
N32	1	1	0	2
N94	0	2	0	2
R32	2	0	0	2
D64	1	0	0	1
H91	1	0	0	1
I21	1	0	0	1
I80	0	1	0	1
J18	1	0	0	1
J98	1	0	0	1
K43	0	1	0	1
K52	0	1	0	1
M25	0	1	0	1
M54	0	1	0	1
N12	1	0	0	1
N35	1	0	0	1
N76	1	0	0	1
N82	0	1	0	1
R18	1	0	0	1
R21	1	0	0	1
R31	0	0	1	1
R51	0	1	0	1
R53	1	0	0	1
R94	1	0	0	1
S34	1	0	0	1

```
30 day admissions without complication
--------------------------------------
6725 thirty day spells without admission for complication
   5651 patients
   7037 episodes

30 day readmissions with no complication - whole cohort:
--------------------------------------------------------
7037 thirty day episodes
   6725 spells
   5651 patients
2911 thirty day episodes with catheter-related procedures
   2910 spells involving (but not limited to) catheter procedures
285 thirty day episodes with mesh-related re-interventions
   285 spells involving (but not limited to) mesh re-interventions
2436 thirty day episodes with no procedure
   2157 spells with no procedure
2963 episodes with no confounding procedure
   2658 spells no confounding procedure
   501 spells with procedures but no confounding procedures

30 day readmissions with no complication - unconfounded cohort:
---------------------------------------------------------------
4822 thirty day episodes
   4631 spells
   3922 patients
1966 thirty day episodes with catheter-related procedures
   1965 spells involving (but not limited to) catheter procedures
203 thirty day episodes with mesh-related re-interventions
   203 spells involving (but not limited to) mesh re-interventions
1587 thirty day episodes with no procedure
   1415 spells with no procedure
1971 episodes with no confounding procedure
   1783 spells no confounding procedure
   368 spells with procedures but no confounding procedures

30 day readmissions with no complication - unconfounded TVT:
------------------------------------------------------------
3208 thirty day episodes
   3094 spells
   2580 patients
1369 thirty day episodes with catheter-related procedures
   1369 spells involving (but not limited to) catheter procedures
138 thirty day episodes with mesh-related re-interventions
   138 spells involving (but not limited to) mesh re-interventions
1072 thirty day episodes with no procedure
```

```
   970 spells with no procedure
1295 episodes with no confounding procedure
   1182 spells no confounding procedure
   212 spells with procedures but no confounding procedures

30 day readmissions with no complication - unconfounded TOT:
------------------------------------------------------------
1514 thirty day episodes
   1440 spells
   1257 patients
547 thirty day episodes with catheter-related procedures
   546 spells involving (but not limited to) catheter procedures
62 thirty day episodes with mesh-related re-interventions
   62 spells involving (but not limited to) mesh re-interventions
485 thirty day episodes with no procedure
   417 spells with no procedure
638 episodes with no confounding procedure
   566 spells no confounding procedure
   149 spells with procedures but no confounding procedures

30 day readmissions with no complication - unconfounded MS:
------------------------------------------------------------
100 thirty day episodes
   97 spells
   85 patients
50 thirty day episodes with catheter-related procedures
   50 spells involving (but not limited to) catheter procedures
3 thirty day episodes with mesh-related re-interventions
   3 spells involving (but not limited to) mesh re-interventions
30 thirty day episodes with no procedure
   28 spells with no procedure
38 episodes with no confounding procedure
   35 spells no confounding procedure
   7 spells with procedures but no confounding procedures

30 day readmissions with no complication - confounded cohort:
-------------------------------------------------------------
2215 thirty day episodes
   2094 spells
   1729 patients
945 thirty day episodes with catheter-related procedures
   945 spells involving (but not limited to) catheter procedures
82 thirty day episodes with mesh-related re-interventions
   82 spells involving (but not limited to) mesh re-interventions
849 thirty day episodes with no procedure
   742 spells with no procedure
992 episodes with no confounding procedure
   875 spells no confounding procedure
   133 spells with procedures but no confounding procedures

30 day readmissions with no complication - confounded TVT:
----------------------------------------------------------
1454 thirty day episodes
   1369 spells
   1102 patients
633 thirty day episodes with catheter-related procedures
   633 spells involving (but not limited to) catheter procedures
58 thirty day episodes with mesh-related re-interventions
   58 spells involving (but not limited to) mesh re-interventions
550 thirty day episodes with no procedure
   476 spells with no procedure
639 episodes with no confounding procedure
   557 spells no confounding procedure
   81 spells with procedures but no confounding procedures

30 day readmissions with no complication - confounded TOT:
----------------------------------------------------------
695 thirty day episodes
   662 spells
   578 patients
276 thirty day episodes with catheter-related procedures
   276 spells involving (but not limited to) catheter procedures
23 thirty day episodes with mesh-related re-interventions
```

```
   23 spells involving (but not limited to) mesh re-interventions
278 thirty day episodes with no procedure
   247 spells with no procedure
328 episodes with no confounding procedure
   296 spells no confounding procedure
   49 spells with procedures but no confounding procedures

30 day readmissions with no complication - confounded MS:
-------------------------------------------------------
63 thirty day episodes
   60 spells
   46 patients
33 thirty day episodes with catheter-related procedures
   33 spells involving (but not limited to) catheter procedures
1 thirty day episodes with mesh-related re-interventions
   1 spells involving (but not limited to) mesh re-interventions
21 thirty day episodes with no procedure
   19 spells with no procedure
25 episodes with no confounding procedure
   22 spells no confounding procedure
   3 spells with procedures but no confounding procedures


------------------------
Post-procedural analysis
------------------------

Counting episodes pre- and post- index
--------------------------------------
561631 total episodes
101364 episodes in index spells
193330 episodes before index spells
266937 episodes after index spells

In-hospital deaths
------------------
918 in-hospital death episodes
860 in-hospital death spells
767 in-hospital death spells for SUI cohort
0 patients died before index procedure
1 duplicated PSEUDO_HESID in death events
766 in-hospital death spells
   766 different patients
3 in-hospital death spells within 30 days
    3 different patients
2 in-hospital death spells within 30 days (unconfounded cohort)
    2 different patients (unconfounded cohort)

Patients who survived to the end of study
-----------------------------------------
91480 patients alive at end of study period
0 patients who were in both dead and alive cohorts
92246 patients in SUI cohort with index procedure
142234241 total follow-up (days)
1541.9 days mean follow-up

Admissions due to relevant symptoms
-----------------------------------
10386 episodes admitted for pain
1324 episodes admitted for abnormal bleeding
4025 episodes admitted for previous procedural complication
3822 episodes admitted for previous complication of devices

3775 spells with admission for relevant symptoms (device complications only)
3194 spells admitted for relevant symptoms in SUI cohort
92 spells admitted for relevant symptoms before index procedure
3102 spells admitted for relevant symptoms after index procedure
13 spells admitted for relevant symptoms which are also index episodes - removed
264 spells admitted for relevant symptoms which are 30 day complication episodes - removed
2825 qualifying spells admitted for relevant symptoms for analysis

1 spells for pain; 1 patients
0 spells for bleeding; 0 patients
```

```
0 spells for previous complication; 0 patients
2825 spells for previous device complication; 2181 patients

Admissions with pain (in any field)
-----------------------------------
21924 episodes admitted with pain ( R100 R101 R102 R103 R104 R10X N930 N938 N939 N93X N941 M726 K613 )
20169 spells with admission with pain
18273 spells admitted with pain in SUI cohort
8423 spells admitted with pain before index procedure
9850 spells admitted with pain after index procedure
593 spells admitted with pain which are also index episodes - removed
147 spells admitted with pain which are 30 day complication episodes - removed
9110 qualifying spells admitted with pain for analysis
7846 spells with abdo+pelvic pain (R10); 5719 patients
793 spells with vaginal bleeding (N93); 742 patients
455 spells with dyspareunia (N94); 421 patients
6 spells with necrotising fasciitis (M72.6); 5 patients
10 spells with ischiorectal abscess (K61.3); 8 patients

Admissions including re-intervention
------------------------------------
107357 episodes including SUI mesh intervention
107341 spells with SUI mesh intervention
97898 spells with SUI mesh intervention in SUI cohort
0 spells with intervention for SUI mesh before index procedure
97898 spells with SUI intervention remaining
92246 spells with SUI intervention which are also index episodes - removed
260 spells admitted for intervention which are 30 day complication spells - removed
5392 qualifying spells admitted with SUI intervention for analysis
   1980 introductions
   968 repairs
   2607 removals
   8 renewals


draw example history figure
---------------------------
see figures/history.pdf

Unconfounded cohort
-------------------
68002 patients in cohort
552 died in hospital
67450 alive at end of study
2008 spells due to relevant symptoms
3886 spells with SUI mesh intervention
68002 patients in SUI cohort with index procedure
104561376 total follow-up (days), 286273.4 years
1537.6 days mean follow-up, 4.2 years
1574 days median follow-up, 4.3 years
   90.17 % followed up for > 1 year(s)
   78.39 % followed up for > 2 year(s)
   66.48 % followed up for > 3 year(s)
   53.77 % followed up for > 4 year(s)
   40.89 % followed up for > 5 year(s)
   27.53 % followed up for > 6 year(s)
   13.59 % followed up for > 7 year(s)
   0.01 % followed up for > 8 year(s)

Relevant symptom events:
   70010 total events
   68002 unique patients
   2008 admissions for symptom events
   7.01 per 1000 person years crude IR
   1541 unique patients
  Distribution of admissions for relevant symptoms:
    n  freq
    0 66461
    1  1178
    2   283
    3    60
    4    17
    5     2
```

```
    6     1
  Breakdown of admissions for symptoms:
     0 pain ( 0 patients)
     0 bleeding ( 0 patients)
     0 complication ( 0 patients)
     2008 device ( 1541 patients)

Further SUI interventions:
   71888 total events
   68002 unique patients
   3886 admissions for reintervention events
   13.57 per 1000 person years crude IR
   3213 unique patients
   Distribution of admissions for further mesh interventions:
    n  freq
    0 64789
    1  2674
    2   430
    3    89
    4    16
    5     3
    6     1
  Breakdown of further mesh interventions:
     1486 introductions ( 1451 patients)
     705 repairs ( 657 patients)
     1829 removals ( 1608 patients)
     6 renewals ( 6 patients)

Cross-tabulation of symptoms and re-intervention:
        Reop
Symptoms     0     1
       0 64461  2000
       1   329  1212

Re-intervention or symptoms event analysis:
   4575 admissions for relevant events (in unique spells)
   3542 patients
   15.98 per 1000 person years crude IR
   Distribution of admissions for relevant events:
    n  freq
    0 64460
    1  2788
    2   553
    3   146
    4    37
    5    13
    6     5

First relevant symptoms event analysis:
   2008 events after first event  (e.g. censor after first admission for relevant symptoms)
   68002 first events
   68002 unique patients
   1541 rows with symptom event
   14 rows with time=0 -> 0.5
   0 events with time=0 -> 0.5
   NA ( NA to NA ) years, 95% survival
   0.974 ( 0.973 to 0.976 ) 5 year survival probability

First re-intervention event analysis:
   3886 events after first event (e.g. censor after re-intervention)
   68002 first events
   68002 unique patients
   3212 rows with re-intervention event
   31 rows with time=0 -> 0.5
   17 events with time=0 -> 0.5
   4.2 ( 3.91 to 4.64 ) years, 95% survival
   0.946 ( 0.944 to 0.948 ) 5 year survival probability

First relevant event survival analysis:
   4575 events after first event  (e.g. censor after first symptoms or re-intervention)
   68002 first events
   68002 unique patients
   3541 rows with symptoms or re-intervention event
```

```
   31 rows with time=0 -> 0.5
   17 events with time=0 -> 0.5
   3.28 ( 2.9 to 3.7 ) years, 95% survival
   0.941 ( 0.939 to 0.943 ) 5 year survival probability

Unconfounded TVT, TOT or MS
---------------------------
68002 patients in cohort
552 died in hospital
67450 alive at end of study
2008 spells due to relevant symptoms
3886 spells with SUI mesh intervention
68002 patients in SUI cohort with index procedure
104561376 total follow-up (days), 286273.4 years
1537.6 days mean follow-up, 4.2 years
1574 days median follow-up, 4.3 years
   90.17 % followed up for > 1 year(s)
   78.39 % followed up for > 2 year(s)
   66.48 % followed up for > 3 year(s)
   53.77 % followed up for > 4 year(s)
   40.89 % followed up for > 5 year(s)
   27.53 % followed up for > 6 year(s)
   13.59 % followed up for > 7 year(s)
   0.01 % followed up for > 8 year(s)

Relevant symptom events:
   70010 total events
   68002 unique patients
   2008 admissions for symptom events
   7.01 per 1000 person years crude IR
   1541 unique patients
  Distribution of admissions for relevant symptoms:
    n  freq
    0 66461
    1  1178
    2   283
    3    60
    4    17
    5     2
    6     1
  Breakdown of admissions for symptoms:
     0 pain ( 0 patients)
     0 bleeding ( 0 patients)
     0 complication ( 0 patients)
     2008 device ( 1541 patients)

Further SUI interventions:
   71888 total events
   68002 unique patients
   3886 admissions for reintervention events
   13.57 per 1000 person years crude IR
   3213 unique patients
   Distribution of admissions for further mesh interventions:
    n  freq
    0 64789
    1  2674
    2   430
    3    89
    4    16
    5     3
    6     1
  Breakdown of further mesh interventions:
     1486 introductions ( 1451 patients)
     705 repairs ( 657 patients)
     1829 removals ( 1608 patients)
     6 renewals ( 6 patients)

Cross-tabulation of symptoms and re-intervention:
        Reop
Symptoms     0     1
       0 64461  2000
       1   329  1212
```

```
Re-intervention or symptoms event analysis:
   4575 admissions for relevant events (in unique spells)
   3542 patients
   15.98 per 1000 person years crude IR
   Distribution of admissions for relevant events:
    n  freq
    0 64460
    1  2788
    2   553
    3   146
    4    37
    5    13
    6     5

First relevant symptoms event analysis:
   2008 events after first event  (e.g. censor after first admission for relevant symptoms)
   68002 first events
   68002 unique patients
   1541 rows with symptom event
   14 rows with time=0 -> 0.5
   0 events with time=0 -> 0.5
   NA ( NA to NA ) years, 95% survival
   0.974 ( 0.973 to 0.976 ) 5 year survival probability

First re-intervention event analysis:
   3886 events after first event (e.g. censor after re-intervention)
   68002 first events
   68002 unique patients
   3212 rows with re-intervention event
   31 rows with time=0 -> 0.5
   17 events with time=0 -> 0.5
   4.2 ( 3.91 to 4.64 ) years, 95% survival
   0.946 ( 0.944 to 0.948 ) 5 year survival probability

First relevant event survival analysis:
   4575 events after first event  (e.g. censor after first symptoms or re-intervention)
   68002 first events
   68002 unique patients
   3541 rows with symptoms or re-intervention event
   31 rows with time=0 -> 0.5
   17 events with time=0 -> 0.5
   3.28 ( 2.9 to 3.7 ) years, 95% survival
   0.941 ( 0.939 to 0.943 ) 5 year survival probability

Unconfounded TVT cohort
------------------------
41880 patients in cohort
345 died in hospital
41535 alive at end of study
1389 spells due to relevant symptoms
2476 spells with SUI mesh intervention
41880 patients in SUI cohort with index procedure
64022335 total follow-up (days), 175283.6 years
1528.7 days mean follow-up, 4.2 years
1541 days median follow-up, 4.2 years
   89.79 % followed up for > 1 year(s)
   77.91 % followed up for > 2 year(s)
   65.81 % followed up for > 3 year(s)
   52.97 % followed up for > 4 year(s)
   40.5 % followed up for > 5 year(s)
   27.44 % followed up for > 6 year(s)
   13.91 % followed up for > 7 year(s)
   0.01 % followed up for > 8 year(s)

Relevant symptom events:
   43269 total events
   41880 unique patients
   1389 admissions for symptom events
   7.92 per 1000 person years crude IR
   1047 unique patients
  Distribution of admissions for relevant symptoms:
    n  freq
    0 40833
```

```
    1   779
    2   211
    3    42
    4    14
    6     1
  Breakdown of admissions for symptoms:
     0 pain ( 0 patients)
     0 bleeding ( 0 patients)
     0 complication ( 0 patients)
     1389 device ( 1047 patients)

Further SUI interventions:
   44356 total events
   41880 unique patients
   2476 admissions for reintervention events
   14.13 per 1000 person years crude IR
   2016 unique patients
   Distribution of admissions for further mesh interventions:
    n  freq
    0 39864
    1  1647
    2   294
    3    63
    4     9
    5     2
    6     1
  Breakdown of further mesh interventions:
     831 introductions ( 812 patients)
     459 repairs ( 425 patients)
     1277 removals ( 1112 patients)
     5 renewals ( 5 patients)

Cross-tabulation of symptoms and re-intervention:
        Reop
Symptoms     0     1
       0 39633  1200
       1   232   815

Re-intervention or symptoms event analysis:
   2950 admissions for relevant events (in unique spells)
   2248 patients
   16.83 per 1000 person years crude IR
   Distribution of admissions for relevant events:
    n  freq
    0 39632
    1  1737
    2   375
    3    98
    4    25
    5     9
    6     4

First relevant symptoms event analysis:
   1389 events after first event  (e.g. censor after first admission for relevant symptoms)
   41880 first events
   41880 unique patients
   1047 rows with symptom event
   9 rows with time=0 -> 0.5
   0 events with time=0 -> 0.5
   NA ( NA to NA ) years, 95% survival
   0.972 ( 0.97 to 0.974 ) 5 year survival probability

First re-intervention event analysis:
   2476 events after first event (e.g. censor after re-intervention)
   41880 first events
   41880 unique patients
   2015 rows with re-intervention event
   17 rows with time=0 -> 0.5
   8 events with time=0 -> 0.5
   4 ( 3.47 to 4.45 ) years, 95% survival
   0.945 ( 0.943 to 0.948 ) 5 year survival probability

First relevant event survival analysis:
```

```
   2950 events after first event  (e.g. censor after first symptoms or re-intervention)
   41880 first events
   41880 unique patients
   2247 rows with symptoms or re-intervention event
   17 rows with time=0 -> 0.5
   8 events with time=0 -> 0.5
   2.65 ( 2.32 to 3.22 ) years, 95% survival
   0.939 ( 0.937 to 0.942 ) 5 year survival probability

Unconfounded TOT cohort
------------------------
25509 patients in cohort
204 died in hospital
25305 alive at end of study
607 spells due to relevant symptoms
1370 spells with SUI mesh intervention
25509 patients in SUI cohort with index procedure
39570960 total follow-up (days), 108339.4 years
1551.3 days mean follow-up, 4.2 years
1607 days median follow-up, 4.4 years
   90.77 % followed up for > 1 year(s)
   79.26 % followed up for > 2 year(s)
   67.62 % followed up for > 3 year(s)
   55.07 % followed up for > 4 year(s)
   41.44 % followed up for > 5 year(s)
   27.55 % followed up for > 6 year(s)
   12.92 % followed up for > 7 year(s)
   0 % followed up for > 8 year(s)

Relevant symptom events:
   26116 total events
   25509 unique patients
   607 admissions for symptom events
   5.6 per 1000 person years crude IR
   484 unique patients
  Distribution of admissions for relevant symptoms:
    n  freq
    0 25025
    1   390
    2    72
    3    17
    4     3
    5     2
  Breakdown of admissions for symptoms:
     0 pain ( 0 patients)
     0 bleeding ( 0 patients)
     0 complication ( 0 patients)
     607 device ( 484 patients)

Further SUI interventions:
   26879 total events
   25509 unique patients
   1370 admissions for reintervention events
   12.65 per 1000 person years crude IR
   1161 unique patients
   Distribution of admissions for further mesh interventions:
    n  freq
    0 24348
    1   995
    2   132
    3    26
    4     7
    5     1
  Breakdown of further mesh interventions:
     630 introductions ( 615 patients)
     240 repairs ( 227 patients)
     541 removals ( 486 patients)
     1 renewals ( 1 patients)

Cross-tabulation of symptoms and re-intervention:
        Reop
Symptoms     0     1
       0 24254   771
```

```
      1    94   390

Re-intervention or symptoms event analysis:
   1580 admissions for relevant events (in unique spells)
   1255 patients
   14.58 per 1000 person years crude IR
   Distribution of admissions for relevant events:
    n  freq
    0 24254
    1  1017
    2   174
    3    47
    4    12
    5     4
    6     1

First relevant symptoms event analysis:
   607 events after first event  (e.g. censor after first admission for relevant symptoms)
   25509 first events
   25509 unique patients
   484 rows with symptom event
   5 rows with time=0 -> 0.5
   0 events with time=0 -> 0.5
   NA ( NA to NA ) years, 95% survival
   0.978 ( 0.976 to 0.98 ) 5 year survival probability

First re-intervention event analysis:
   1370 events after first event (e.g. censor after re-intervention)
   25509 first events
   25509 unique patients
   1161 rows with re-intervention event
   13 rows with time=0 -> 0.5
   8 events with time=0 -> 0.5
   4.75 ( 4.07 to 5.3 ) years, 95% survival
   0.948 ( 0.945 to 0.952 ) 5 year survival probability

First relevant event survival analysis:
   1580 events after first event  (e.g. censor after first symptoms or re-intervention)
   25509 first events
   25509 unique patients
   1255 rows with symptoms or re-intervention event
   13 rows with time=0 -> 0.5
   8 events with time=0 -> 0.5
   4 ( 3.58 to 4.6 ) years, 95% survival
   0.944 ( 0.941 to 0.948 ) 5 year survival probability

Unconfounded minisling cohort
-----------------------------
613 patients in cohort
3 died in hospital
610 alive at end of study
12 spells due to relevant symptoms
40 spells with SUI mesh intervention
613 patients in SUI cohort with index procedure
968081 total follow-up (days), 2650.461 years
1579.3 days mean follow-up, 4.3 years
1644 days median follow-up, 4.5 years
   91.03 % followed up for > 1 year(s)
   75.2 % followed up for > 2 year(s)
   64.44 % followed up for > 3 year(s)
   54.65 % followed up for > 4 year(s)
   44.7 % followed up for > 5 year(s)
   32.79 % followed up for > 6 year(s)
   19.9 % followed up for > 7 year(s)
   0.16 % followed up for > 8 year(s)

Relevant symptom events:
   625 total events
   613 unique patients
   12 admissions for symptom events
   4.53 per 1000 person years crude IR
   10 unique patients
  Distribution of admissions for relevant symptoms:
```

```
    n freq
    0  603
    1    9
    3    1
  Breakdown of admissions for symptoms:
     0 pain ( 0 patients)
     0 bleeding ( 0 patients)
     0 complication ( 0 patients)
     12 device ( 10 patients)

Further SUI interventions:
   653 total events
   613 unique patients
   40 admissions for reintervention events
   15.09 per 1000 person years crude IR
   36 unique patients
   Distribution of admissions for further mesh interventions:
    n freq
    0  577
    1   32
    2    4
  Breakdown of further mesh interventions:
     25 introductions ( 24 patients)
     6 repairs ( 5 patients)
     11 removals ( 10 patients)
     0 renewals ( 0 patients)

Cross-tabulation of symptoms and re-intervention:
        Reop
Symptoms   0   1
       0 574  29
       1   3   7

Re-intervention or symptoms event analysis:
   45 admissions for relevant events (in unique spells)
   39 patients
   16.98 per 1000 person years crude IR
   Distribution of admissions for relevant events:
    n freq
    0  574
    1   34
    2    4
    3    1

First relevant symptoms event analysis:
   12 events after first event  (e.g. censor after first admission for relevant symptoms)
   613 first events
   613 unique patients
   10 rows with symptom event
   0 rows with time=0 -> 0.5
   0 events with time=0 -> 0.5
   NA ( NA to NA ) years, 95% survival
   0.983 ( 0.972 to 0.995 ) 5 year survival probability

First re-intervention event analysis:
   40 events after first event (e.g. censor after re-intervention)
   613 first events
   613 unique patients
   36 rows with re-intervention event
   1 rows with time=0 -> 0.5
   1 events with time=0 -> 0.5
   3.27 ( 1.21 to 5.12 ) years, 95% survival
   0.929 ( 0.905 to 0.953 ) 5 year survival probability

First relevant event survival analysis:
   45 events after first event  (e.g. censor after first symptoms or re-intervention)
   613 first events
   613 unique patients
   39 rows with symptoms or re-intervention event
   1 rows with time=0 -> 0.5
   1 events with time=0 -> 0.5
   2.09 ( 0.92 to 4.87 ) years, 95% survival
   0.925 ( 0.901 to 0.949 ) 5 year survival probability
```

```
Confounded cohort
-----------------
24244 patients in cohort
214 died in hospital
24030 alive at end of study
817 spells due to relevant symptoms
1506 spells with SUI mesh intervention
24244 patients in SUI cohort with index procedure
37672865 total follow-up (days), 103142.7 years
1553.9 days mean follow-up, 4.3 years
1603 days median follow-up, 4.4 years
   91.2 % followed up for > 1 year(s)
   79.65 % followed up for > 2 year(s)
   67.95 % followed up for > 3 year(s)
   55.08 % followed up for > 4 year(s)
   41.13 % followed up for > 5 year(s)
   27.05 % followed up for > 6 year(s)
   13.19 % followed up for > 7 year(s)
   0.11 % followed up for > 8 year(s)

Relevant symptom events:
   25061 total events
   24244 unique patients
   817 admissions for symptom events
   7.92 per 1000 person years crude IR
   640 unique patients
  Distribution of admissions for relevant symptoms:
    n  freq
    0 23604
    1   501
    2   109
    3    23
    4     6
    5     1
  Breakdown of admissions for symptoms:
     1 pain ( 1 patients)
     0 bleeding ( 0 patients)
     0 complication ( 0 patients)
     817 device ( 640 patients)

Further SUI interventions:
   25750 total events
   24244 unique patients
   1506 admissions for reintervention events
   14.6 per 1000 person years crude IR
   1238 unique patients
   Distribution of admissions for further mesh interventions:
    n  freq
    0 23006
    1  1015
    2   191
    3    24
    4     5
    5     1
    6     2
  Breakdown of further mesh interventions:
     494 introductions ( 478 patients)
     263 repairs ( 247 patients)
     778 removals ( 683 patients)
     2 renewals ( 2 patients)

Cross-tabulation of symptoms and re-intervention:
        Reop
Symptoms     0     1
       0 22859   745
       1   147   493

Re-intervention or symptoms event analysis:
   1780 admissions for relevant events (in unique spells)
   1385 patients
   17.26 per 1000 person years crude IR
   Distribution of admissions for relevant events:
```

```
    n  freq
    0 22859
    1  1083
    2   236
    3    46
    4    15
    5     3
    6     2

First relevant symptoms event analysis:
   817 events after first event  (e.g. censor after first admission for relevant symptoms)
   24244 first events
   24244 unique patients
   640 rows with symptom event
   2 rows with time=0 -> 0.5
   0 events with time=0 -> 0.5
   NA ( NA to NA ) years, 95% survival
   0.97 ( 0.968 to 0.973 ) 5 year survival probability

First re-intervention event analysis:
   1506 events after first event (e.g. censor after re-intervention)
   24244 first events
   24244 unique patients
   1238 rows with re-intervention event
   10 rows with time=0 -> 0.5
   8 events with time=0 -> 0.5
   3.3 ( 2.64 to 3.99 ) years, 95% survival
   0.942 ( 0.939 to 0.945 ) 5 year survival probability

First relevant event survival analysis:
   1780 events after first event  (e.g. censor after first symptoms or re-intervention)
   24244 first events
   24244 unique patients
   1385 rows with symptoms or re-intervention event
   10 rows with time=0 -> 0.5
   8 events with time=0 -> 0.5
   2.3 ( 1.94 to 2.71 ) years, 95% survival
   0.935 ( 0.932 to 0.939 ) 5 year survival probability

Confounded TVT, TOT or MS
-------------------------
24184 patients in cohort
213 died in hospital
23971 alive at end of study
814 spells due to relevant symptoms
1498 spells with SUI mesh intervention
24184 patients in SUI cohort with index procedure
37573381 total follow-up (days), 102870.3 years
1553.6 days mean follow-up, 4.3 years
1603 days median follow-up, 4.4 years
   91.21 % followed up for > 1 year(s)
   79.64 % followed up for > 2 year(s)
   67.93 % followed up for > 3 year(s)
   55.07 % followed up for > 4 year(s)
   41.12 % followed up for > 5 year(s)
   27.04 % followed up for > 6 year(s)
   13.17 % followed up for > 7 year(s)
   0.11 % followed up for > 8 year(s)

Relevant symptom events:
   24998 total events
   24184 unique patients
   814 admissions for symptom events
   7.91 per 1000 person years crude IR
   638 unique patients
  Distribution of admissions for relevant symptoms:
    n  freq
    0 23546
    1   500
    2   108
    3    23
    4     6
    5     1
```

```
  Breakdown of admissions for symptoms:
     1 pain ( 1 patients)
     0 bleeding ( 0 patients)
     0 complication ( 0 patients)
     814 device ( 638 patients)

Further SUI interventions:
   25682 total events
   24184 unique patients
   1498 admissions for reintervention events
   14.56 per 1000 person years crude IR
   1231 unique patients
   Distribution of admissions for further mesh interventions:
    n  freq
    0 22953
    1  1009
    2   190
    3    24
    4     5
    5     1
    6     2
  Breakdown of further mesh interventions:
     491 introductions ( 475 patients)
     262 repairs ( 246 patients)
     774 removals ( 679 patients)
     2 renewals ( 2 patients)

Cross-tabulation of symptoms and re-intervention:
        Reop
Symptoms     0     1
       0 22807   739
       1   146   492

Re-intervention or symptoms event analysis:
   1770 admissions for relevant events (in unique spells)
   1377 patients
   17.21 per 1000 person years crude IR
   Distribution of admissions for relevant events:
    n  freq
    0 22807
    1  1077
    2   234
    3    46
    4    15
    5     3
    6     2

First relevant symptoms event analysis:
   814 events after first event  (e.g. censor after first admission for relevant symptoms)
   24184 first events
   24184 unique patients
   638 rows with symptom event
   2 rows with time=0 -> 0.5
   0 events with time=0 -> 0.5
   NA ( NA to NA ) years, 95% survival
   0.97 ( 0.968 to 0.973 ) 5 year survival probability

First re-intervention event analysis:
   1498 events after first event (e.g. censor after re-intervention)
   24184 first events
   24184 unique patients
   1231 rows with re-intervention event
   10 rows with time=0 -> 0.5
   8 events with time=0 -> 0.5
   3.33 ( 2.68 to 4.03 ) years, 95% survival
   0.942 ( 0.939 to 0.946 ) 5 year survival probability

First relevant event survival analysis:
   1770 events after first event  (e.g. censor after first symptoms or re-intervention)
   24184 first events
   24184 unique patients
   1377 rows with symptoms or re-intervention event
   10 rows with time=0 -> 0.5
```

```
   8 events with time=0 -> 0.5
   2.34 ( 1.94 to 2.72 ) years, 95% survival
   0.936 ( 0.932 to 0.939 ) 5 year survival probability

Confounded TVT cohort
------------------------
14768 patients in cohort
131 died in hospital
14637 alive at end of study
506 spells due to relevant symptoms
873 spells with SUI mesh intervention
14768 patients in SUI cohort with index procedure
22675836 total follow-up (days), 62083.06 years
1535.5 days mean follow-up, 4.2 years
1565 days median follow-up, 4.3 years
   90.68 % followed up for > 1 year(s)
   78.72 % followed up for > 2 year(s)
   66.68 % followed up for > 3 year(s)
   53.6 % followed up for > 4 year(s)
   40.37 % followed up for > 5 year(s)
   26.79 % followed up for > 6 year(s)
   13.48 % followed up for > 7 year(s)
   0.12 % followed up for > 8 year(s)

Relevant symptom events:
   15274 total events
   14768 unique patients
   506 admissions for symptom events
   8.15 per 1000 person years crude IR
   395 unique patients
  Distribution of admissions for relevant symptoms:
    n  freq
    0 14373
    1   304
    2    75
    3    12
    4     4
  Breakdown of admissions for symptoms:
     0 pain ( 0 patients)
     0 bleeding ( 0 patients)
     0 complication ( 0 patients)
     506 device ( 395 patients)

Further SUI interventions:
   15641 total events
   14768 unique patients
   873 admissions for reintervention events
   14.06 per 1000 person years crude IR
   722 unique patients
   Distribution of admissions for further mesh interventions:
    n  freq
    0 14046
    1   592
    2   113
    3    14
    4     2
    5     1
  Breakdown of further mesh interventions:
     238 introductions ( 233 patients)
     143 repairs ( 138 patients)
     506 removals ( 448 patients)
     2 renewals ( 2 patients)

Cross-tabulation of symptoms and re-intervention:
        Reop
Symptoms     0     1
       0 13966   407
       1    80   315

Re-intervention or symptoms event analysis:
   1026 admissions for relevant events (in unique spells)
   802 patients
   16.53 per 1000 person years crude IR
```

```
   Distribution of admissions for relevant events:
    n  freq
    0 13966
    1   625
    2   141
    3    27
    4     7
    5     2

First relevant symptoms event analysis:
   506 events after first event  (e.g. censor after first admission for relevant symptoms)
   14768 first events
   14768 unique patients
   395 rows with symptom event
   1 rows with time=0 -> 0.5
   0 events with time=0 -> 0.5
   NA ( NA to NA ) years, 95% survival
   0.971 ( 0.968 to 0.974 ) 5 year survival probability

First re-intervention event analysis:
   873 events after first event (e.g. censor after re-intervention)
   14768 first events
   14768 unique patients
   722 rows with re-intervention event
   5 rows with time=0 -> 0.5
   4 events with time=0 -> 0.5
   3.84 ( 2.84 to 4.69 ) years, 95% survival
   0.945 ( 0.941 to 0.949 ) 5 year survival probability

First relevant event survival analysis:
   1026 events after first event  (e.g. censor after first symptoms or re-intervention)
   14768 first events
   14768 unique patients
   802 rows with symptoms or re-intervention event
   5 rows with time=0 -> 0.5
   4 events with time=0 -> 0.5
   2.49 ( 1.92 to 3.45 ) years, 95% survival
   0.939 ( 0.935 to 0.944 ) 5 year survival probability

Confounded TOT cohort
------------------------
9195 patients in cohort
80 died in hospital
9115 alive at end of study
301 spells due to relevant symptoms
619 spells with SUI mesh intervention
9195 patients in SUI cohort with index procedure
14556153 total follow-up (days), 39852.57 years
1583.1 days mean follow-up, 4.3 years
1650 days median follow-up, 4.5 years
   92.13 % followed up for > 1 year(s)
   81.21 % followed up for > 2 year(s)
   70.06 % followed up for > 3 year(s)
   57.46 % followed up for > 4 year(s)
   42.31 % followed up for > 5 year(s)
   27.36 % followed up for > 6 year(s)
   12.58 % followed up for > 7 year(s)
   0.08 % followed up for > 8 year(s)

Relevant symptom events:
   9496 total events
   9195 unique patients
   301 admissions for symptom events
   7.55 per 1000 person years crude IR
   237 unique patients
  Distribution of admissions for relevant symptoms:
    n freq
    0 8958
    1  191
    2   32
    3   11
    4    2
    5    1
```

```
  Breakdown of admissions for symptoms:
     1 pain ( 1 patients)
     0 bleeding ( 0 patients)
     0 complication ( 0 patients)
     301 device ( 237 patients)

Further SUI interventions:
   9814 total events
   9195 unique patients
   619 admissions for reintervention events
   15.53 per 1000 person years crude IR
   503 unique patients
   Distribution of admissions for further mesh interventions:
    n freq
    0 8692
    1  411
    2   77
    3   10
    4    3
    6    2
  Breakdown of further mesh interventions:
     250 introductions ( 239 patients)
     118 repairs ( 107 patients)
     266 removals ( 229 patients)
     0 renewals ( 0 patients)

Cross-tabulation of symptoms and re-intervention:
        Reop
Symptoms    0    1
       0 8631  327
       1   61  176

Re-intervention or symptoms event analysis:
   732 admissions for relevant events (in unique spells)
   564 patients
   18.37 per 1000 person years crude IR
   Distribution of admissions for relevant events:
    n freq
    0 8631
    1  442
    2   92
    3   19
    4    8
    5    1
    6    2

First relevant symptoms event analysis:
   301 events after first event  (e.g. censor after first admission for relevant symptoms)
   9195 first events
   9195 unique patients
   237 rows with symptom event
   1 rows with time=0 -> 0.5
   0 events with time=0 -> 0.5
   NA ( NA to NA ) years, 95% survival
   0.971 ( 0.967 to 0.974 ) 5 year survival probability

First re-intervention event analysis:
   619 events after first event (e.g. censor after re-intervention)
   9195 first events
   9195 unique patients
   503 rows with re-intervention event
   5 rows with time=0 -> 0.5
   4 events with time=0 -> 0.5
   2.66 ( 2.03 to 3.55 ) years, 95% survival
   0.937 ( 0.932 to 0.943 ) 5 year survival probability

First relevant event survival analysis:
   732 events after first event  (e.g. censor after first symptoms or re-intervention)
   9195 first events
   9195 unique patients
   564 rows with symptoms or re-intervention event
   5 rows with time=0 -> 0.5
   4 events with time=0 -> 0.5
```

```
   2.02 ( 1.69 to 2.64 ) years, 95% survival
   0.93 ( 0.924 to 0.936 ) 5 year survival probability

Confounded minisling cohort
-----------------------------
221 patients in cohort
2 died in hospital
219 alive at end of study
7 spells due to relevant symptoms
6 spells with SUI mesh intervention
221 patients in SUI cohort with index procedure
341392 total follow-up (days), 934.6804 years
1544.8 days mean follow-up, 4.2 years
1541 days median follow-up, 4.2 years
   88.69 % followed up for > 1 year(s)
   76.02 % followed up for > 2 year(s)
   62.9 % followed up for > 3 year(s)
   53.85 % followed up for > 4 year(s)
   42.08 % followed up for > 5 year(s)
   30.77 % followed up for > 6 year(s)
   16.74 % followed up for > 7 year(s)
   0.45 % followed up for > 8 year(s)

Relevant symptom events:
   228 total events
   221 unique patients
   7 admissions for symptom events
   7.49 per 1000 person years crude IR
   6 unique patients
  Distribution of admissions for relevant symptoms:
    n freq
    0  215
    1    5
    2    1
  Breakdown of admissions for symptoms:
     0 pain ( 0 patients)
     0 bleeding ( 0 patients)
     0 complication ( 0 patients)
     7 device ( 6 patients)

Further SUI interventions:
   227 total events
   221 unique patients
   6 admissions for reintervention events
   6.42 per 1000 person years crude IR
   6 unique patients
   Distribution of admissions for further mesh interventions:
    n freq
    0  215
    1    6
  Breakdown of further mesh interventions:
     3 introductions ( 3 patients)
     1 repairs ( 1 patients)
     2 removals ( 2 patients)
     0 renewals ( 0 patients)

Cross-tabulation of symptoms and re-intervention:
        Reop
Symptoms   0   1
       0 210   5
       1   5   1

Re-intervention or symptoms event analysis:
   12 admissions for relevant events (in unique spells)
   11 patients
   12.84 per 1000 person years crude IR
   Distribution of admissions for relevant events:
    n freq
    0  210
    1   10
    2    1

First relevant symptoms event analysis:
```

```
   7 events after first event  (e.g. censor after first admission for relevant symptoms)
   221 first events
   221 unique patients
   6 rows with symptom event
   0 rows with time=0 -> 0.5
   0 events with time=0 -> 0.5
   6.58 ( 4.52 to NA ) years, 95% survival
   0.96 ( 0.925 to 0.996 ) 5 year survival probability

First re-intervention event analysis:
   6 events after first event (e.g. censor after re-intervention)
   221 first events
   221 unique patients
   6 rows with re-intervention event
   0 rows with time=0 -> 0.5
   0 events with time=0 -> 0.5
   NA ( 4.73 to NA ) years, 95% survival
   0.971 ( 0.945 to 0.998 ) 5 year survival probability

First relevant event survival analysis:
   12 events after first event  (e.g. censor after first symptoms or re-intervention)
   221 first events
   221 unique patients
   11 rows with symptoms or re-intervention event
   0 rows with time=0 -> 0.5
   0 events with time=0 -> 0.5
   4.53 ( 2.63 to NA ) years, 95% survival
   0.931 ( 0.888 to 0.975 ) 5 year survival probability

-----------------------
overall safety analysis
-----------------------

2866 spells with peri-procedural complication
2180 spells with admission within 30 days for complication
6355 unique spells with long-term admission for surgery or symptoms
0 spells common to peri-procedure and 30 day admissions
0 spells common to 30 day admissions and long-term admission
0 spells common to peri-procedure and long-term admission

Unconfounded cohort:
   68002 patients with first-time mesh insertion
   1618 with peri-procedural complication
   1137 with 30 day readmission for complication
   3542 with post-procedural admission for device or relevant surgery
   2639 with peri-procedural or 30 day complication, 3.88 %
   5915 with at least one of these, 8.7 %

Unconfounded TVT:
   41880 patients with first-time mesh insertion
   1232 with peri-procedural complication
   833 with 30 day readmission for complication
   2248 with post-procedural admission for device or relevant surgery
   1983 with peri-procedural or 30 day complication, 4.73 %
   4028 with at least one of these, 9.62 %

Unconfounded TOT:
   25509 patients with first-time mesh insertion
   356 with peri-procedural complication
   275 with 30 day readmission for complication
   1255 with post-procedural admission for device or relevant surgery
   601 with peri-procedural or 30 day complication, 2.36 %
   1796 with at least one of these, 7.04 %

Unconfounded MS:
   613 patients with first-time mesh insertion
   30 with peri-procedural complication
   29 with 30 day readmission for complication
   39 with post-procedural admission for device or relevant surgery
   55 with peri-procedural or 30 day complication, 8.97 %
   91 with at least one of these, 14.85 %

Confounded cohort:
```

```
   24244 patients with first-time mesh insertion
   1248 with peri-procedural complication
   739 with 30 day readmission for complication
   1385 with post-procedural admission for device or relevant surgery
   1893 with peri-procedural or 30 day complication, 7.81 %
   3105 with at least one of these, 12.81 %

Confounded TVT:
   14768 patients with first-time mesh insertion
   880 with peri-procedural complication
   479 with 30 day readmission for complication
   802 with post-procedural admission for device or relevant surgery
   1295 with peri-procedural or 30 day complication, 8.77 %
   1987 with at least one of these, 13.45 %

Confounded TOT:
   9195 patients with first-time mesh insertion
   342 with peri-procedural complication
   250 with 30 day readmission for complication
   564 with post-procedural admission for device or relevant surgery
   565 with peri-procedural or 30 day complication, 6.14 %
   1068 with at least one of these, 11.62 %

Confounded MS:
   221 patients with first-time mesh insertion
   23 with peri-procedural complication
   9 with 30 day readmission for complication
   11 with post-procedural admission for device or relevant surgery
   29 with peri-procedural or 30 day complication, 13.12 %
   39 with at least one of these, 17.65 %

Combinations:
   60 patients with first-time mesh insertion
   3 with peri-procedural complication
   1 with 30 day readmission for complication
   8 with post-procedural admission for device or relevant surgery
   4 with peri-procedural or 30 day complication, 6.67 %
   11 with at least one of these, 18.33 %


2016-07-14 18:33:18
```

Supplementary Table S7: ICD10 diagnosis codes used to classify peri-procedural complication types ('.x' represents any number from 0 to 9 indicating that the whole chapter is used)

Complication type	*ICD10 codes*
Procedural	T81.x, (S34.5, S34.6, S34.8, S35.7, S35.8, S35.9, S36.5, S36.7, S36.8, S36.9, S37.0, S37.1, S37.2, S37.3, S37.7, S37.8, S37.9, S39.x) qualified by Y60.0 or Y60.8
Device	T83.1, T83.4, T83.5, T83.6, T83.8, T83.9, T85.6, T85.7, T85.8, T85.9, any main code qualified by Y73.2 or Y73.3 or Y83.1 (excluding R30.x-R39.x)
Complication with urinary symptoms	R30.x-R39.x, N99.8, N99.9
Other complications	Any remaining complications identified by ICD10 codes not specified above.

Supplementary Table S8: ICD10 diagnosis codes used in the primary diagnosis position to define admission for complication of mesh device implanted previously

Symptom type	*ICD10 codes*
Complication of device implanted previously	T83.1, T83.4, T83.5, T83.6, T83.7, T83.8, T83.9, any main code qualified by Y73.2 or Y73.3

Supplementary Table S9: The number of reported peri-procedural complications (% of occurrence) for the six most frequently reported overall during admissions for tension-free vaginal (TVT), trans-obturator (TOT) and suprapubic sling (SS) surgical mesh insertion in the absence of concomitant procedure likely to influence outcomes.

ICD10 subchapter	*Description*	*TVT*	*TOT*	*SS*
T81	Complications of procedures not elsewhere classified	643 (50.6)	91 (24.9)	21 (61.8)
R33†	Retention of urine	350 (27.5)	152 (41.6)	3 (8.8)
N99	Post-procedural disorders of genitourinary system not elsewhere classified	46 (3.6)	11 (3.0)	0 (0.0)
T83	Complications of genitourinary prosthetic devices, implants and grafts	26 (2.0)	11 (3.0)	2 (5.9)
S37†	Injury of urinary and pelvic organs	23 (1.8)	2 (0.5)	0 (0.0)
T88	Other complications of surgical and medical care not elsewhere classified	14 (1.1)	9 (2.5)	0 (0.0)
	Total number reported	1271 (100.0)	365 (100.0)	34 (100.0)

†Followed by one or more supplementary codes (from ICD-10 sub-chapter Y)

Supplementary Table S10: The number of reported peri-procedural complications (% of occurrence) for the six most frequently reported overall during admissions for tension-free vaginal (TVT), trans-obturator (TOT) and suprapubic sling (SS) surgical mesh insertion in the presence of concomitant procedures likely to influence outcomes.

ICD10 Subchapter	*Description*	*TVT*	*TOT*	*SS*
T81	Complications of procedures not elsewhere classified	484 (49.4)	166 (45.7)	20 (62.5)
R33†	Retention of urine	185 (18.9)	86 (23.7)	2 (6.3)
T83	Complications of genitourinary prosthetic devices, implants and grafts	41 (4.2)	16 (4.4)	3 (9.4)
N99	Post-procedural disorders of genitourinary system not elsewhere classified	40 (4.1)	16 (4.4)	0 (0.0)
N39†	Other disorders of urinary system	29 (3.0)	10 (2.8)	1 (3.1)
T88	Other complications of surgical and medical care not elsewhere classified	12 (1.2)	5 (1.4)	0 (0.0)
	Total number reported	979 (100.0)	363 (100.0)	32 (100.0)

†Followed by one or more supplementary codes (from ICD-10 sub-chapter Y)

Supplementary Table S11: Readmissions [patients, %] occurring within 30-days of index admissions for surgical mesh insertions conducted in the absence of concomitant procedures likely to influence outcomes.

	TVT		*TOT*		*SS*	
Total patients		41,880		25,509		613
Total readmissions	4025	[3255, 7.8]	1749	[1485, 5.8]	130	[110,17.9]
Routine readmission	3094	[2580, 6.1]	1440	[1257, 4.9]	97	[85, 13.9]
- catheter intervention	*1369*		*546*		*50*	
- no intervention	*970*		*417*		*28*	
- diagnostic/ imaging only	*212*		*149*		*7*	
- further mesh surgery	*138*		*62*		*3*	
Readmission for complications	931	[833, 2.0]	309	[275, 1.1]	33	[29, 4.7]
- procedural	*253*		*86*		*18*	
- device	*233*		*81*		*5*	
- urinary complications	*340*		*99*		*6*	
- other complications	*151*		*60*		*6*	
Readmission for complications and with further surgery	144	[141, 16.9†]	31	[30, 10.9†]	0	[0, 0.0]

†Percentage of patients readmitted for complications

Supplementary Table S12: Readmissions [patients, %] occurring within 30-days of index admissions for surgical mesh insertions conducted in the presence of concomitant procedures likely to influence outcomes.

	TVT		*TOT*		*SS*	
Total patients		14,768		9195		221
Total readmissions	1898	[1502, 10.2]	930	[785, 8.5]	70	[54,24.4]
Routine readmission	1369	[1102, 7.5]	662	[578, 6.3]	60	[46, 20.8]
- catheter intervention	*633*		*276*		*33*	
- no intervention	*476*		*247*		*19*	
- diagnostic/ imaging only	*81*		*49*		*3*	
- further mesh surgery	*58*		*23*		*1*	
Readmission for complications	529	[479, 3.2]	268	[250, 2.7]	10	[9, 4.1]
- procedural	*256*		*145*		*6*	
- device	*89*		*42*		*1*	
- urinary complications	*112*		*47*		*2*	
- other complications	*93*		*44*		*2*	
Readmission for complications and with further surgery	61	[61, 12.7†]	22	[22, 8.8†]	1	[1, 10.0†]

†Percentage of patients readmitted for complications

Supplementary Figure S13: Kaplan-Meier curve for time to first readmission for further mesh surgery or for complications from previous mesh surgery (solid lines) and 95% confidence limits (dashed lines) for those having TVT mesh insertions in the absence of concomitant procedures likely to influence outcome. Dash-dot lines indicate the proportions of patients free from mesh-related admission at 5 years (with 95% confidence intervals.

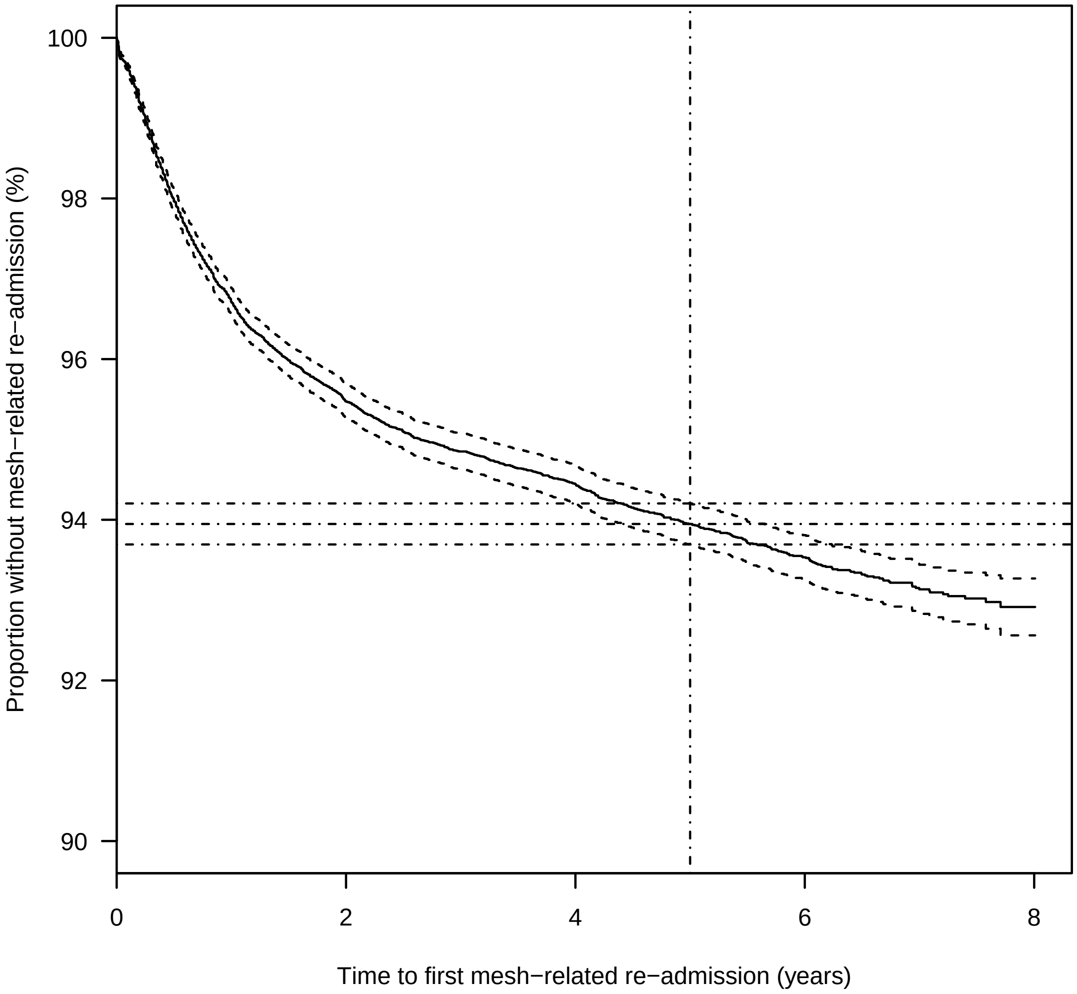

Supplementary Figure S14: Kaplan-Meier curve for time to first readmission for further mesh surgery or for complications from previous mesh surgery (solid lines) and 95% confidence limits (dashed lines) for those having TOT mesh insertion in the absence of concomitant procedures likely to influence outcomes. Dash-dot lines indicate the proportions of patients free from mesh-related admission at 5 years (with 95% confidence intervals).

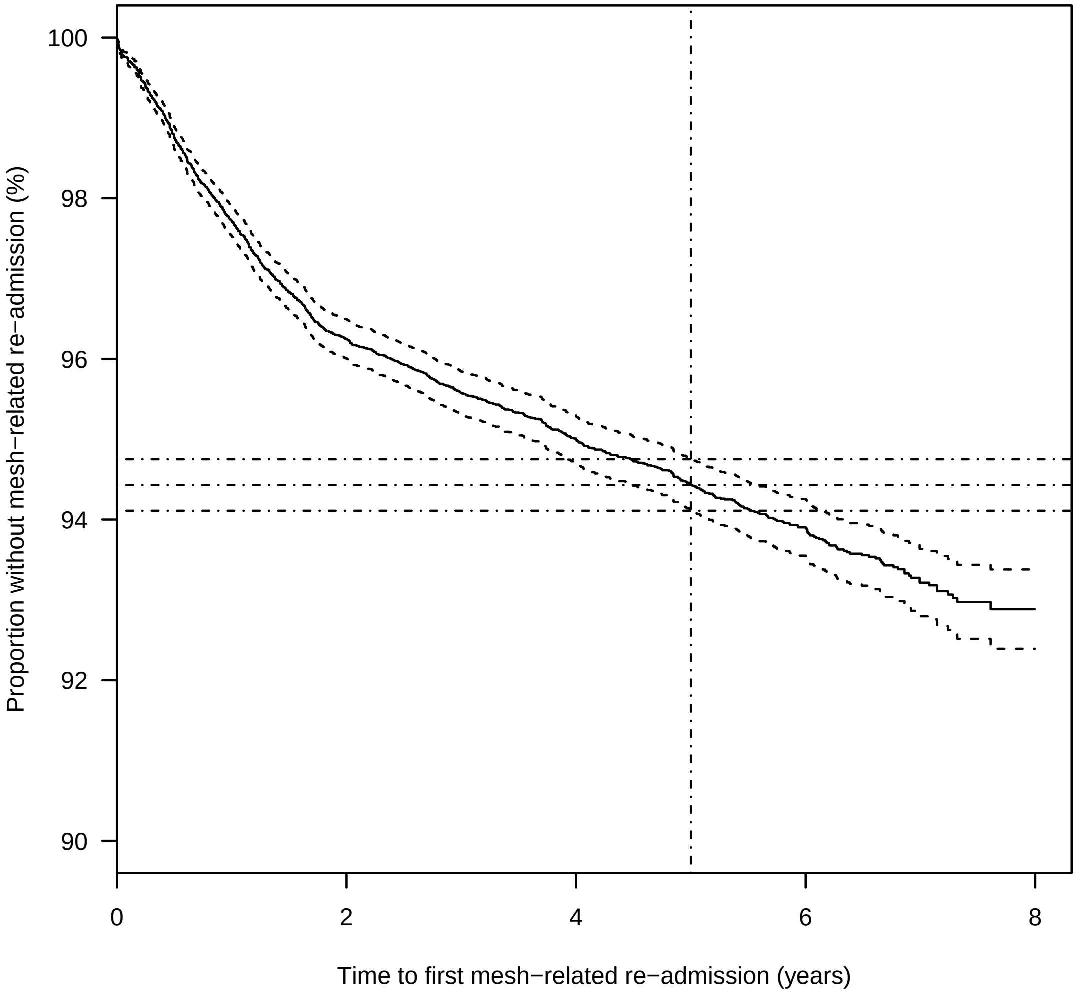

Supplementary Figure S15: Kaplan-Meier curve for time to first readmission for further mesh surgery or for complications from previous mesh surgery (solid lines) and 95% confidence limits (dashed lines) for those having SS mesh insertion in the absence of concomitant procedures likely to influence outcomes. Dash-dot lines indicate the proportions of patients free from mesh-related admission at 5 years (with 95% confidence intervals).

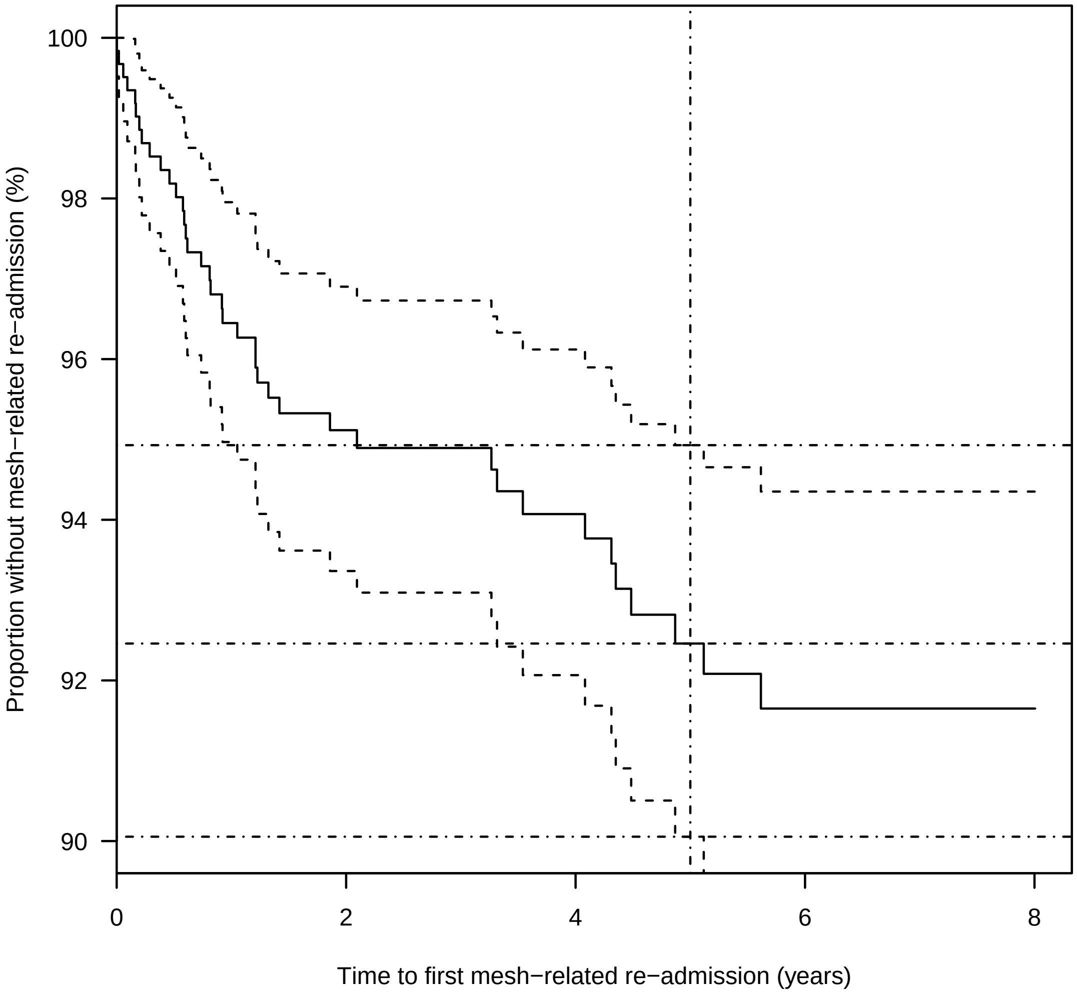

Supplementary Table S16: Cross-tabulations of the number (%) of patients having surgical mesh insertion, in the absence of concomitant procedures likely to influence outcomes, who were readmitted for further mesh surgery, or readmitted for complications of previous mesh surgery, during the study period.

TVT cohort (n=41,880)		Readmitted for complications of previous mesh surgery?	
		No	Yes
Readmitted for further mesh surgery?	No	39,633 (94.6)	232 (0.6)
	Yes	1200 (2.9)	815 (1.9)

TOT cohort (n=25,509)		Readmitted for complications of previous mesh surgery?	
		No	Yes
Readmitted for further mesh surgery?	No	24,254 (95.1)	94 (0.4)
	Yes	771 (3.0)	390 (1.5)

SS cohort (n=613)		Readmitted for complications of previous mesh surgery?	
		No	Yes
Readmitted for further mesh surgery?	No	574 (93.6)	3 (0.5)
	Yes	29 (4.7)	7 (1.1)

All (combined) (n=68,002)		Readmitted for complications of previous mesh surgery?	
		No	Yes
Readmitted for further mesh surgery?	No	64,461 (94.8)	329 (0.5)
	Yes	2000 (2.9)	1212 (1.8)

Supplementary Table S17: The total number of patients (%, percentage of cohort) who had a transvaginal tape (TVT), transobturator tape (TOT) or suprapubic sling (SS) mesh insertion, in the presence of concomitant procedures, who were re-admitted during the study period for further mesh surgery or due to complications from previous mesh surgery. Results are uncorrected for censoring. For example, 802 of 14,768 (5.4%) patients who had a TVT mesh inserted were re-admitted at least once during the period of follow-up.

Procedure type	Number of readmissions				Maximum number of readmissions
	0	1	2	3+	
TVT	13,966 (94.6)	625 (4.2)	141 (1.0)	36 (0.2)	5
TOT	8631 (93.9)	442 (4.8)	92 (1.0)	30 (0.3)	6
SS	210 (95.0)	10 (4.5)	1 (0.5)	0 (0.0)	2
All (combined)	22,807 (94.3)	1077 (4.4)	234 (1.0)	66 (0.3)	6

Supplementary Table S18: Reasons and frequency of readmission, following mesh insertion in the presence of concomitant procedures, during follow-up. The total number (%, percentage of cohort) of patients readmitted is also given (some patients were readmitted on multiple occasions).

	TVT		TOT		SS		All	
	Total	Patients	Total	Patients	Total	Patients	Total	Patients
Total number of patients in cohort (total duration of follow-up)		14,768 (62,083 patient years)		9195 (39,852 patient years)		221 (934 patient years)		24,184 (102,870 patient years)
In-hospital deaths	-	131 (0.9%)	-	80 (0.9%)	-	2 (0.9%)	-	213 (0.9%)
**Readmissions** for further surgery:	873	722 (4.9%)	619	503 (5.5%)	6	6 (2.7%)	1498	1231 (5.1%)
- *removal*	*506*	*448 (3.0%)*	*266*	*229 (2.5%)*	*2*	*2 (0.9%)*	*774*	*679 (2.8%)*
- *repair*	*143*	*138 (0.9%)*	*118*	*107 (1.2%)*	*1*	*1 (0.5%)*	*262*	*246 (1.0%)*
- *insertion*	*238*	*233 (1.6%)*	*250*	*239 (2.6%)*	*3*	*3 (1.4%)*	*491*	*475 (2.0%)*
- *renewal*	*2*	*2 (0.0%)*	*0*	*0 (0.0%)*	*0*	*0 (0.0%)*	*2*	*2 (0.0%)*
**Readmissions** for complications from mesh surgery	506	395 (2.7%)	301	237 (2.6%)	7	6 (2.7%)	814	638 (2.6%)
**Readmissions** for complications *or* further surgery	1026	802 (5.4%)	732	564 (6.1%)	12	11 (5.0%)	1770	1377 (5.7%)
**Readmissions** / 1000 person years:								
- further mesh surgery	14.1	-	15.5	-	6.4	-	14.6	-
- complications of mesh surgery	8.2	-	7.6	-	7.5	-	7.9	-
- complications or further surgery	16.5	-	18.4	-	12.8	-	17.2	-
**Patients** free from further surgery or admission for complications after 5 years [95% CI]		93.9 [93.5-94.4] %		93.0 [92.4-93.6] %		93.1 [88.8-97.5] %		93.6 [93.2-93.9] %

Supplementary Figure S19: Hazard rate for readmission for further mesh surgery or for readmission due to complications from previous mesh surgery in those having surgical mesh insertion in the presence of concomitant procedures likely to influence outcomes. The instantaneous rate is in units of events per 1000 patient years.

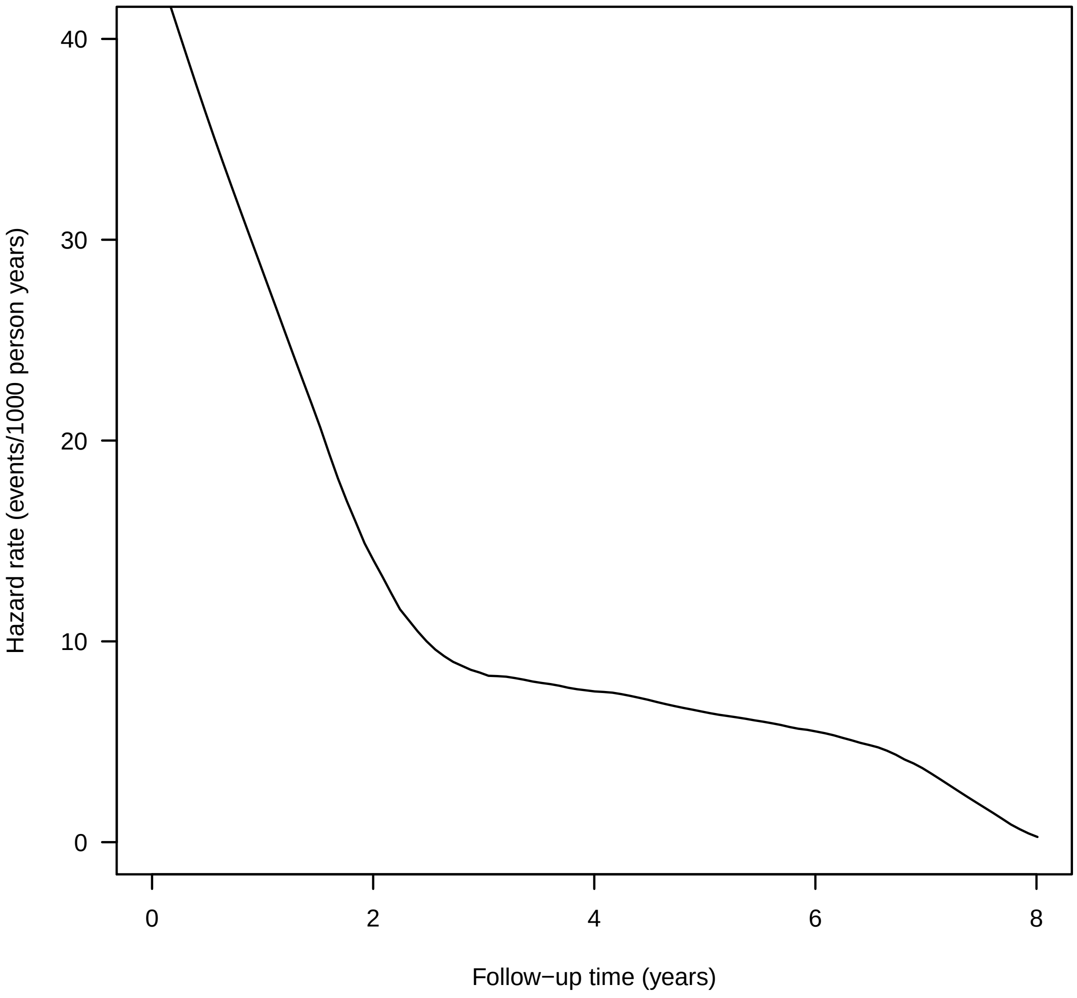

Supplementary Figure S20: Kaplan-Meier curve for time to first readmission for further mesh surgery or for complications from previous mesh surgery (solid lines) and 95% confidence limits (dashed lines) for those having surgical mesh insertion in the presence of concomitant procedures likely to influence outcomes. Dash-dot lines indicate the proportions of patients free from mesh-related admission at 5 years (with 95% confidence intervals).

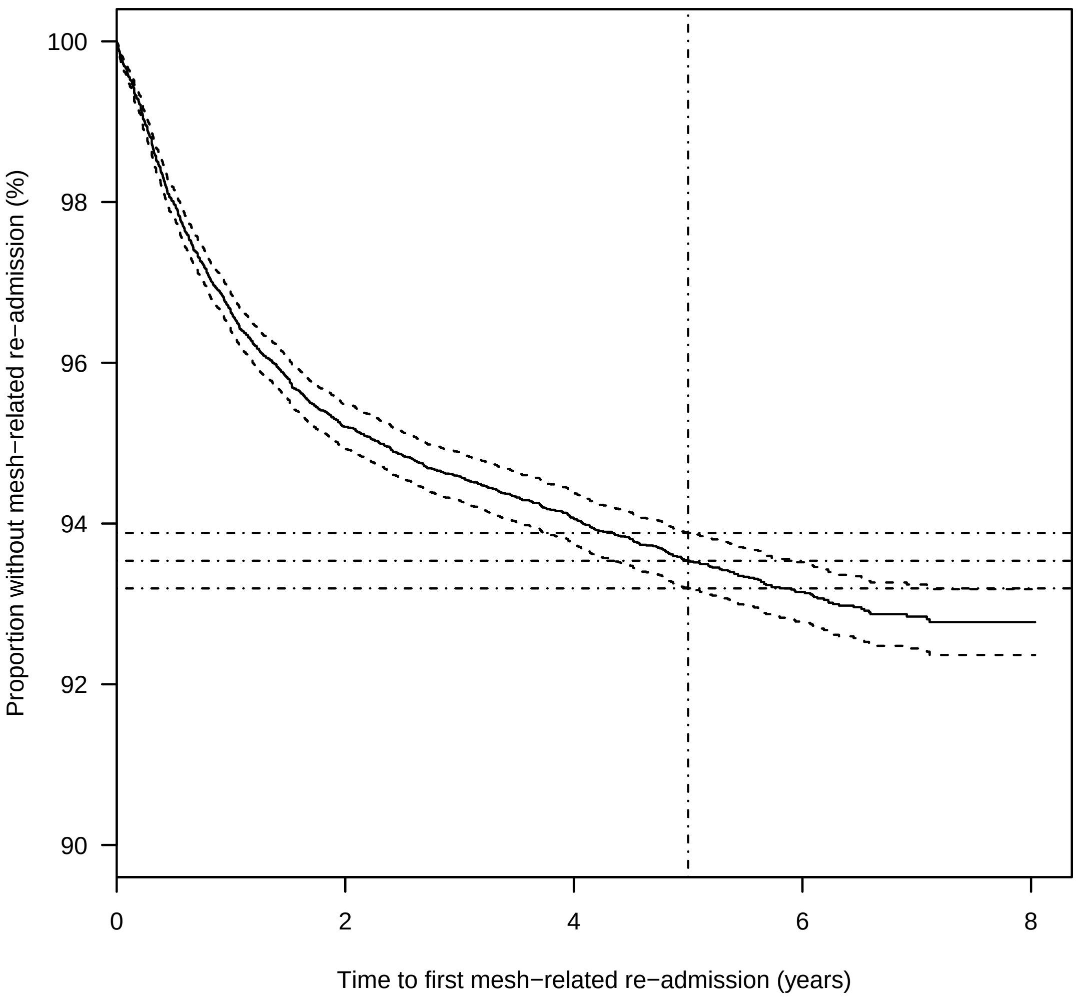

Supplement: Supplementary file 1 — Supplementary Information [file 41598_2017_11821_MOESM1_ESM.pdf]
